# Distinct spatiotemporal contribution of morphogenetic events and mechanical tissue coupling during *Xenopus* neural tube closure

**DOI:** 10.1242/dev.200358

**Published:** 2022-07-01

**Authors:** Neophytos Christodoulou, Paris A. Skourides

**Affiliations:** Department of Biological Sciences, University of Cyprus, P.O. Box 20537, 2109 Nicosia, Cyprus

**Keywords:** Apical constriction, Convergent extension, Morphogenesis, Neural tube closure, Neurulation, *Xenopus*

## Abstract

Neural tube closure (NTC) is a fundamental process during vertebrate development and is indispensable for the formation of the central nervous system. Here, using *Xenopus laevis* embryos, live imaging, single-cell tracking, optogenetics and loss-of-function experiments, we examine the roles of convergent extension and apical constriction, and define the role of the surface ectoderm during NTC. We show that NTC is a two-stage process with distinct spatiotemporal contributions of convergent extension and apical constriction at each stage. Convergent extension takes place during the first stage and is spatially restricted at the posterior tissue, whereas apical constriction occurs during the second stage throughout the neural plate. We also show that the surface ectoderm is mechanically coupled with the neural plate and its movement during NTC is driven by neural plate morphogenesis. Finally, we show that an increase in surface ectoderm resistive forces is detrimental for neural plate morphogenesis.

## Abstract (precis)

**Summary:** Detailed characterization of the contribution of distinct morphogenetic processes and mechanical tissue coupling during neural tube closure, a process indispensable for central nervous system formation in vertebrates.

## INTRODUCTION

The first crucial event during central nervous system development is the formation of the neural tube. The neural tube emerges from the dorsal ectoderm and is the precursor of the brain and spinal cord (Wallingford, 2005). Defective neural tube formation leads to neural tube defects (NTDs), which are some of the most common human birth defects, occurring in 0.5-2 per 1000 births (Greene and Copp, 2014). Thus, understanding the morphogenetic events driving neural tube formation will provide a better understanding of human NTDs and facilitate their prevention.

The transformation of the flat neuroepithelium into the neural tube is facilitated by intrinsic forces generated by morphogenetic events within the neural plate (NP). Studies in different vertebrate models have identified two distinct morphogenetic processes contributing to neural tube closure (NTC). Convergent extension (CE) (Davidson and Keller, 1999; Wallingford and Harland, 2002; Nishimura et al., 2012) and apical constriction (AC) (Hildebrand and Soriano, 1999; Haigo et al., 2003; Christodoulou and Skourides, 2015) are the two main morphogenetic movements taking place within the NP as it transforms into the neural tube.

CE is a morphogenetic process driven by polarized cell intercalation, leading to tissue lengthening and narrowing (Keller, 2002; Wallingford et al., 2002). Studies in mouse, frog and chicken embryos demonstrated that the planar cell polarity (PCP) pathway governs CE during NTC (Greene et al., 1998; Wallingford et al., 2002; Wallingford and Harland, 2002; Ybot-Gonzalez et al., 2007). Specifically, PCP controls the polarized activation of actomyosin (Nishimura et al., 2012; Shindo and Wallingford, 2014), which is essential for polarized junction remodelling and subsequent neighbour exchanges (Blankenship et al., 2006; Rauzi et al., 2008; Butler et al., 2009; Rauzi et al., 2010).

AC is a morphogenetic process driving the conversion of columnar cells to wedge-shaped cells as a result of apical surface area reduction (Martin and Goldstein, 2014). We and others have previously shown that during *Xenopus* NTC, AC is controlled by calcium transients and asynchronous medial actomyosin contraction events (Christodoulou and Skourides, 2015; Suzuki et al., 2017). AC is responsible for the generation of forces that lead to epithelial folding, driving the bending of the neuroepithelium in vertebrates (Lee et al., 1983; Sawyer et al., 2010; Inoue et al., 2016). Importantly, ablation of genes regulating AC results in NTDs (Hildebrand and Soriano, 1999; Haigo et al., 2003; Badouel et al., 2015; Grego-Bessa et al., 2015).

Interestingly, although defects in both CE and AC result in NTDs, failure of CE is correlated with spinal NTDs whereas failure of AC is correlated with anterior NTDs (Greene et al., 1998; Hildebrand and Soriano, 1999; Brouns et al., 2000; Wallingford et al., 2002; Haigo et al., 2003), suggesting that although these two morphogenetic processes are both indispensable for NTC they have discrete spatial and temporal contribution during neural tube morphogenesis. However, at present the precise spatiotemporal contribution of these processes towards NTC is not clear.

Several studies have suggested that, in addition to NP intrinsic morphogenesis, extrinsic forces generated by neighbouring tissues contribute to NP morphogenesis (Jacobson and Gordon, 1976; Moury and Schoenwolf, 1995; Wallingford et al., 2002). Specifically, it has been proposed that the surface ectoderm (SE) generates a pushing force that drives the medial movement of the neural folds. In support of this, a study suggested that active cell movement in the deep SE generates a pushing force during NTC (Morita et al., 2012). However, NP explants lacking the SE could still form a neural tube (Vijayraghavan and Davidson, 2017). Additionally, the tension within the SE is not anisotropic, which is contradictory with the notion that the SE generates a medially directed pushing force (Jacobson and Gordon, 1976), contributing to NTC.

Here, we aim to understand the spatiotemporal contribution of the morphogenetic processes within the NP and delineate the role of the SE in NTC. Using live imaging we show that NTC is biphasic and that the posterior and anterior regions of the NP display distinct behaviours. This is the result of differential PCP activity within the two regions. PCP-mediated cell intercalation at the posterior region drives CE of the tissue, which pushes the anterior region of the NP forward, positioning the anterior-most region in the middle of the dorsoventral axis. Moreover, we show that AC, unlike CE, is initiated throughout the NP during late NTC and is the major contributor of anterior NTC. We then show that the SE does not display active migratory behaviour during NTC, and its medial movement is passive and driven by NP morphogenesis. Finally, we show that a balance between the forces generated by NP morphogenesis and SE tension is required for NTC.

## RESULTS

### Macroscopic analysis of NTC reveals differential behaviour of the posterior and anterior domains

**Fig. 1.**
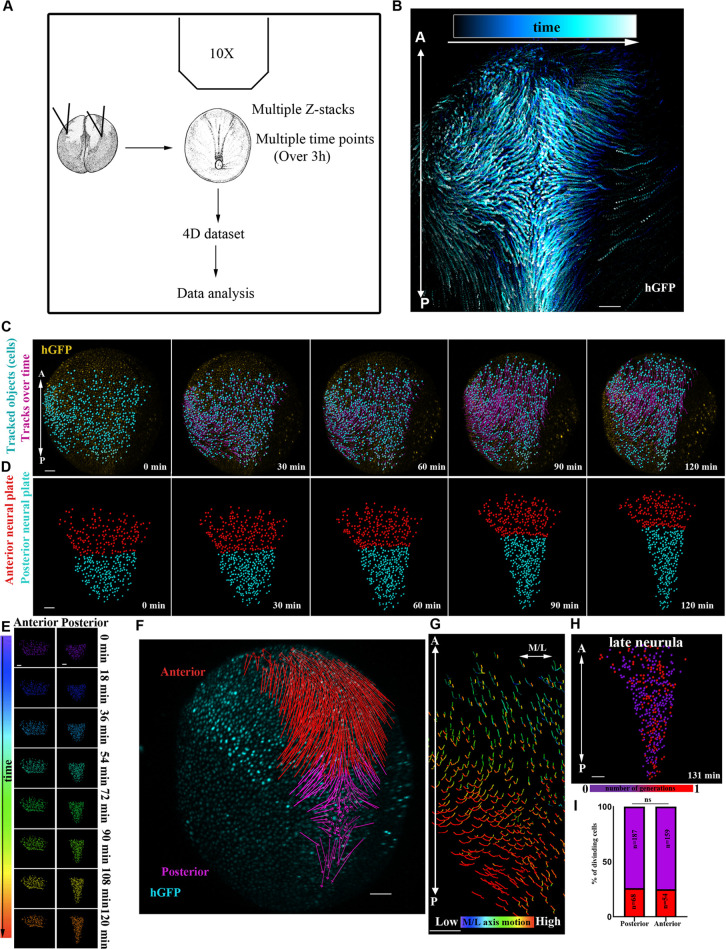
**Live imaging reveals differential behaviour of posterior and anterior NP regions.** (A) Schematic of the method used for the generation of 4D time-lapse recordings. (B) Representative temporal colour-coded maximum intensity projection of a time-lapse recording from an embryo expressing H2B-GFP (hGFP). (C) Stills from a single-cell-tracked time-lapse recording (Movie 1) of an embryo expressing H2B-GFP. (D) Stills from a tracked time-lapse recording revealing the behaviour of the posterior (spinal cord/hindbrain region) and anterior (midbrain/forebrain region) NP. The posterior NP narrows and elongates as time progresses, whereas the shape of the anterior NP remains the same. (E) Temporal colour-coded tracks shown in C displaying the evolution of anterior and posterior NP shape over time. (F) Displacement map of single-cell tracks overlaid over H2B-GFP signal at t0. The posterior NP cells move towards the midline. Anterior NP cells move anteriorly and ventrally. (G) ML motion direction colour-coded cell tracks revealing the absence of midline-directed movement from the anterior NP. (H) Generation colour-coded cell tracks. (I) Quantification of cell division at the posterior and anterior NP. χ^2^ test shows no significantly statistical differences (ns) with respect to cell division events at the posterior and anterior NP. A, anterior; P, posterior. Scale bars: 100 µm.

NTC is a process controlled by distinct morphogenetic events within the NP (Christodoulou and Skourides, 2015; Butler and Wallingford, 2018). However, our understanding of how these events are translated into collective tissue behaviour is poor. We thus decided to generate single-cell-resolution maps of the collective tissue movements taking place during NTC. To accomplish this, we acquired high temporal resolution time-lapse recordings of the entire NP of *Xenopus* embryos during NTC (Fig. 1A,B). Histone-GFP mRNA was microinjected at the animal pole of both blastomeres of two-cell-stage embryos. The embryos were allowed to develop to stage 12.5-13 and then imaged. We generated time-lapse recordings by collecting 50-60 *z*-stack images at 8 µm intervals every 3 min encompassing the entire dorsal half of the embryo. After the generation of time-lapse recordings, we tracked single cells (468 neuroepithelial and 348 SE cells) (Fig. 1B,C, Movie 1). This revealed that neuroepithelial cells display district motion patterns along the anteroposterior (A/P) axis of the embryo. These results are in agreement with classical studies following neuroepithelial cells displacement using fluorescein-Dextran labelling (Keller et al., 1992). Specifically, anterior NP cells display a linear movement towards the embryo's anterior ventral side, whereas the posterior NP cells move towards the midline (Fig. 1C-G, Movie 1). Additionally, we observed distinct tissue-wide behaviour along the A/P axis. The posterior NP (spinal cord-hindbrain region) lengthens and narrows, whereas the shape of the anterior NP (midbrain-forebrain region) retains its geometry but translocates and is repositioned at a more anterior and ventral area of the embryo (Fig. 1C-E, Movie 1). This was confirmed by tracking the posterior and anterior NP deformation over time, using targeted photoconversion of mEos (Wiedenmann et al., 2004) (Fig. S1A-C, Movie 2). We went on to examine the possibility that differences in cell proliferation rates or cell division orientation contribute to the different movement patterns of the NP along the A/P axis. Cell division orientation relative to the A/P axis was the same at the posterior (mean cell division angle 48±3°, 250 cells, five embryos) and anterior (mean cell division angle 49±2°, 250 cells, five embryos) NP. At the same time, cell proliferation rates were uniform throughout the NP (Fig. 1H,I, Movie 3). Lengthening and narrowing of tissues are driven by PCP-mediated intercalative behaviour during CE (Nishimura et al., 2012; Shindo and Wallingford, 2014; Huebner and Wallingford, 2018). This raises the possibility that the anterior and posterior regions of the NP possess different intrinsic patterns of morphogenesis owing to restricted PCP-mediated intercalative behaviour at the posterior of the tissue.

### PCP-mediated cell intercalative behaviour is restricted to the posterior NP

**Fig. 2.**
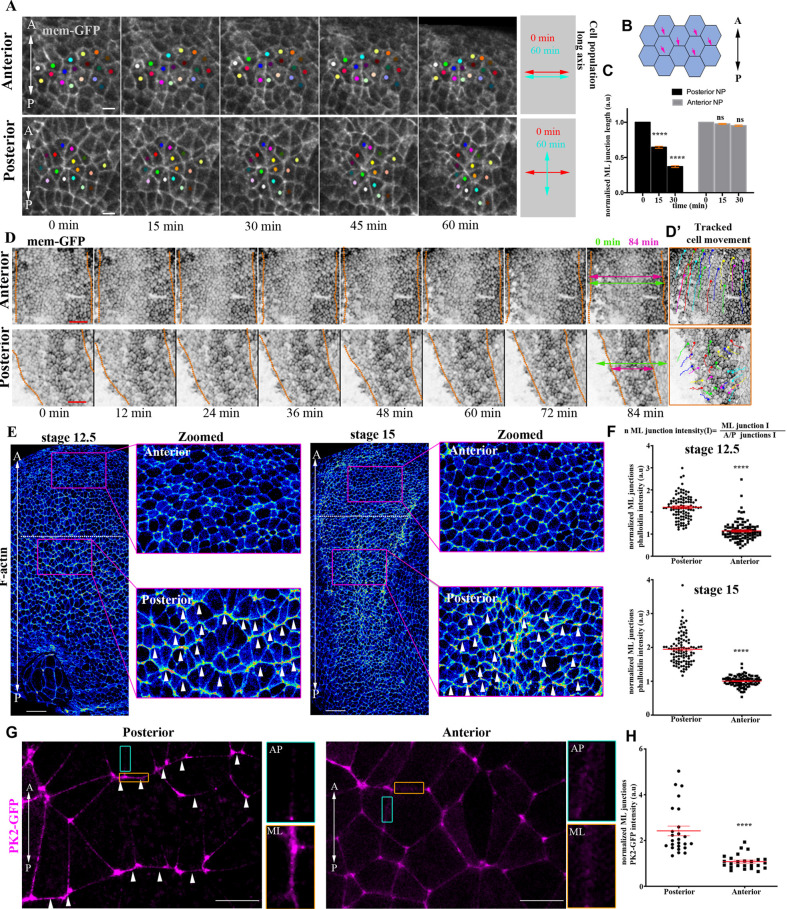
**PCP-mediated cell intercalative behaviour is restricted to the posterior NP.** (A) Stills from a time-lapse recording of a neurula-stage embryo. Coloured spots represent single neuroepithelial cells tracked over time. At the posterior region, cells display polarized intercalative behaviour leading to neighbour exchanges. Scale bars: 20 µm. (B) Schematic showing the neuroepithelial cell-cell junctions (pink arrows) used for the quantification of ML junction length in C. (C) Quantification of relative ML junction length at different time points. ****P<0.0001 (two-way ANOVA); mean±s.e.m.; *n*=4 embryos, 210 anterior and 210 posterior junctions. (D) Stills from a time-lapse recording of a neurula-stage embryo. Dotted lines indicate NP/SE boundaries. NP width (double-headed arrows) is reduced only at the posterior NP region. Scale bars: 100 µm. (D′) Tracked movement of neuroepithelial cells at the posterior and anterior NP. (E) Signal intensity colour-coded maximum intensity projections of representative neurula-stage embryos stained with Phalloidin (F-actin). Dotted line represents the A/P NP boundary. Arrowheads in zoomed images highlight the enrichment of F-actin at ML junctions. *n*=10 embryos for each stage. Scale bars: 100 μm. (F) Quantification of F-actin fluorescence intensity at ML junctions. ****P<0.0001 (two-sided, unpaired Student's *t*-test); mean±s.e.m.; *n*=100 junctions. (G) Representative images from a stage 14 neurula embryo exogenously expressing Prickle2-GFP (PK2-GFP). Arrowheads indicate PK2-GFP enrichment at ML junctions within the posterior NP. A/P (green box) and ML (orange box) junctions are shown at higher magnification in the images to the right. *n*=5 embryos. Scale bars: 20 µm. (H) Quantification of PK2-GFP localization at ML junctions. ****P<0.0001 (two-sided, unpaired Student's *t*-test); ns, not significant; mean±s.e.m.; *n*=100 junctions. A, anterior; a.u., arbitrary units; P, posterior.

Polarized junction shrinkage and basal protrusive activity are both essential for PCP-mediated cell intercalation during vertebrate NTC (Williams et al., 2014). To examine intercalative activity across the NP, we tracked single neuroepithelial cells over time. This revealed that cells at the posterior NP display neighbour exchange events (Rauzi et al., 2008). These neighbour exchange events resulted in the re-orientation of the long axis of cell collectives from perpendicular to parallel with respect to the A/P axis (Fig. 2A, Movie 4). In contrast, anterior NP cells did not display neighbour exchange events (Fig. 2A, Movie 4). In agreement with the above, polarized junction shrinkage, which takes place during polarized intercalative behaviour, was evident only in the posterior part of the tissue (Fig. 2B,C). This polarized cell intercalative behaviour underlies the CE-driven narrowing and lengthening of the posterior NP. At the same time, the anterior part of the tissue did not change dimensions and simply moved to a more anterior position (Fig. 2A,D, Fig. S1A-C, Movies 2, 5). Overall, these data reveal that CE movements are restricted to the posterior part of the NP.

Junction remodelling, controlled by PCP-mediated actomyosin contractility, drives polarized cell intercalative behaviour during NTC (Nishimura et al., 2012; Butler and Wallingford, 2018). If the differential behaviour of the anterior and posterior NP is underpinned by differential PCP-driven intercalative behaviour, then the actomyosin network would be expected to display pattern variations along the A/P axis of the neuroepithelium. To investigate this possibility, we stained early and mid-neurula embryos with Phalloidin to examine F-actin localization. We observed that F-actin displayed enriched localization at mediolateral (ML) neuroepithelial cell-cell junctions only in the posterior part of the tissue, whereas it displayed uniform localization at the anterior NP (Fig. 2E,F). Given that actomyosin localization at ML junctions is controlled by PCP signalling, we decided to examine PCP polarity across the NP. The establishment of PCP typically involves a global orienting cue and the asymmetric segregation of dedicated polarity proteins. Prickle2 (PK2) is a PCP member that has previously been shown to mark PCP-mediated junction remodelling events within the NP (Butler and Wallingford, 2018). We thus used exogenously expressed PK2-GFP to examine PCP polarity within the NP. As shown, PK2 displayed enriched localization at ML cell-cell junctions at posterior regions of the NP, but a uniform distribution in cells of the anterior (Fig. 2G,H). In summary, these data clearly show that anterior NP lacks PCP polarization and does not undergo CE movements.

### NP anterior movement requires CE of the posterior NP region

**Fig. 3.**
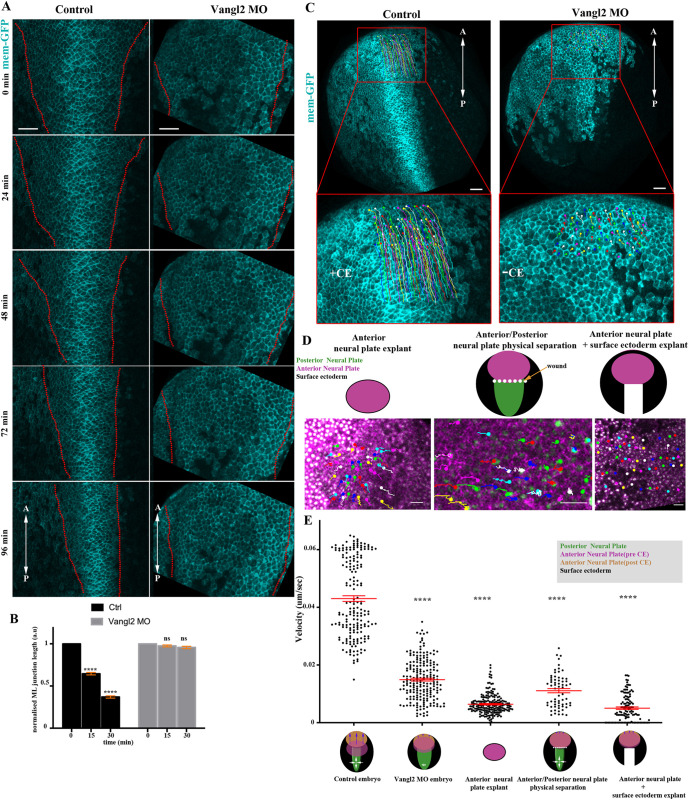
**NP anterior movement requires CE of the posterior tissue.** (A) Stills from time-lapse recordings of representative control and Vangl2 morphant embryos. Dotted lines delineate NP boundaries. NP narrowing and neural fold movement towards the midline is defective in the Vangl2 morphant embryo. (B) Quantification of ML junction length over time in control (*n*=4) and Vangl2 morphant (*n*=3) embryos. ****P<0.0001 (two-sided, unpaired Student's *t*-test); ns, not significant; mean±s.e.m.; *n*=210 and 148 junctions from control and Vangl2 morphant embryos, respectively. (C) Representative examples of anterior neuroepithelial cell displacement within a 60 min time period in a control and Vangl2 morphant embryo. Boxed areas are shown at higher magnification below. (D) Representative examples of anterior neuroepithelial cell displacement within a 60 min time period in an anterior NP explant, in an embryo with wound-induced physical separation of anterior and posterior NP and in embryos lacking the posterior NP. (E) Quantification of anterior neuroepithelial cell velocity. ****P<0.0001 (one-way ANOVA); mean±s.e.m.; *n*=187 cells from three control embryos 209 cells from three Vangl2 morphant embryos; 202 cells from three anterior NP explants; 64 cells from an embryo with wound-induced physical separation of anterior and posterior NP; and 120 cells from three embryos lacking the posterior NP. Scale bars: 100 µm. A, anterior; a.u., arbitrary units; P, posterior.

The movement of the anterior NP towards the rostral region takes place concomitantly with the remodelling of the posterior NP due to CE. The anterior NP movement might be either active or passive in response to pushing force caused by elongation of the posterior NP. To test these possibilities, we decided to inhibit CE, which takes place only at the posterior part of the tissue, and then examine the movement of the anterior NP. We blocked CE by downregulating the PCP member Vangl2, the vertebrate homologue of Vang (Devenport, 2014), by targeted microinjections of a previously characterized morpholino against Vangl2 (Darken et al., 2002; Ossipova et al., 2015) (Fig. S2A-C). Comparison of NTC in control and Vangl2 morphant embryos revealed that ML junction shrinkage, a hallmark of polarized cell intercalative behaviour controlled by Vangl2 (Williams et al., 2014), and the medial movement of the neural folds are impaired upon downregulation of Vangl2 within the NP (Fig. 3A,B, Movie 6). Altogether, these results confirm that CE is impaired in Vangl2 morphants.

We went on to examine how the movement of the anterior NP is affected in Vangl2 morphant embryos. The movement of the anterior NP towards the future rostral site was blocked in Vangl2 morphants, suggesting that its repositioning during NTC is passive (Fig. 3C,E, Fig. S3A-C, Movie 7). Another interpretation of these results could be that anterior movement is active; however, posterior CE is permissive for anterior translocation because the two are inherently linked. To examine this possibility, we assessed anterior NP movement in anterior NP explants, anterior NP/epidermis explants and in embryos in which the anterior and posterior regions of the NP were physically decoupled by a wound (Fig. 3D). Anterior NP movement was defective in all cases (Fig. 3D,E, Fig. S4A-C, Movies 8-10), revealing that physical coupling between the anterior and posterior NP is necessary for passive movement of the anterior tissue. In support of the latter, in Vangl2 morphants the A/P length of posterior NP cells proximal to the anterior tissue did not change over time, showing that these cells do not experience pulling forces generated by the anterior-most tissue (Fig. S4D). Overall, our data show that CE extension has a dual role during NTC. At the posterior part of the NP, CE drives the active narrowing and lengthening of the tissue that leads to passive rostral positioning of the anterior tissue.

### Spatiotemporal contribution of AP during NTC

**Fig. 4.**
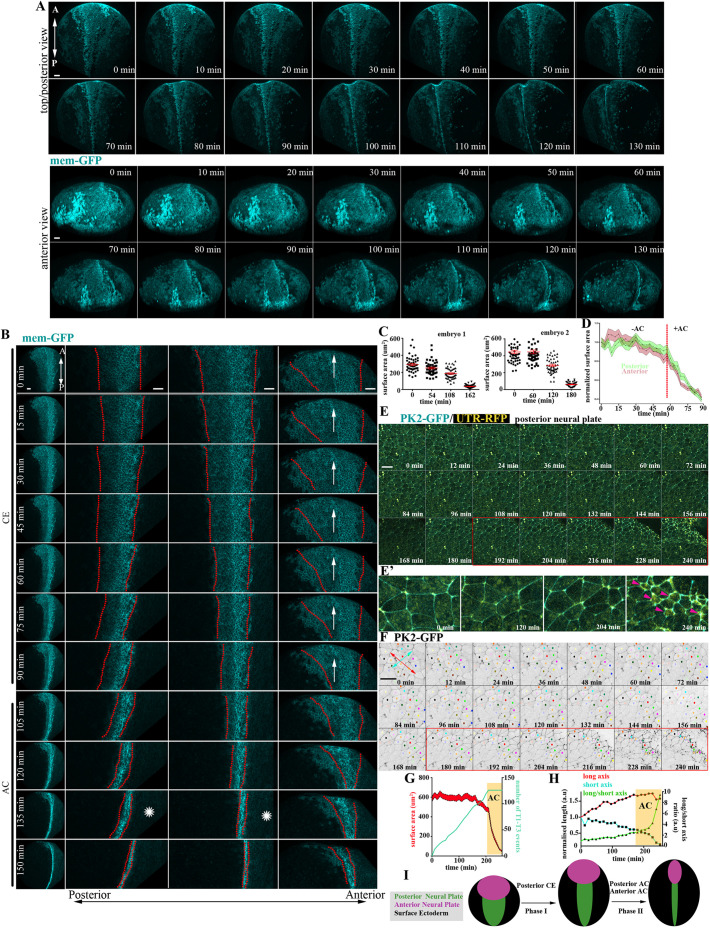
**AC is initiated subsequent to CE and is the major contributor of NTC the anterior region.** (A) Stills from a 3D-rendered, time-lapse recording of a neurula-stage embryo. Scale bars: 100 µm. (B) Stills from a time-lapse recording of a second neurula-stage embryo. In the left column, the entire embryo is shown. The next three columns show zoomed NP areas (posterior to anterior, from left to right). CE at the posterior NP precedes AC and is concomitant with the forward displacement (arrow) of the anterior region. Posterior NTC is completed before anterior NTC (asterisks). Scale bars: 100 µm. (C) Quantification of neuroepithelial cell apical cell-surface area of two different embryos. Data are mean±s.e.m.; *n*=50 cells for all time points except the last time point of embryo 2, which is 35 cells. (D) Quantification of apical cell-surface area of posterior and anterior NP cells shows that AC (rapid reduction of apical surface area) occurs simultaneously at the posterior and anterior NP. *n*=30 posterior and 30 anterior NP cells. (E) Stills from a time-lapse recording from the posterior region of a neurula-stage embryo. Imaging was initiated at stage 12.5. Red box indicates the period during which AC takes place. Scale bar: 50 µm. (E′) Zoomed images of selected time points from E. Arrowheads indicate the appearance of apical-medial actomyosin during AC. (F) Cell tracking of magnified regions of the images shown in E. Neighbour exchanges (red and yellow cells are an example) only take place before AC initiation (red box). Double-headed arrows were used for the quantification shown in H: red indicates the length of the cell collective at the A/P direction, cyan indicates the length of the cell collective at the ML direction. Scale bar: 50 µm. (G) Quantification of apical cell surface area and T1-T3 transitions over time. Initiation of AC is marked by the yellow box. The coloured area in the graph for the cell surface area represents s.e.m.; *n*=30 cells for each time point. (H) Quantification of A/P length of cell collective (long axis; red line), ML length of cell collective (short axis; cyan) and the ratio of A/P and ML (long/short axis; green line). A/P length increases through CE until the initiation of AC when it is slightly reduced, indicating the absence of cell intercalative behaviour. The A/P length is dramatically reduced during AC, when the tissue begins to bend towards the midline. (I) Schematic showing the contribution of AC and CE to changes in morphology during NTC. A, anterior; a.u., arbitrary units; P, posterior.

Efficient NTC is realized by the combined activities of CE and AC of neuroepithelial cells (Wallingford and Harland, 2002; Haigo et al., 2003; Ybot-Gonzalez et al., 2007; Christodoulou and Skourides, 2015; Balashova et al., 2017; Marivin and Garcia-Marcos, 2019). Thus, we went on to investigate the spatiotemporal contribution of AC during NTC. We generated time-lapse recordings of embryos expressing membrane fluorescent markers. This revealed that AC is initiated during late NTC stages (Fig. 4A-C, Fig. S5A, Movies 11-13) and, unlike CE, AC is initiated synchronously across the entire NP (Fig. 4A,B,D, Fig. S5A,B). To examine whether AC and CE temporarily overlap during NTC, we generated high-resolution, time-lapse recordings of the posterior neuroepithelium. This revealed that polarized neighbour exchanges, which drive CE, cease when AC initiates (Fig. 4E-H, Movies 14, 15). In agreement with our data, it was recently shown that optogenetic induction of AC results in inhibition of intercalative behaviour during *Drosophila* development (Herrera-Perez et al., 2021). To confirm that AC does not contribute to the first phase of NTC, we downregulated Shroom3, a known regulator of AC (Haigo et al., 2003). Analysis of time-lapse recordings of Shroom3 morphant embryos shows that CE was unaffected, as indicated by the midline-directed movement of the neural folds and the NTC rate during the first phase of NTC (Fig. S6A,B, Movie 16). In contrast, the second phase of NTC, which is driven by AC, was defective, as evident by quantification of the NTC rate and NP cell surface area over time (Fig. S6B,C). In agreement with our data, Crispr/Cas-mediated knock out of Shroom3 in *Xenopus* embryos leads to a mild effect on the polarity of T1 transitions, but does not affect the number of T1 transitions and CE-mediated tissue deformation (Baldwin et al., 2022). Altogether, our data show that NTC is clearly biphasic with an early CE-driven phase that is spatially restricted to the posterior NP, followed by a second phase driven by generalized AC across the entire NP (Fig. 4I).

AC has been linked with anterior NTDs (Hildebrand and Soriano, 1999; Grego-Bessa et al., 2015). For example, inhibition of cell-autonomous calcium flashes (using the voltage-gated calcium channel inhibitor nifedipine), which regulate AC during *Xenopus* NTC (Christodoulou and Skourides, 2015), results only in defective anterior NTC (Fig. S6D). Given that during the second phase of NTC AC is taking place across the entire NP, we hypothesized that CE could compensate for posterior NTDs in embryos with defective AC. To examine this possibility, we compared time-lapse sequences from Shroom3 morphants, in which AC is absent, and control embryos. Our analysis revealed that polarized intercalative cell behaviour within the posterior NP of Shroom3 morphants is prolonged and continues well beyond that of control embryos (Fig. S6E-G, Movie 17). This shows that CE is temporally extended in these embryos, compensating for the loss of AC and moderating its effects on posterior NTC.

### NP morphogenesis is necessary for SE medial movement

**Fig. 5.**
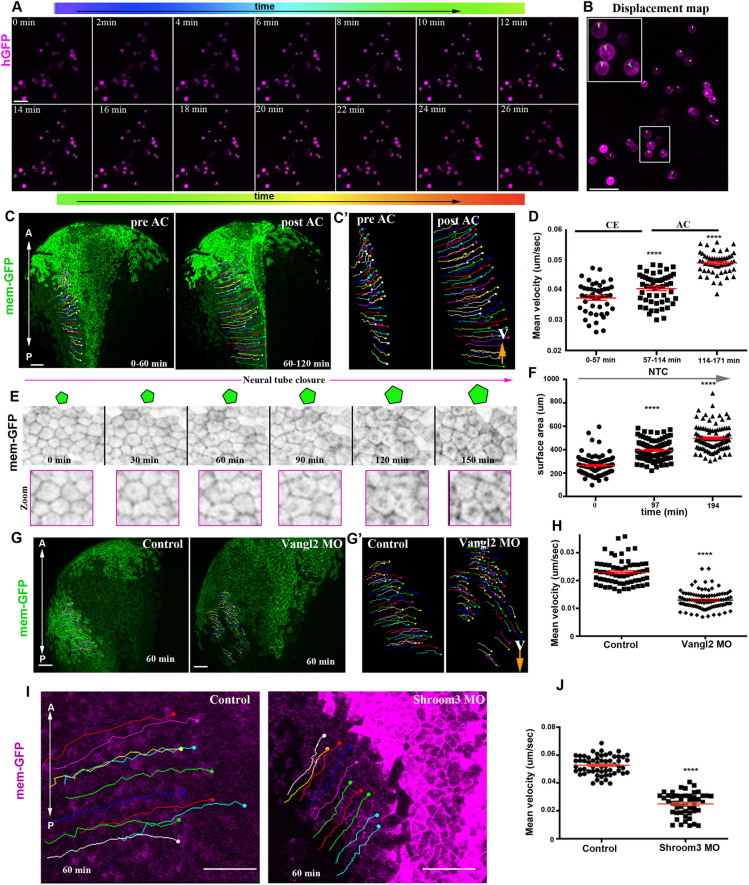
**SE medial movement requires forces generated by NP morphogenesis.** (A) Stills from a time-lapse recording of deep SE cells plated on an FN-coated coverslip. Tracks (spots) are time colour-coded. (B) Displacement map from a 26 min time window from the time-lapse recording used for cell tracking in A, indicating absence of cell movement. Boxed area is shown at higher magnification in inset. (C) Stills from a tracked time-lapse recording of a control embryo before (left) and after (right) AC. (C′) Zoomed tracks from the recording shown C showing the increase of cell displacement and velocity (V) during AC. (D) Quantification of the average cell velocity of SE cells during distinct phases of NTC. ****P<0.0001 (two-sided, unpaired Student's *t*-test); mean±s.e.m. *n*=46, 53 and 54 cells for the three different time periods. (E) Stills from a tracked time-lapse recording showing SE cells during NTC. As NTC progresses, the surface area of SE cells increases, as shown by the schematics above. (F) Quantification of the apical surface area of SE cells during NTC. ****P<0.0001 (two-sided, unpaired Student's *t*-test); mean±s.e.m. *n*=100 cells for each time point. (G) Stills from a tracked time-lapse recording of a control embryo (left) and a Vangl2 morphant embryo (right). (G′) Zoomed tracks from the recording shown in C showing decrease of cell displacement and velocity (V) in the absence of CE in Vangl2 morphants. (H) Quantification of the average cell velocity from control and Vangl2 morphant embryos. ****P<0.0001 (two-sided, unpaired Student's *t*-test); mean±s.e.m., *n*=63 SE cells from a control and 97 SE cells from a Vangl2 morphant embryo. (I) Stills from a tracked time-lapse recording of a control embryo (left) and a Shroom3 morphant embryo (right). The displacement and velocity (V) of SE cells is decreased in the absence of AC. (J) Quantification of the average cell velocity from control and Shroom3 morphant embryos. ****P<0.0001 (two-sided, unpaired Student's *t*-test); mean±s.e.m., *n*=59 SE cells from a control and 49 SE cells from a Shroom3 morphant embryo. Scale bars: 100 µm. A, anterior; P, posterior.

In addition to active force-generating morphogenetic events taking place within the NP, it has been proposed that extrinsic mechanical forces also contribute actively to NTC. Specifically, it has been suggested that active migration of deep SE cells is necessary for NTC in *Xenopus* embryos (Morita et al., 2012). However, this is in contrast with studies showing that excised NP explants can elongate and roll up (Vijayraghavan and Davidson, 2017). Furthermore, the argument supporting an active movement of deep epidermal cells is inconsistent with the fact that that deep ectoderm cells from gastrula-stage embryos, unlike mesodermal cells, do not have the capacity to migrate when explanted from the embryo (Kashkooli et al., 2021). In order to examine whether deep SE cells become migratory during neurulation and differ from their gastrula-stage counterparts, we explanted deep SE cells from stage 14 allowed them to attach to fibronectin (FN)-coated substrates and subsequently generated time-lapse recordings. This revealed that deep SE cells cannot migrate when cultured *ex vivo* on FN substrates as other migratory cell types do, such as the head mesoderm during gastrulation and neural crest during neurulation (Winklbauer, 1990; Alfandari et al., 2010) (Fig. 5A,B, Movie 18). To examine the migratory capacity of the SE in the embryo, we tracked deep and superficial SE cells. This revealed that deep cells move together with the superficial epithelial cells and never overtake superficial cells (Fig. S7A, Movie 19), indicating that the deep SE cells do not move autonomously, but rather their movement is coupled with the movement of the superficial epithelial layer.

Analysis of tissue tensile properties in neurula axolotl embryos has revealed that tissue tension within the NP is higher than that of the SE (Wiebe and Brodland, 2005). Thus, the SE as a softer tissue would not be expected to generate forces able to deform the stiffer neuroepithelium. Therefore, we formed the hypothesis that the movement of the SE towards the midline is passive and a consequence of pulling forces generated by neural tube morphogenesis. To test this hypothesis, we tracked SE cells during NTC in control embryos. This revealed that the velocity of SE cells increases slightly during CE and shows a dramatic increase when neuroepithelial cells undergo AC (Fig. 5C-D, Movie 20), the NTC phase with the higher rate of neural fold displacement (Christodoulou and Skourides, 2015). Moreover, single-cell tracking analysis shows that the lateral SE epithelium movement mirrors the movement of the NP (Fig. S7B). Importantly, quantification of SE cell surface area during NTC revealed that the surface area of superficial ectoderm cells adjacent to the NP increases as NTC progresses (Fig. 5E,F). This indicates that the SE cells are stretched as a result of active ML movement of the NP.

The anterior SE, which is found beneath the forebrain region, displays a unique behaviour during NTC. Specifically, this region of the SE displays an anterior and ventral directed movement during the first phase of NTC following the movement of the anterior NP, which passively moves anteriorly and ventrally (Fig. S7C, Movie 21). During the second phase of NTC, the cells in this region of the SE change behaviour and are stretched along the dorsoventral axis by the folding anterior NP and acquire an elongated shape (Fig. S7D,E, Movies 22,23). These cell shape changes are characteristic of tissues experiencing tensile forces, generated by the active remodelling of a neighbouring tissue (Butler et al., 2009; Wyatt et al., 2015). All the above findings indicate that the SE moves passively towards the midline during neurulation and its movement is coupled with NP morphogenesis.

To assess directly the impact of NP morphogenesis on SE movements, we decided to inhibit CE and AC within the NP and then examine the movement of the SE. The movement of the SE towards the midline was impaired in Vangl2 morphant embryos (Fig. 5G-H, Movie 24), showing that NP CE contributes to the movement of the SE. Similarly, the movement of the SE was impaired in Shroom3 morphants, in which AC is defective (Fig. 5I,J Movie 25). Overall, these data show that NP morphogenesis generates forces that contribute to the expansion and movement of the SE towards the midline.

### NP-restricted force generation is sufficient to drive the medial movement of the SE

**Fig. 6.**
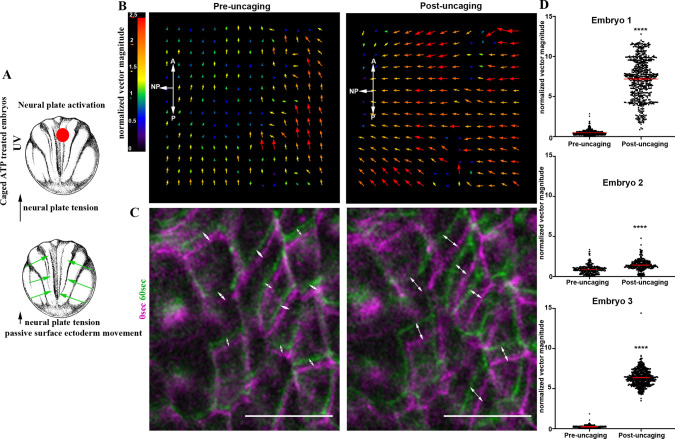
**Force generation within the NP is sufficient to drive movement of the SE.** (A) Schematic showing the experimental approach to increase tissue tension within the NP by optogenetic uncaging of ATP. (B) PIV analysis illustrates the increased SE movement upon ATP uncaging within the NP. (C) Time colour-coded representative images of the SE before and after NP-targeted ATP uncaging. Double-headed arrows indicate cell displacement. Scale bars: 50 μm. (D) Quantification of normalized SE movement vector magnitude before and after NP-targeted ATP uncaging. ****P<0.0001 (two-sided unpaired Student's *t*-test); mean±s.e.m., *n*=326 vector from embryo 1; 595 vectors for embryo 2; and 601 vectors for embryo 3. A, anterior; P, posterior.

To assess directly the mechanical interplay between the SE and the NP, we decided to use optogenetics to modulate contractility in a spatially and temporally controlled manner. Specifically, we used caged ATP because ATP release has been shown to be sufficient to induce cell contractility in the *Xenopus* ectoderm epithelium (Kim et al., 2014). Targeted uncaging of ATP will lead to an increase in cell contractility and tissue tension at the selected tissue region. Targeted NP ATP uncaging (Fig. 6A) resulted in a rapid change of SE movement pace towards the excited NP region (Fig. 6B-D, Fig. S8A,B, Movie 26). These data show that cells of the SE respond directly to forces generated within the NP and that forces generated within the NP are sufficient to move the SE medially. Furthermore, our data highlight the close mechanical linkage between the NP and the SE and strongly support the notion that SE movements during neurulation are passive.

### Normal SE development is necessary for NTC

**Fig. 7.**
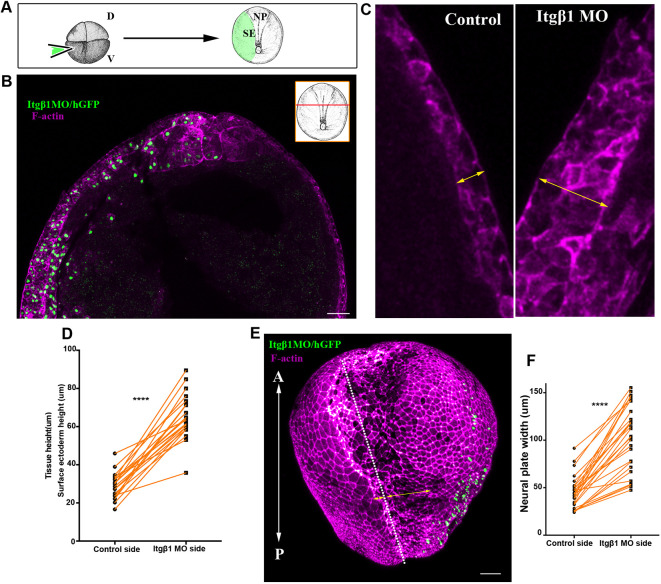
**Normal SE development is necessary for NTC.** (A) Schematic showing the process for SE targeted microinjections. D, dorsal; V, ventral. (B) Representative example from a cross-section (inset) of a stage 15 embryo with SE-targeted unilateral injection of Itgβ1 MO. Itgβ1 MO was co-injected with histone-GFP (hGFP). *n*=21 embryos. (C) High-magnification images of the SE shown in B. The thickness of the SE (double-headed arrows) is increased on the Itgβ1 morphant side. (D) Quantification of SE thickness in embryos unilaterally injected with Itgβ1 MO. ****P<0.0001 (two-sided, paired Student's *t*-test); mean±s.e.m., *n*=21 embryos. (E) 3D image of a representative example of a stage 16 embryo unilaterally injected with Itgβ1 MO. White dotted line indicates the midline. Double-headed arrows indicate the length of the NP in the control and Itgβ1 MO-injected side. *n*=18 embryos (F) Quantification of NP length at the control side and the Itgβ1 MO side in embryos unilaterally injected with Itgβ1 MO. ****P<0.0001 (two-sided, paired Student's *t*-test); mean±s.e.m., *n*=28 embryos. A, anterior; P, posterior. Scale bars: 100 μm.

We postulated that neural tube morphogenesis would require a balance between the active forces generated by NP morphogenesis and SE tension. Specifically, SE tension should be sufficiently low to allow tissue deformation and expansion in order to permit NTC. Integrin-β1 (Itgβ1) signalling has been shown to control cell division orientation in the ectoderm of gastrula-stage embryos and absence of Itgβ1 signalling leads to tissue thickening (Marsden and DeSimone, 2001). Importantly, tissue thickening has been shown to lead to increased tissue tension (Petridou et al., 2013). To test the effects of increased SE tissue tension on NTC, we injected a previously characterized Itgβ1 morpholino (Morita et al., 2012) into one ventral blastomere at the four-cell stage to obtain embryos with unilateral SE Itgβ1 downregulation (Fig. 7A, Figs S9A-C, S10A,B). Itgβ1 downregulation in the SE led to thickening of the tissue, as expected (Fig. 7B-D, Fig. S10C), and impaired NTC (Fig. 7E,F, Fig. S10D,E, Movie 27). This phenotype was previously attributed to the loss of active deep SE cell migration (Morita et al., 2012). Although this interpretation cannot be ruled out, it is highly unlikely to be correct based on our data indicating that SE movements are passive.

**Fig. 8.**
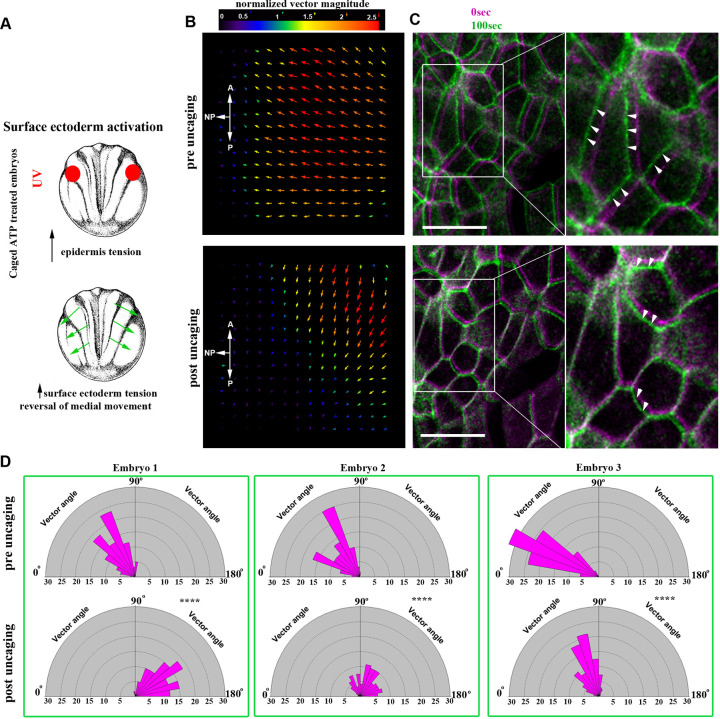
**Increased SE contractility negatively impacts NTC.** (A) Schematic showing the experimental approach to increase tissue tension within the SE through optogenetic uncaging of ATP. (B) PIV analysis illustrating reversed movement of SE upon ATP uncaging within the SE. A, anterior; P, posterior. (C) Time colour-coded representative images of the SE before and after SE-targeted ATP uncaging. Boxed areas are shown at higher magnification to the right. Arrowheads indicate the direction of cell displacement. Scale bars: 50 μm. (D) Quantification of SE movement vector angle presented as a rose diagram showing the percentage of angles values between 0 and 180°. 0° represents movement parallel with the ML embryo axis and 90° represents movement parallel with the embryo A/P axis. ****P<0.0001 (Kolmogorov–Smirnov test). *n*=60 vector angles for embryo 1; 120 vector angles for embryo 2; and 400 vector angles for embryo 3. A, anterior; P, posterior.

According to our hypothesis, thickening of the SE in Itgβ1 morphant embryos would lead to an increase of tissue tension within the SE and increased resistance to the forces generated within the NP, leading to defective NTC. To test this hypothesis directly, we employed optogenetic induction of cell contractility. Specifically, we allowed embryos treated with caged-ATP to develop to neurula stages and subsequently uncaged ATP within the SE to increase tension in this tissue (Fig. 8A). We observed that upon increase of SE contractility NTC velocity slowed down and the direction of SE cell movement was reversed (Fig. 8B-D, Fig. S11A,B, Movie 28). These data suggest that defects in Itgβ1 SE morphants stem from elevated resistive forces rather than loss of deep cell migration. In summary, our data show that although the SE expands passively during NTC, its proper development and maintenance of correct tissue tension levels are permissive for NTC.

**Fig. 9.**
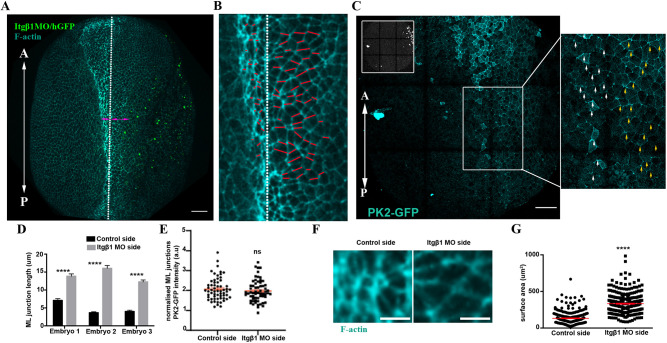
**AC and CE are defective in embryos with defective SE development.** (A) Representative example of an embryo unilaterally injected with Itgβ1 MO. White dotted line indicates the midline. Double-headed arrows indicate the length of the NP in the control and Itgβ1 MO-injected side. *n*=20 embryos. (B) High magnification image of the NP from A. White dotted line demarcates the midline. Red lines mark the ML junctions. ML junctions at the NP side adjacent with Itgβ1 morphant SE fail to shrink. (C) Representative example of an embryo expressing PK2-GFP and unilaterally injected with Itgβ1 MO (inset shows H2B-GFP). Boxed area is shown at higher magnification to the right. PK2 displays enriched localization at ML junctions of the control NP side (white arrows) and the NP side adjacent with Itgβ1 morphant SE (yellow arrows). (D) Quantification of ML junction length in the control NP side and the adjacent NP side with Itgβ1 morphant SE. ****P<0.0001 (two-sided, unpaired Student's *t*-test); mean±s.e.m., *n*=50 junctions. (E) Quantification of ML PK2-GFP localization in control NP side and the adjacent NP side with Itgβ1 morphant SE. ns, not significant (two-sided, unpaired Student's *t*-test); mean±s.e.m., *n*=60 junctions. (F) Representative neuroepithelial cell surface area in control NP and the adjacent NP with Itgβ1 morphant SE. (G) Quantification of apical cell surface area of neuroepithelial cells. ****P<0.0001 (two-sided, unpaired Student's *t*-test); mean±s.e.m., *n*=300 cells from six control embryos and 300 from six Itgβ1 morphant embryos. Scale bars: 100 µm. A, anterior; P, posterior.

Given the NTC defects observed upon elevated tension within the SE, we then investigated which NP morphogenetic events are affected under elevated resistive forces from the SE. To address specifically how these processes are affected by elevated tension, we turned to Itgβ1 SE morphant embryos, in which elevated tension is maintained throughout NTC. To assess CE, we analysed the length of ML junctions. We found that ML junctions fail to shrink at the NP side adjacent to the Itgβ1 morphant SE (Fig. 9A,B,D), even though planar polarization of the tissue was preserved (Fig. 9C,E). To assess the effects on AC, we quantified the apical cell surface area of neuroepithelial cells. This revealed that neuroepithelial cells at the side adjacent to the morphant SE had a significantly larger average apical surface area (Fig. 9F,G), indicative of defective AC. This suggests that the resistive forces from the stiffened SE cannot be overcome by the force-generating machinery of the NP, leading to defects in both AC and CE. Overall, our data show that, although the SE does not actively contribute to neural tube morphogenesis, its mechanical linkage to the NP imposes opposing forces that must be carefully regulated to allow NTC. We conclude that SE morphogenesis is permissive for NTC.

## DISCUSSION

In this study, we describe the spatiotemporal contribution of the morphogenetic events taking place within the NP and we delineate the role of the SE during NTC. Our findings demonstrate that NTC is driven by a precisely orchestrated spatiotemporal pattern of morphogenetic events. Additionally, we show that NP is mechanical coupled with the SE, the expansion of which is permissive for NTC.

High-resolution imaging of NP morphogenesis revealed that the anterior and posterior regions of the NP display distinct morphogenetic behaviour. This is an agreement with a recent study that also detected distinct patterns of behaviour at the posterior and anterior NP (Baldwin et al., 2022). Specifically, here we show that whereas the posterior NP narrows and elongates, the anterior part of the tissue displays a polarized anterior movement. Importantly, our data indicate that the anterior movement of the anterior NP is passive, and its correct positioning depends on the polarized intercalative behaviour of the posterior NP. The possibility that anterior NP cells move actively during neurulation was excluded by the fact that in Vangl2 morphants we do not observe stretching of posterior neuroepithelial cells as a result of tensile forces transmitted from the movement of the anterior part of the tissue.

Our results also reveal that the distinct behaviour of the anterior and posterior NP depends on PCP-mediated intercalative cell behaviour being present only at the posterior part of the tissue. Recently, it was shown that the blastopore lip found at the posterior end of the NP is a source of PCP-inducing signals (Mancini et al., 2021). Thus, it is likely that these signals originating from the posterior-most area of the NP are responsible for the differential behaviour of the posterior and anterior NP.

When we analysed their spatiotemporal contribution of CE and AC during NTC, we found that CE contributes only to the posterior NP during the first phase of NTC. During this phase, the anterior NP moves towards the dorsoventral midline without any deformation. Subsequently, during the second phase AC takes place at both the posterior and anterior of the tissue with no temporal overlap with CE. Our data show that in the absence of AC, CE takes place and is prolonged at the spinal NP, and this is likely responsible for mitigating NTDs at the posterior NP when AC is defective.

The contribution of extrinsic forces, generated by neighbouring tissues, to NTC has been controversial over the years. Here, we focused on the role of the SE during NTC. Our results indicate that the deep SE cells do not have a migratory capacity, in contrast with a previous study (Morita et al., 2012). These results agree both with the fact that deep ectoderm cells from gastrula embryos are not migratory (Kashkooli et al., 2021), and with the fact that SE explants developed up to tailbud stages never display any migratory behaviour. Using live imaging we show that the SE movement mirrors the movement of the NP, and its movement is directly influenced by NP morphogenesis. We also show, using optogenetics, that induction of contractility within the NP is sufficient to elicit directed movement of the SE towards the NP midline. Finally, we show that deep and outer cells move synchronously and in the same direction. Overall, our results show that the movement of the SE towards the midline is passive and is dependent on forces generated by NP morphogenesis. Similar mechanically coupled morphogenesis between neighbouring tissues has been described during early mouse development (Christodoulou et al., 2019), *Drosophila* development (Butler et al., 2009) and chick embryo axis elongation (Xiong et al., 2020).

In addition, in this study we examined how defective SE development might affect NTC. We found that thickening of the SE epithelium, after loss of Itgβ1 signalling, leads to NTDs. It has been suggested previously that Itgβ1 signalling is necessary for the migration of the deep ectodermal cells towards the midline (Morita et al., 2012). Here, we ruled out this possibility as we showed that the deep SE cells are not migratory. Thickening of the ectoderm in *Xenopus* embryos has been shown to result in tissue stiffening (Petridou et al., 2013). To examine the effect of SE tissue stiffening, we activated cell contractility optogenetically within the SE. This blocks the movement of the NP hinge points, revealing that a balance between the forces generated by the NP and the tension within the SE ensures proper NP development. Opposing forces by the surrounding tissues have been documented during NTC in mouse embryos (Galea et al., 2017). Our data directly show that increased tension within the SE prevents its deformation in response to NP-generated pulling forces and, as a result, mechanically opposes NTC. Similarly, overexpression of Grhl2 in mice results in an increase in tissue tension and defective NTC (Pyrgaki et al., 2011; Nikolopoulou et al., 2019).

Finally, we show that defective SE development affects both AC and CE within the NP. At the same time, PCP remains unaffected. This can be explained by the fact that the molecular regulators of actomyosin, which executes both AC and junction shrinkage during CE, have been shown to be mechanosensitive (Fernandez-Gonzalez et al., 2009; Pouille et al., 2009). It is likely that resistive forces stemming from the SE are higher than the capacity of actomyosin force generation by either CE or AC. In agreement with this, a recent report has demonstrated that ectopic induction of AC results in blockage of genetically programmed AC in a neighbouring cell population (Bhide et al., 2021). Thus, our results demonstrate the necessity of proper SE development for NTC and highlight the close mechanical linkage between the two tissues and the mechanosensitive nature of NP morphogenesis.

## MATERIALS AND METHODS

### *Xenopus* embryos and microinjections

Female adult *Xenopus laevis* frogs were induced to ovulate by injection of human chorionic gonadotropin. Eggs were fertilized *in vitro*, after acquisition of testes from male frogs, dejellied in 2% cysteine (pH 7.8) and subsequently reared in 0.1× Marc's Modified Ringers (MMR).

For microinjections, embryos were placed in a solution of 4% Ficoll in 0.33× MMR and injected using a glass capillary pulled needle, forceps, a Singer Instruments MK1 micromanipulator and Harvard Apparatus pressure injector at the four-cell stage according to Nieuwkoop and Faber (1967). After injections, embryos were reared for 1 h in 4% Ficoll in 0.33× MMR and then washed and maintained in 0.1× MMR. Injected embryos were allowed to develop to neurula stage (Nieuwkoop and Faber stage 12.5-13) at 17°C and imaged live or allowed to develop to the appropriate stage and the dissected or fixed in 1× MEMFA (Zanardelli et al., 2013) for 1-2 h at room temperature. Capped mRNAs encoding fluorescent protein fusions were *in vitro* transcribed using mMessage machine kits (Ambion). The amount of mRNA per 4 nl of microinjection volume was as follows: membrane-GFP, 100 pg; histone-RFP, 80 pg; Prickle2-GFP, 100 pg; Utrophin-RFP, 80 pg, mEos 100 pg.

### Morpholino oligonucleotides

The Shroom3 (Haigo et al., 2003), Vangl2 (Darken et al., 2002) and Itgβ1 (Morita et al., 2012) morpholinos (MOs) have been previously described and were obtained from Gene Tools, and 30 ng of Shroom3 MO, 20 ng of Vangl2 MO or 30 ng of Itgβ1 MO were injected per blastomere. Shroom3 and Vangl2 MOs were injected into dorsal blastomeres of four-cell-stage embryos to target the NP. Itgβ1 MO was injected into the ventral blastomere of four-cell-stage embryos to target the SE.

### Immunofluorescence

Immunofluorescence was performed as previously described (Zanardelli et al., 2013). Briefly, embryos were fixed for 2 h at room temperature, permeabilized in PBST (1×PBS, 0.5% Triton X-100, 1% dimethyl sulfoxide) and blocked for 1 h in 10% donkey serum. Embryos were then incubated overnight in primary antibodies at 4°C. Primary antibodies used were: integrin β1 (1:50, 8C8, Developmental Studies Hybridoma Bank) and Sox3 (1:100, DA5H6, Developmental Studies Hybridoma Bank). Embryos were washed in PBST and incubated for 2 h with secondary antibodies at room temperature, washed several times and post-fixed in 1× MEMFA. Alexa Fluor 488-conjugated secondary antibody was used (1:500, Invitrogen, A-21202), and co-incubated with Phalloidin 546 (1:500, Invitrogen, A22283).

### Deep SE migration assay

Explants of deep cell SE were dissected from stage 13 embryos. Dissections were performed in 1× MBSH [88 mM NaCl, 1 mM KCl, 2.4 mM NaHCO_3_, 0.82 mM MgSO_4_, 0.33 mM Ca (NO_3_)_2_, 0.33 mM CaCl_2_, 10 mM HEPES and 10 μg/ml Streptomycin and Penicillin, pH 7.4). Single cells were dissociated in alkaline buffer (88 mM NaCl, 1 mM KCl and 10 mM NaHCO_3_, pH 9.5) as previously described (Kashkooli et al., 2021). Dissociated cells were plated on FN-coated glass-bottom dishes and left to adhere for 45 min and then imaged.

### Nifedipine treatment

For nifedipine treatment, 200 µM nifedipine (from a 500× stock; Sigma-Aldrich) in DMSO was added to the medium at stage 13. Embryos were fixed at stage 25.

### ATP uncaging

DMNPE-caged ATP (A1049; Molecular Probes) was added to the medium to a concentration of 100 µM 1 h before imaging. For the photolysis of DMNPE-caged ATP, the samples were illuminated for 45 s with UV light. The duration of illumination was controlled manually.

### mEOS photoconversion

Embryos injected with 150 pg of mEOS mRNA. At neurula stage (stage 14), embryos were illuminated for 45 s with UV light at selected regions within the NP.

### Live imaging

Live imaging of neurula-stage *Xenopus* embryos was performed on a ZEISS LSM 710 confocal microscope with a Plan-Apo 10×, NA 0.45 objective for all time-lapse recordings except for Fig. 4D,E, Movies 11 and 12, for which we used a Plan-Apo 40×, NA 1.1 objective. The ZEISS ZEN software was used during imaging. Embryos were imaged in a custom chamber made of thick layer of vacuum grease on a microscope slide and sealed with a coverslip. Embryos were mounted in 0.1× MMR and kept at room temperature during imaging.

### Embryo explants and embryo wounding

Embryos injected with fluorescent proteins were cultured until stage 12-12.5 when embryos were devitellinized. For anterior and posterior NP explants, the posterior and anterior dorsal half regions of the embryo were explanted using a hair knife. For anterior NP/SE explants, the posterior dorsal half of the embryo was excised and the anterior dorsal half of the embryo together with the flanking SE was explanted. Explants were cultured in 0.1× MMR in Petri dishes and were immobilized using glass coverslips with silicon grease-coated edges. Explants were left to recover for at least 30 min before imaging.

To separate the anterior and posterior NP physically, we allowed the embryos to develop to stage 10 when the embryos were devitellinized using sharp forceps and then allowed to develop to stage 12.5. Subsequently, we used a hair knife to generate a wound of a rectangular shape (850 μm length across the ML NP axis and 120 μm width parallel to the A/P axis) at the boundary between the anterior and posterior NP. Wounded embryos were left to recover for 30 min before imaging.

### Image analysis and single-cell tracking

All image analysis and quantification were carried out using Fiji software (Schindelin et al., 2012). Particle image velocimetry (PIV) analysis was performed in Fiji using the in-built Iterative PIV (Cross-correlation) plugin. For single-cell tracking (Fig. 1), the spots function of the Imaris image analysis software was used. Automated generated tracks were manually curated. Neuroepithelial and SE lineages were defined based on retrograde tracking. Single-cell tracking in Figs 2D, 3C,D and 5C,G,I, and Figs S3B, S4A-C and S7C, was carried out using the manual tracking plugin of Fiji. Normalized fluorescent intensities in Figs 2F,H and 9E were quantified from sum intensity profile images. From these images, the fluorescent intensity of an ML junction was divided by the average signal intensity of two perpendicular A/P junctions (Fig. 2F). Junctions with an orientation of <45° relative to the ML axis of the embryo were selected as ML junctions and junctions with an orientation of >45° were set as A/P junctions.

### Statistics

GraphPad Prism 6.0 software was used for all statistical analysis performed. The sample size of the experiments carried out was defined based on previous experimental experience. Quantitative data are presented as mean±s.e.m. or the total number of data points obtained. The statistical tests carried out on the quantitative data obtained are stated in each figure legend.

## Supplementary Material

Supplementary information

Reviewer comments
