# Supplementary material for: Distinct spatiotemporal contribution of morphogenetic events and mechanical tissue coupling during Xenopus neural tube closure
Source: Development. 2022 Jul 1;149(13):dev200358. doi: 10.1242/dev.200358 (PMC9340557; doi:10.1242/dev.200358)
Supplement: Supplementary information [file develop-149-200358-s1.pdf]

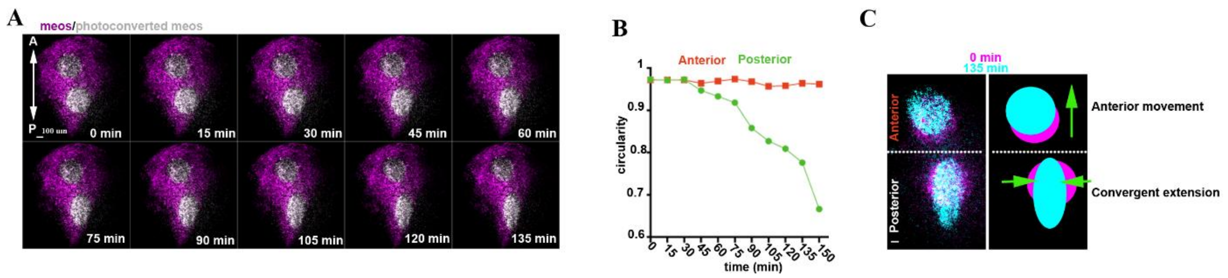


**Fig. S1. Distinct tissue behaviour along the anteroposterior neural plate axis.**

A) Stills from a time lapse recording of a neurula embryo expressing the photoconvertible fluorescent protein mEos. mEeos was photoconverted in two circular areas (0 min) at the posterior and anterior neural plate. The anterior neural plate region maintained its initial circular shape while the posterior region narrowed and extended. B) Quantification of anterior and posterior photoconverted regions circularity over time. C) Positions and shape of the posterior and anterior neural plate photoconverted regions at 0 and 135 min. The anterior region maintains its shape and moves towards more anterior regions while the posterior region narrows and extends. A: anterior. P: posterior. Scale bars: 100 um.

Development • Supplementary information

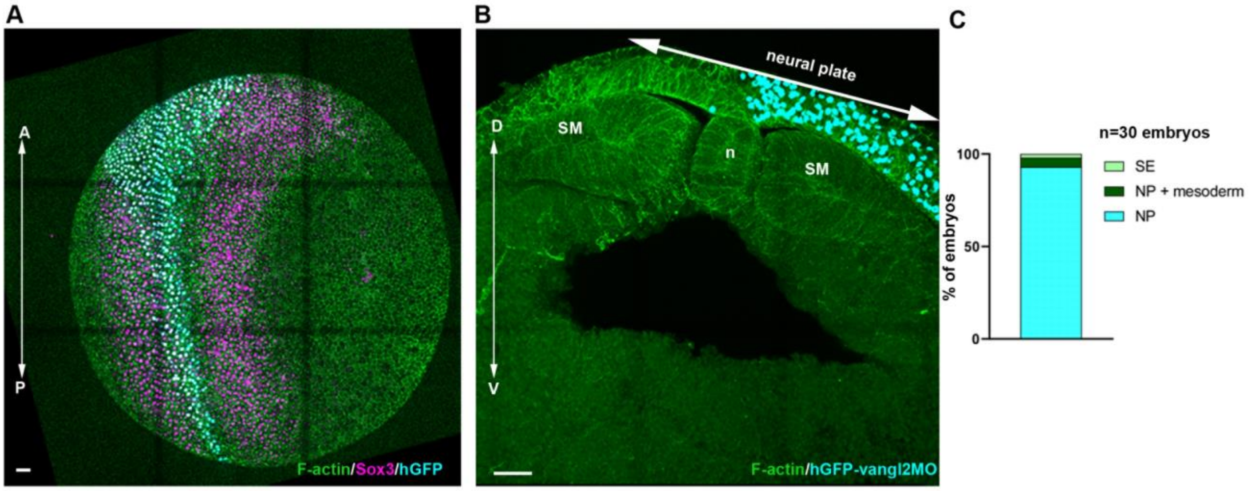


**Fig. S2. Targeting of the neurectoderm through microinjections.**

A) Representative image of a neurula stage embryo injected in one dorsal blastomere at the 4-cell stage with hGFP mRNA to target the neuroepithelium, marked by Sox3 expression. B) Cross section of a neurula stage Vangl2 morphant embryo targeted at the neuroepithelium by injection of 1 dorsal blastomere at the 4-cell stage. C) Quantification of lineage tracer distribution after microinjection of the 1 dorsal blastomere at the 4-cell stage. n=30 embryos. n: notochord, SM: somites. A: anterior. P: posterior. D: dorsal. V: ventral. Scale bars: 50um.

Development • Supplementary information

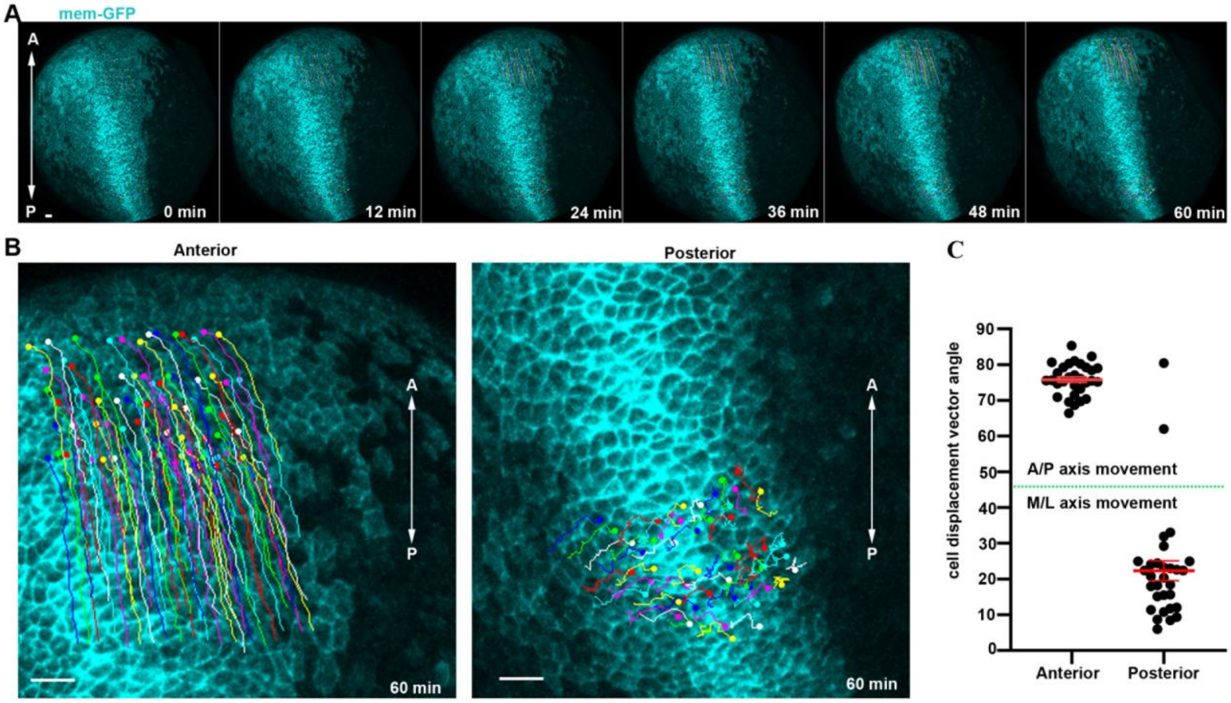


**Fig. S3. Anterior and posterior neural plate cell displacement.**

A) Stills from time lapse recording of a representative control embryo (presented in Figure 3C). B) Zoomed images of the anterior and posterior regions of A showing cell displacement over a period of 60 min as assessed by single cell tracking. Cells within the anterior move anteriorly while posterior cells display mediolateral movement. C) Quantification of cell displacement vector angle. Two-sided unpaired student's t-test; ****P<0.0001; mean±SEM; n=30 posterior and 30 anterior cells. A: anterior. P: posterior. Scale bars: 50 um.

Development • Supplementary information

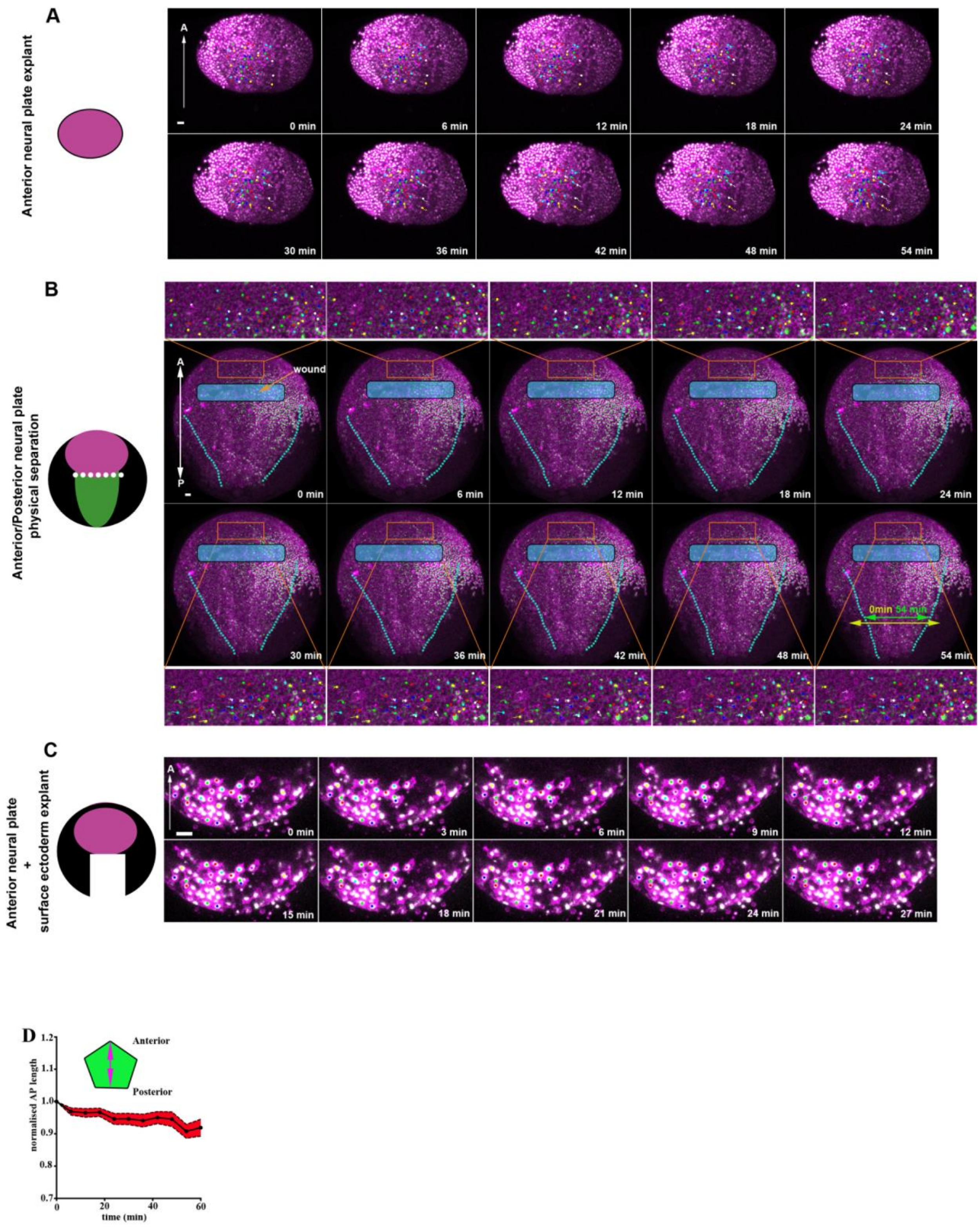


Development • Supplementary information

**Fig. S4. Physical coupling between the posterior and anterior neural plate is necessary for anterior tissue movement.**

A) Stills from a time lapse recording of an anterior neural plate explant. Single cell tracking shows that anterior neural plate cells don't move in comparison with their counterparts in the embryo (Figure 3 C). B) Stills from a time lapse recording of an embryo with a deep wound (cyan rectangle) physically separating the anterior and posterior neural plate. Single cell tracking (zoomed images) reveals that anterior neural plate cells do not display anterior directed movement when physically separated from the posterior tissue. Double arrowheads indicate the width of the posterior neural plate at 0 and 54 minutes. C) Stills from a time lapse recording of an anterior neural plate/surface ectoderm explant. Single cell tracking shows that anterior neural plate cells do not display anterior directed movement. D) Quantification of anteroposterior cell length (magenta double headed arrow) over time, of posterior NP cells located at the anterior/posterior region boundary. mean±SEM (red coloured area), n=30 cells for each time point. A: anterior. P: posterior. Scale bars 50um.

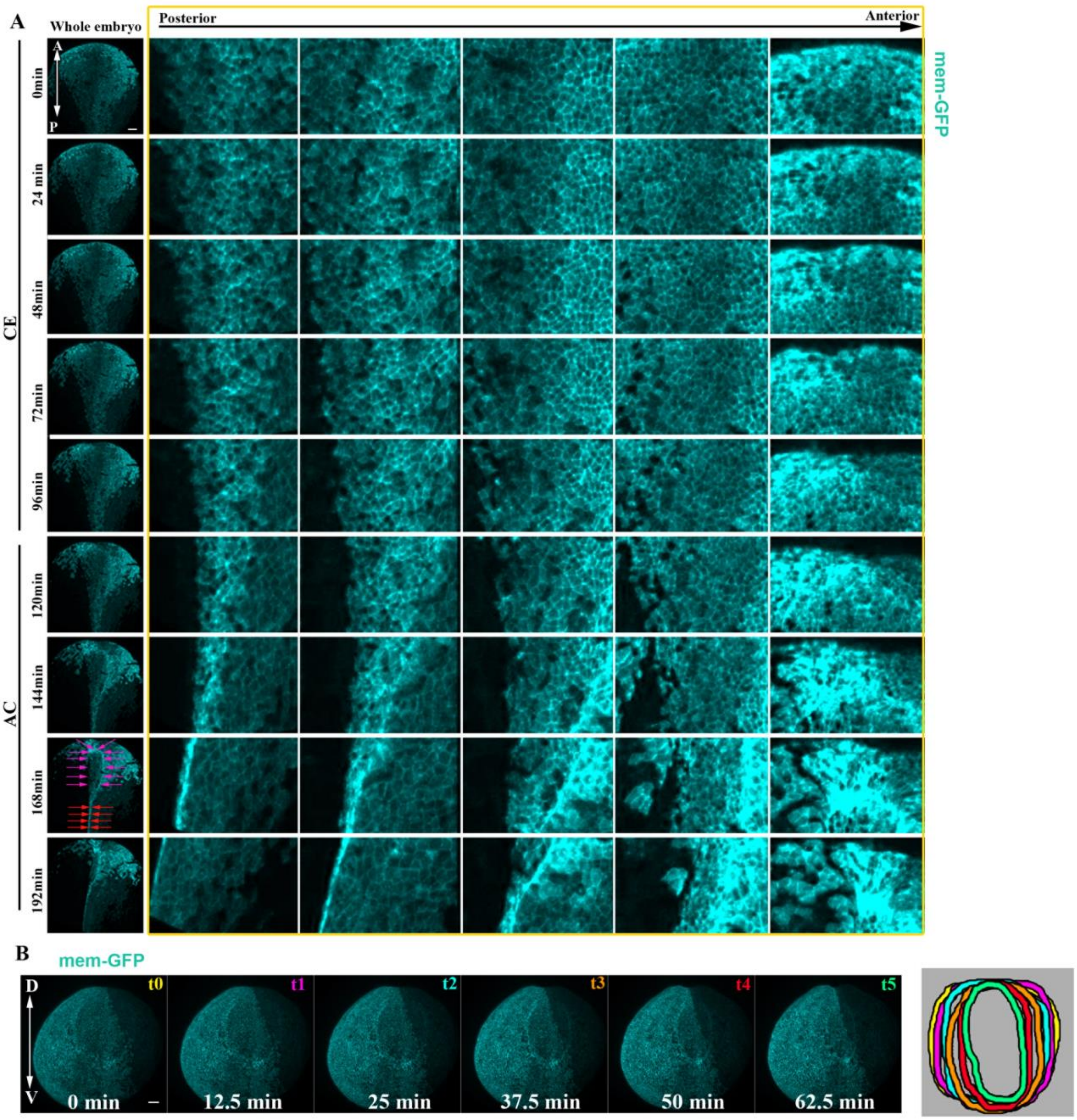


**Fig. S5. Progression of neural tube closure along the anteroposterior axis of the embryo**

A) Stills from a time lapse recording of a neurula embryo. In the left panel row, the whole embryo is shown. The next five rows show zoomed neural plate areas (caudal to rostral from left to right). Convergent extension at the posterior neural plate precedes apical constriction. Caudal neural tube closure (red arrows) is completed before anterior neural tube closure (magenta arrows). B) Stills from a time lapse recording of an embryo expressing membrane-GFP. Right panel shows the shape (temporal colour coded) of the rostral neural plate during neural tube closure indicating absence of convergent extension. The anterior neural plate shrinks along the ML axis due to apical constriction. A: anterior. P: posterior. D: dorsal. V: ventral. Scale bars = 100um.

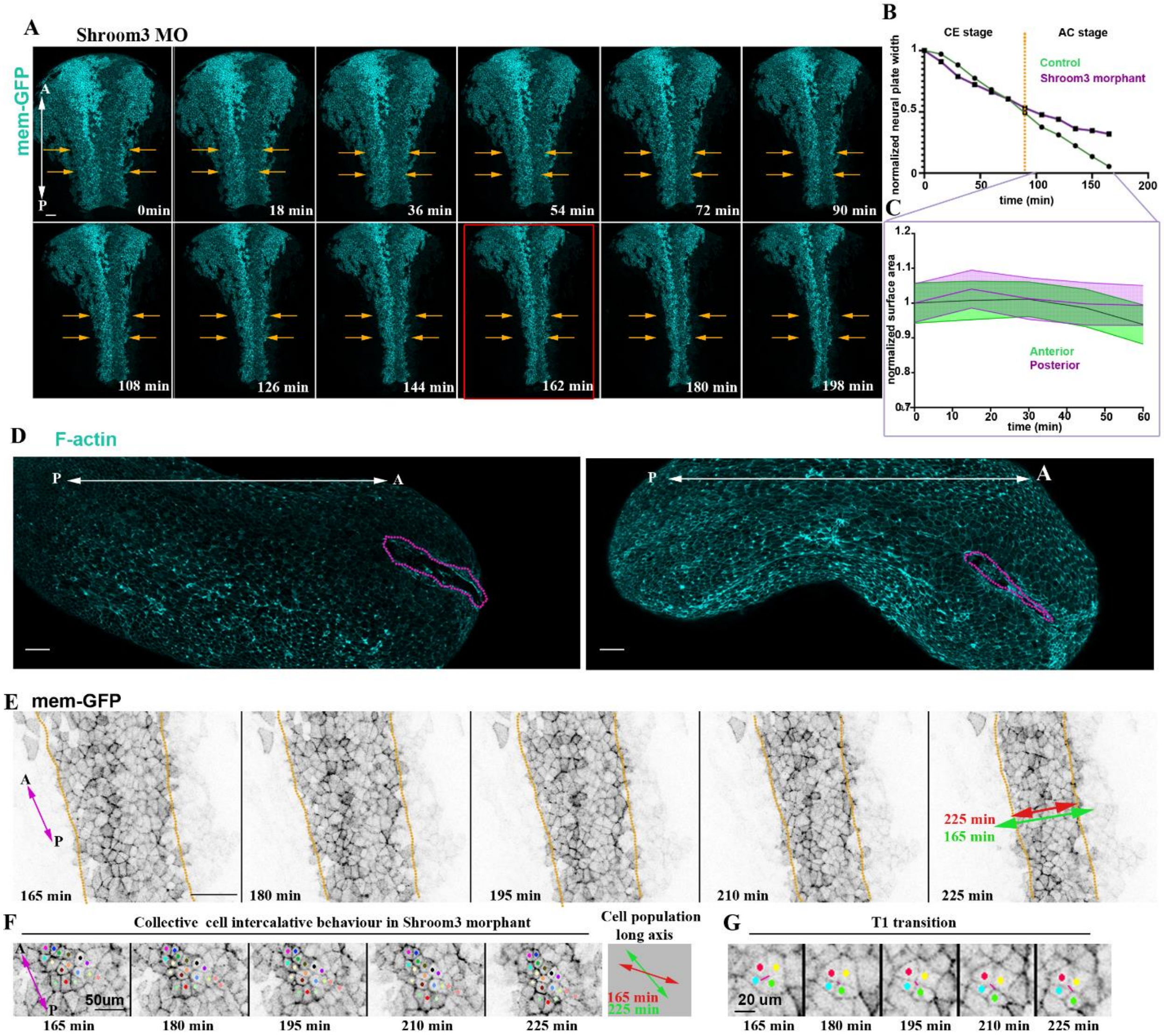
A
Shroom3 MO
mem-GFP
A
P
0min
18 min
36 min
54 min
72 min
90 min
108 min
126 min
144 min
162 min
180 min
198 min
B
CE stage
AC stage
Control
Shroom3 morphant
normalized neural plate width
time (min)
C
normalized surface area
Anterior
Posterior
time (min)
D
F-actin
P
A
E
mem-GFP
225 min
165 min
180 min
195 min
210 min
F
Collective cell intercalative behaviour in Shroom3 morphant
50um
Cell population long axis
G
T1 transition
20 um

**Fig. S6. Apical constriction is necessary for the second phase of neural tube closure.**

A) Stills from a time lapse recording of a Shroom3 morphant embryo. The caudal neural plate narrows (arrows) and lengthens through convergent extension. By the time-point that control embryos have completed neural tube closure (red box), neural tube closure is not completed, and caudal neural plate continues to narrow. B) Quantification of posterior NTC rate (normalized NP width over time) in a control and Shroom3 morphant embryo. During the CE phase NTC rate is not affected by Shroom3 downregulation. Shroom3 downregulation affects NTC only during AC. C) Quantification of normalized surface area over time of posterior and anterior neural plate cells during a time period when AC normally occurs. NP cell surface area is not reduced in Shroom3 morphant embryos, indicating that AC is defective. n= 30 posterior and 30 anterior neural plate cells. D)Representative embryos (stage 25) treated with 200uM nifedipine from stage 14. The anterior neural tube failed to close (dashed outline). n=20 embryos. E) Zoomed stills from the same time lapse recording as in (A), starting from the time point highlighted with red box in (A), when neural tube closure is completed in control embryos. Dashed lines: neural plate boundaries. Neural plate convergent extension continues well beyond the time point of neural tube closure completion in control embryos. Double headed arrows: neural plate width at different time points. F) Single cell behavior analysis from a zoomed region from (E) showing polarized cell intercalative behavior and neighbour exchanges (red and orange marked cell as an example). Right panel: The cell population long axis reorients as time progresses, becoming parallel with the embryo's AP axis. G) An example of T1 transition from (C). The remodelled cell junction is highlighted with magenta. A: anterior. P: posterior. Scale bars for A, B and C = 100um, for D= 50um and for E=20um.

Development • Supplementary information

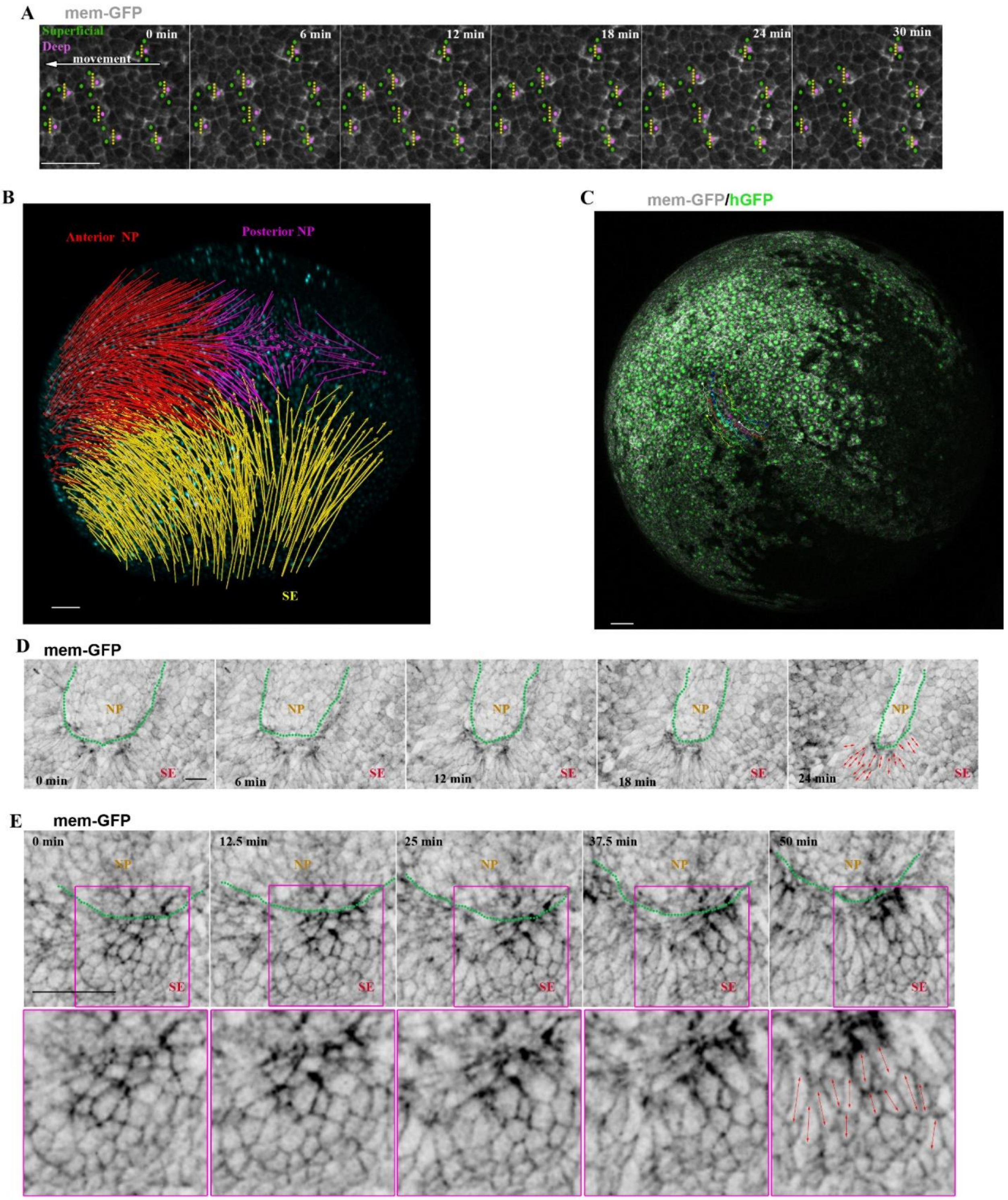
A
mem-GFP
Superficial
Deep
movement
0 min
6 min
12 min
18 min
24 min
30 min
B
Anterior NP
Posterior NP
SE
C
mem-GFP/hGFP
D
mem-GFP
NP
SE
0 min
6 min
12 min
18 min
24 min
E
mem-GFP
0 min
12.5 min
25 min
37.5 min
50 min
NP
SE

**Fig. S7. Surface ectoderm movement is coupled with neural plate morphogenesis.**

A) Stills from a time lapse recording of a control embryo expressing mem-GFP showing the surface ectoderm (SE). Superficial SE cells are marked with green spots and deep SE cells with magenta dots. Note that as neural tube closure progresses the deep SE cells move together with superficial cells and never overtake superficial cells. B) Displacement map of single cell tracks coloured according to their spatial location. The movement of SE cells mirrors the movement of neuroepithelial cells. C) Representative example of rostral/ ventral SE cell's displacement in a 60 min time window during the first phase of neural tube closure, assessed by single cell tracking. The cells move towards the rostral and ventral side of the embryo. D)Stills from a time lapse recording focusing on the anterior neural plate (dashed line). During anterior neural plate folding (second phase of neural tube closure) the SE cells beneath the neural plate(NP) are stretched and acquire an elongated shape (double headed arrows). E) Stills from a time lapse recording of an embryo focusing on the anterior neural plate (dashed line). During anterior neural plate folding the SE cells beneath the neural plate are stretched and acquire an elongated shape (double headed arrows). Scale bars = 100um.

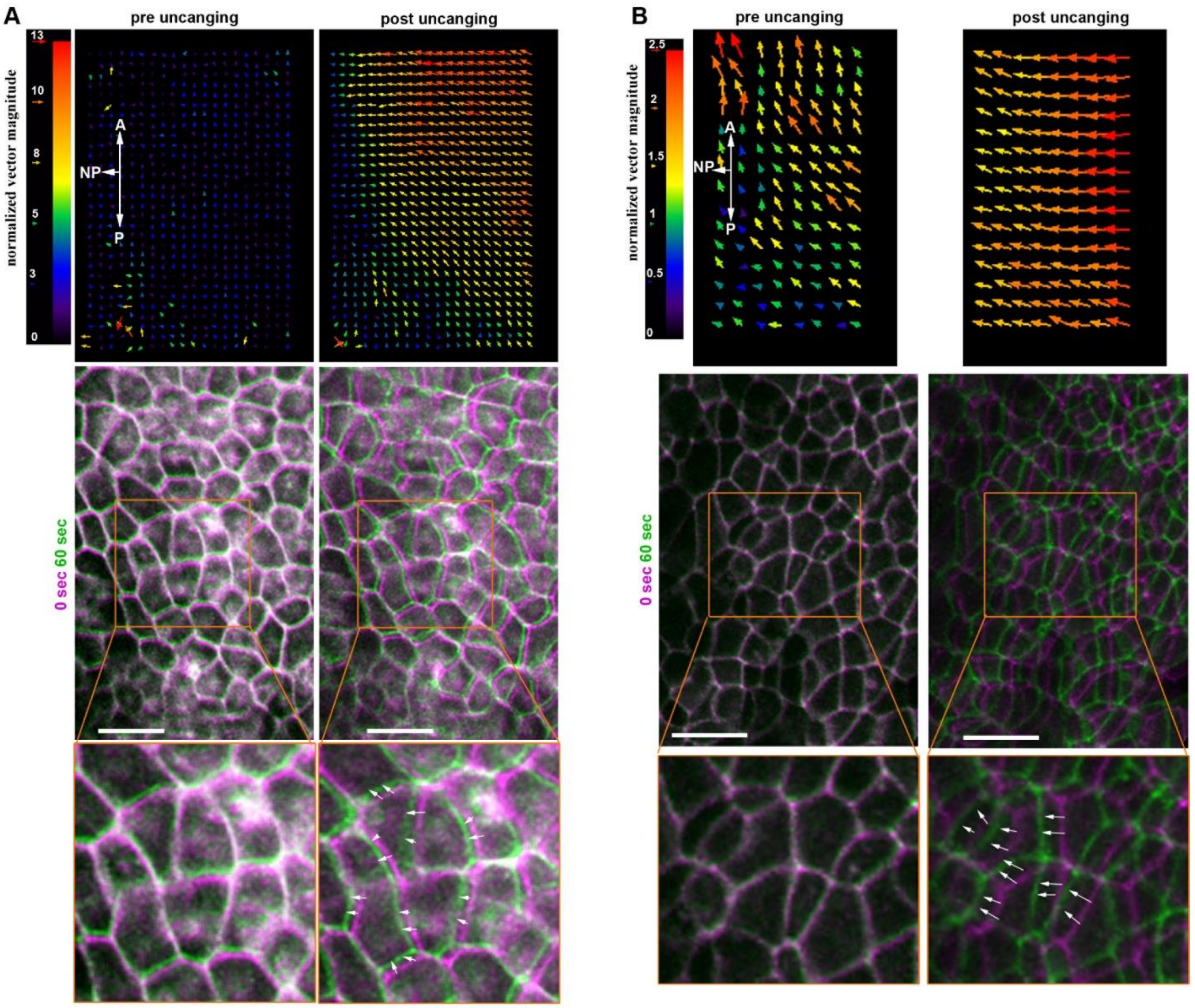


**Fig. S8. Force generation within the neural plate is sufficient to drive the movement of the surface ectoderm.**

A,B) Particle image velocimetry (PIV) analysis of two different embryos used for quantification of surface ectoderm vector magnitude in Figure 6. Top panels: PIV analysis illustrating increased movement of SE movement upon ATP uncaging within the NP. Bottom panels: Time colour-coded representative images of the SE before and after NP targeted ATP unchanging. Arrows indicate the movement of SE between the two time points. A: anterior. P: posterior. NP: neural plate. Scale bars: 50um

Development • Supplementary information

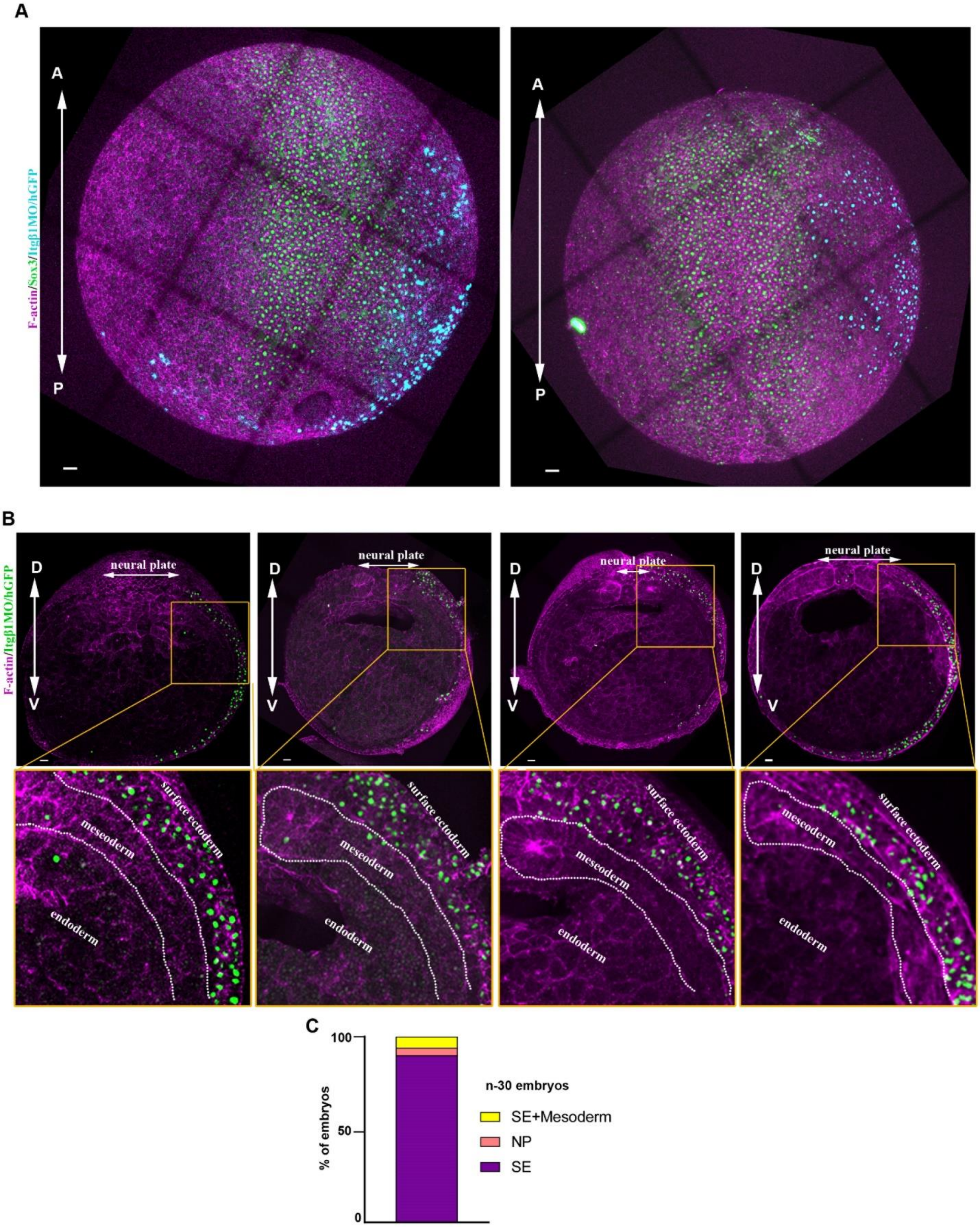

A
F-actin/Sox3/Itgβ1MO/hGFP
A
P
B
F-actin/Itgβ1MO/hGFP
D
V
neural plate
surface ectoderm
meseoderm
endoderm
C
% of embryos
100
50
0
n-30 embryos
SE+Mesoderm
NP
SE

**Fig. S9. Surface ectoderm targeting via microinjections.**

A) Representative examples of embryos injected with Itgb1 morpholino. Injections were targeted at the one ventral blastomere of 4 cell stage embryos leading to surface ectoderm targeting. Sox3 staining marks the neurectoderm. B) Cross section of a neurula stage Itgb1 morphant embryo targeted at the surface ectoderm by the injection of 1 ventral blastomere at the 4-cell stage. C) Quantification of lineage tracer distribution after microinjection of the 1ventral blastomere at the 4-cell stage. A: anterior. P: posterior. D: dorsal. V: ventral. Scale bars: 50um

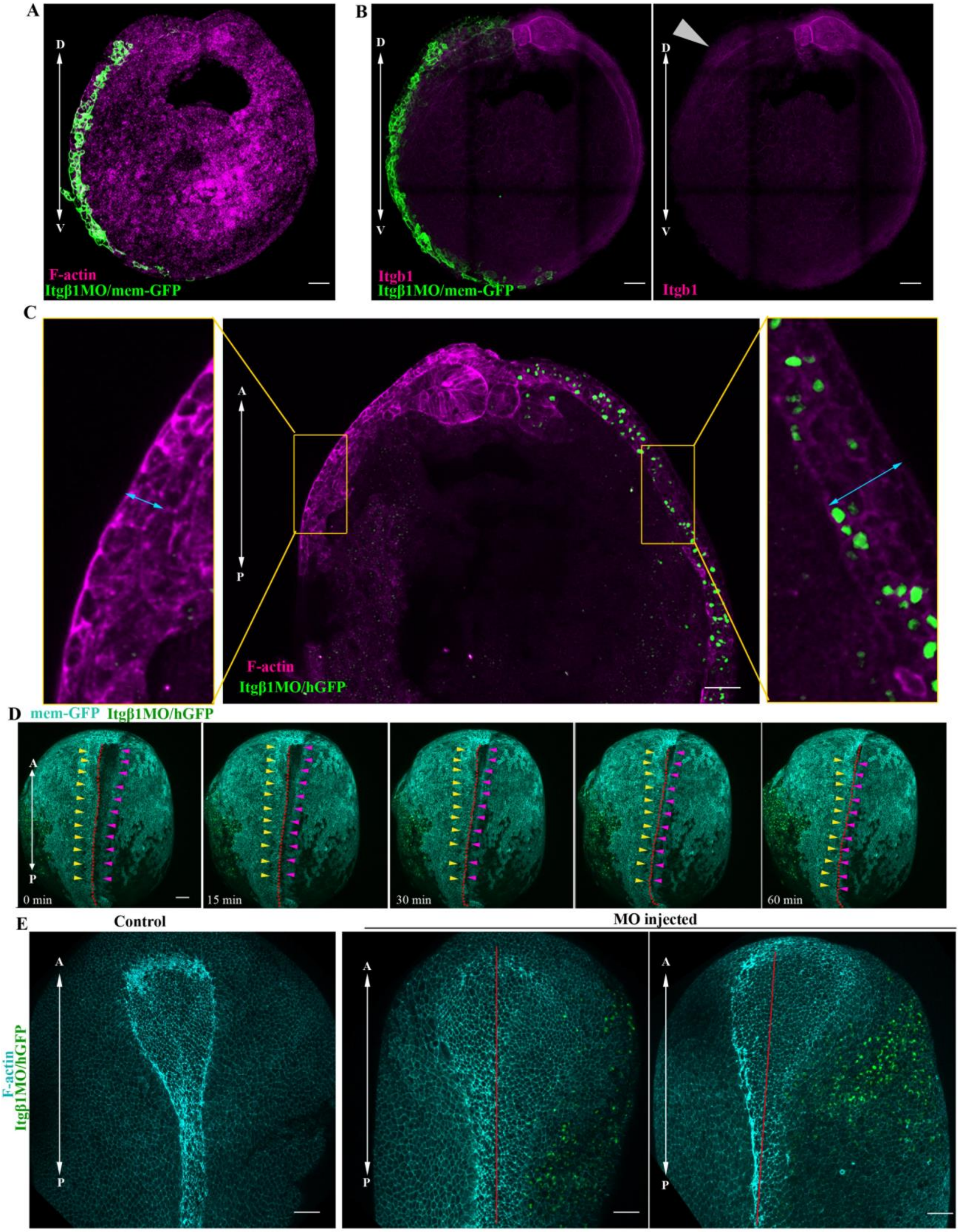


Development • Supplementary information

**Fig. S10. Normal surface ectoderm homeostasis is necessary for neural tube closure.**

A) Cross section of a representative embryo. Microinjection of Itgβ1 MO+ membrane GFP at the animal side of 1 ventral blastomere at the 4-cell stage led to unilateral SE targeting. B) Cross section of representative embryo unilaterally targeted with Itgβ1 MO at the SE showing effective downregulation of Itgβ1 at the MO injected side (arrowhead). C) Representative example from a cross section of an embryo with SE unilateral injection of Itgβ1 MO. Itgβ1 MO was co-injected with histone-GFP. Zoomed images of the SE reveal that the thickness of the SE (double headed arrow) is increased at the Itgβ1 morphant side. D) Stills from a time lapse recording of a unilateral SE targeted Itgβ1 morphant embryo. Neural tube closure at the side adjacent to the Itgβ1 morphant SE is defective. Arrowheads indicate the neural folds (magenta: control; yellow: Itgβ1 morphant side). The neural fold at the Itgβ1 morphant side fails to reach the midline (dashed red line) E) Representative examples of a control embryo and two unilateral SE targeted Itgβ1 morphant embryos. Red line indicates the midline. A: anterior. P: posterior. D: dorsal. V: ventral. Scale bars = 100um.

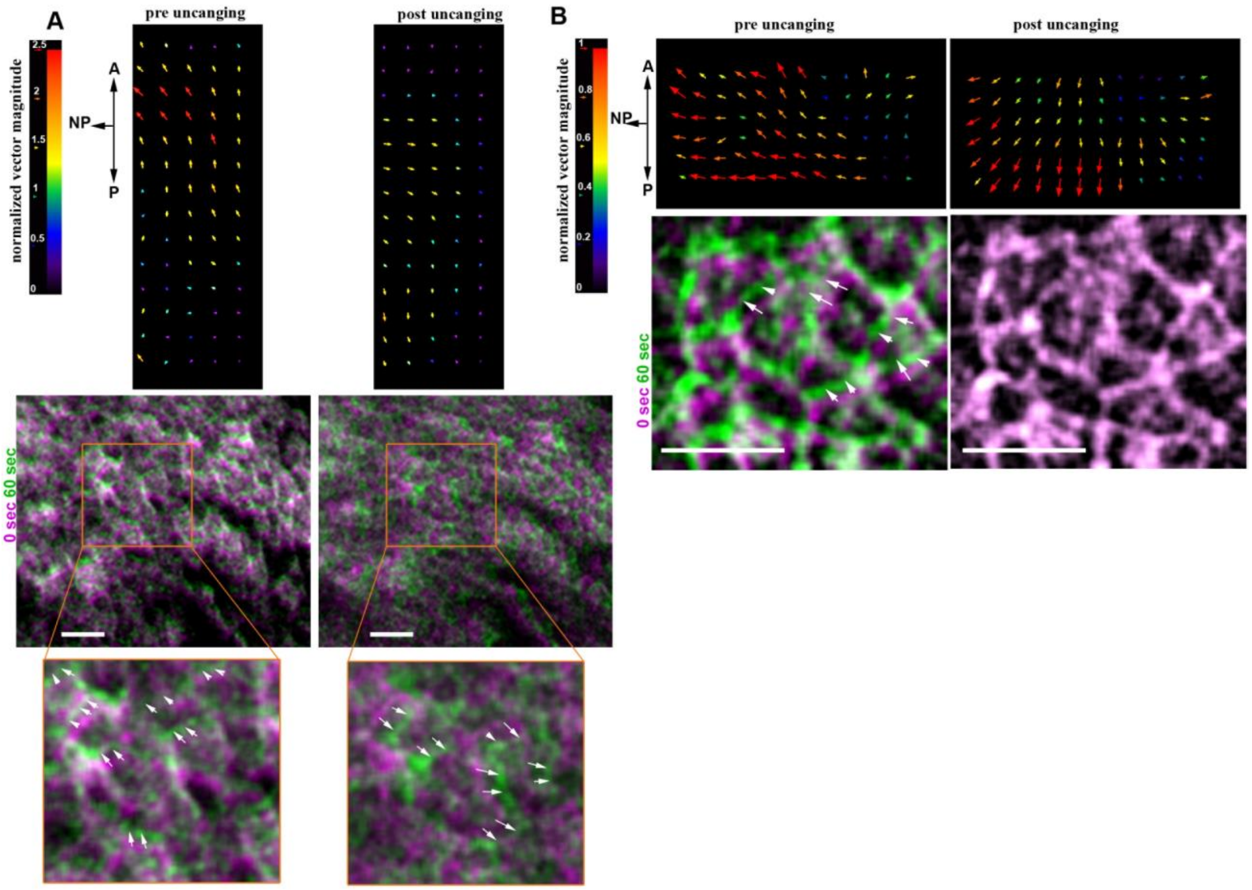


**Fig. S11. Increased surface ectoderm contractility negatively impacts neural tube closure.**

A,B) Particle image velocimetry (PIV) analysis of two different embryos used for quantification of vector magnitude movement angle in Figure 8. Top panels: PIV analysis illustrating reversal of SE movement upon ATP uncaging within the SE. Bottom panels: Time colour-coded representative images of the SE before and after SE targeted ATP unchanging. Arrows highlight the reversal of SE movement. A: anterior. P: posterior. NP: neural plate. Scale bars 50 um.

Development • Supplementary information

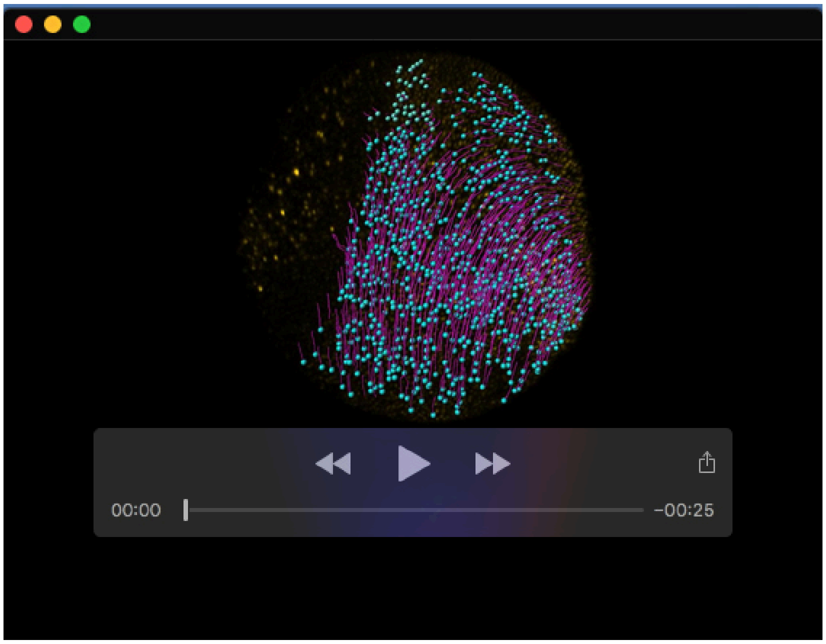


**Movie 1. Single cell tracking during Xenopus laevis neurulation.** Single cell tracked time lapse recording of a neurula stage embryo expressing histone-GFP. In the first part of the Movie all neuroepithelial cells tracks are shown with cyan spots and magenta lines. During the second part of the Movie the neuroepithelial cells are colour coded according to their spatial location within the neural plate. Cyan dots: caudal neural plate. Red dots: rostral neural plate. Time interval = 3 min.

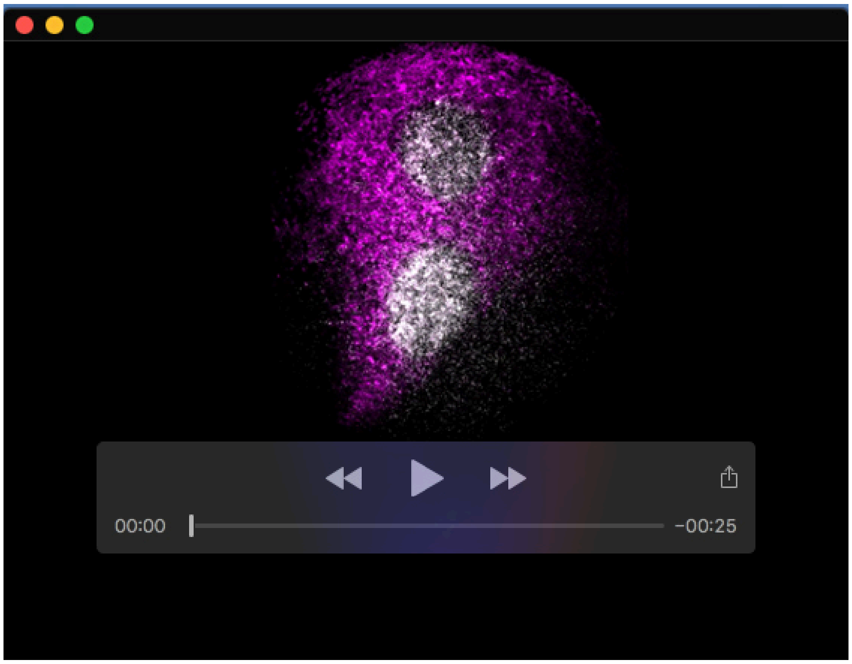


**Movie 2. Anterior and posterior neural plate deformation.**Time lapse recording of a neurula stage embryo expressing mEos (magenta). mEos was photoconverted at two circular regions (white) are the anterior (top) and posterior (bottom) neural plate, used to follow tissue displacement and deformation over time. Time interval=1 min.

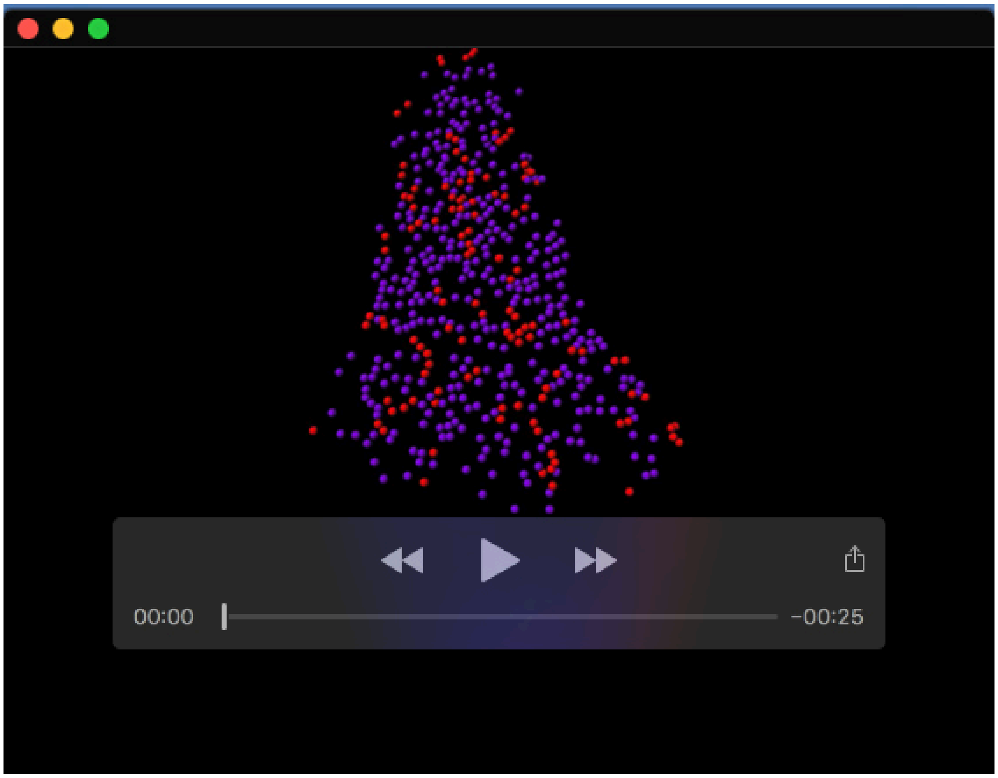


**Movie 3. Cell division events during neurulation.** Generation colour coded cell tracks of neuroepithelial cells during neural tube closure. Proliferation is uniform within the neural plate during neurulation. Time interval = 3 min.

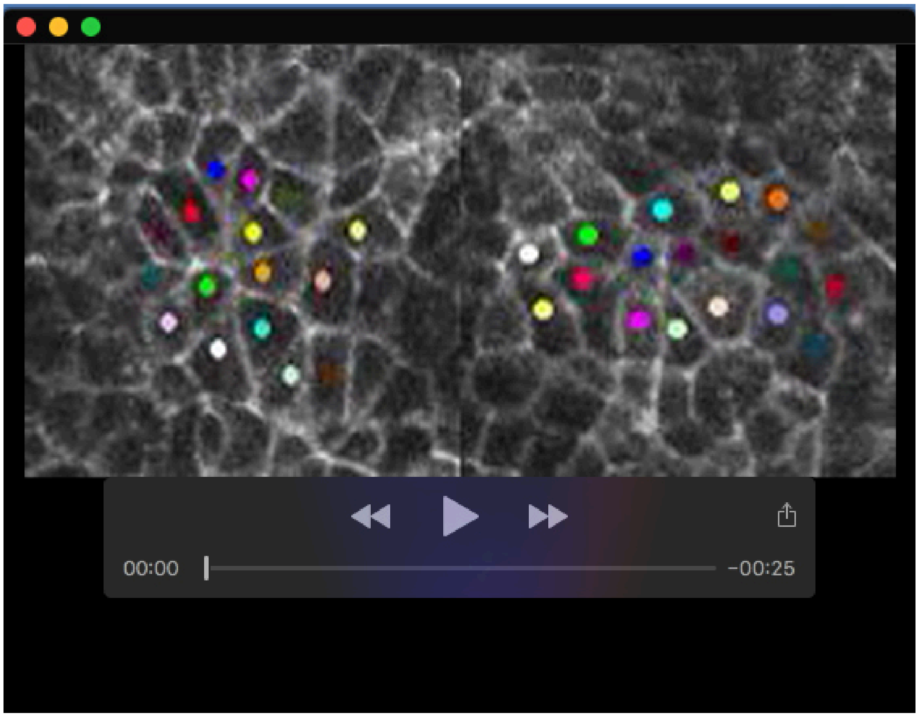


**Movie 4. Intercalative behaviour along the anteroposterior neural plate axis.** Time lapse recording of the posterior (left) and anterior (right) regions of the neural plate of an embryo expressing mem-GFP. Neuroepithelial cells display polarised intercalative behaviour only at the caudal part of the tissue. Time interval = 3min.

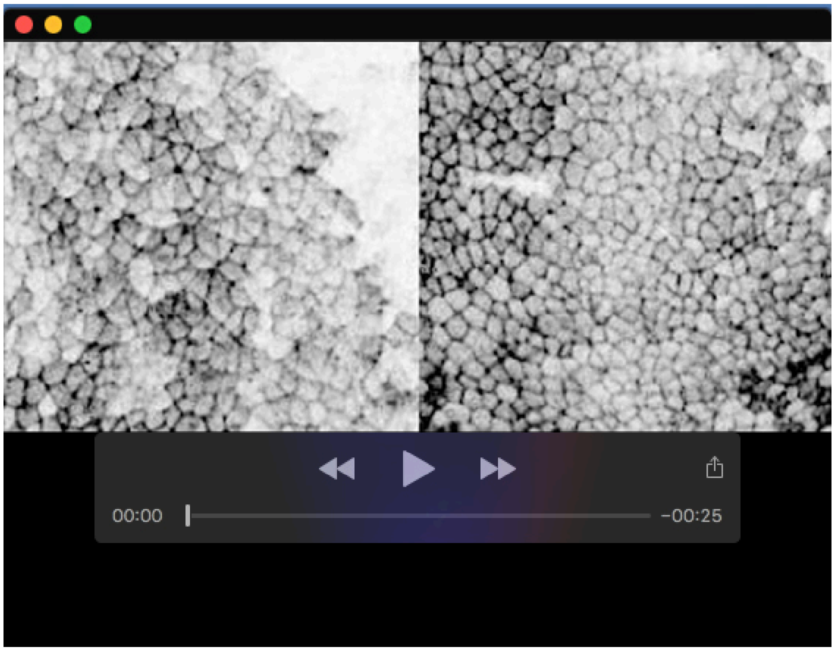


**Movie 5. Distinct cell behaviour along the anteroposterior axis of the neural plate.**Part 1: Caudal (left) and rostral (right) morphogenesis neural plate morphogenesis from an embryo expressing mem-GFP. Only the caudal neural plate undergoes convergent extension Part 2: Single cell tracking of neuroepithelial cells reveals the presence of cell intercalation at the caudal neural plate (left) and an anterior directed movement of the rostral part of the tissue (right). Time interval= 3 min.

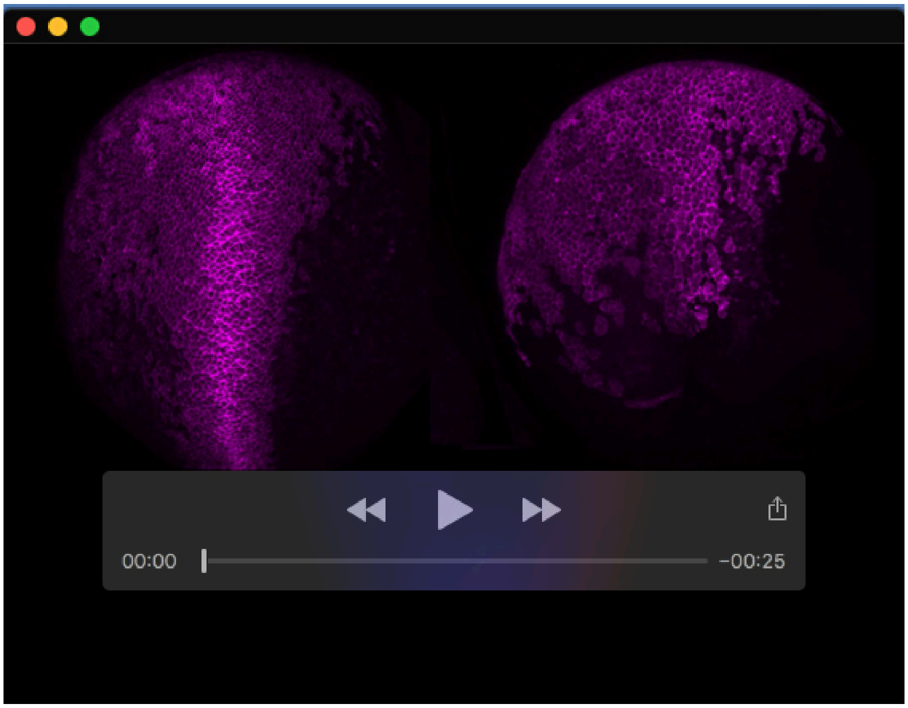


**Movie 6. Vangl2 downregulation leads to defective convergent extension.** Time lapse recording of a control (left) and a Vangl2 morphant embryo. Neural plate convergent extension is defective in Vangl2 morphant embryo. Time interval= 3min

Development • Supplementary information

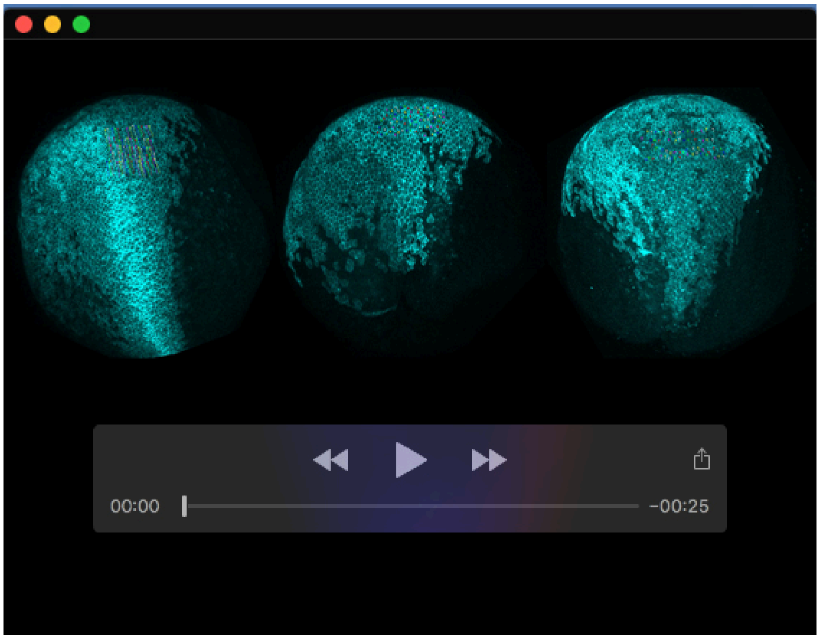


**Movie 7. Convergent extension is necessary for the directed movement of the anterior neural plate.** Tracking of anterior neuroepithelial cells in control (left) and Vangl2 morphant embryos (2 right embryos). The anterior directed movement of rostral neuroepithelial cells is absent in Vangl2 morphant embryos. Time interval= 3min

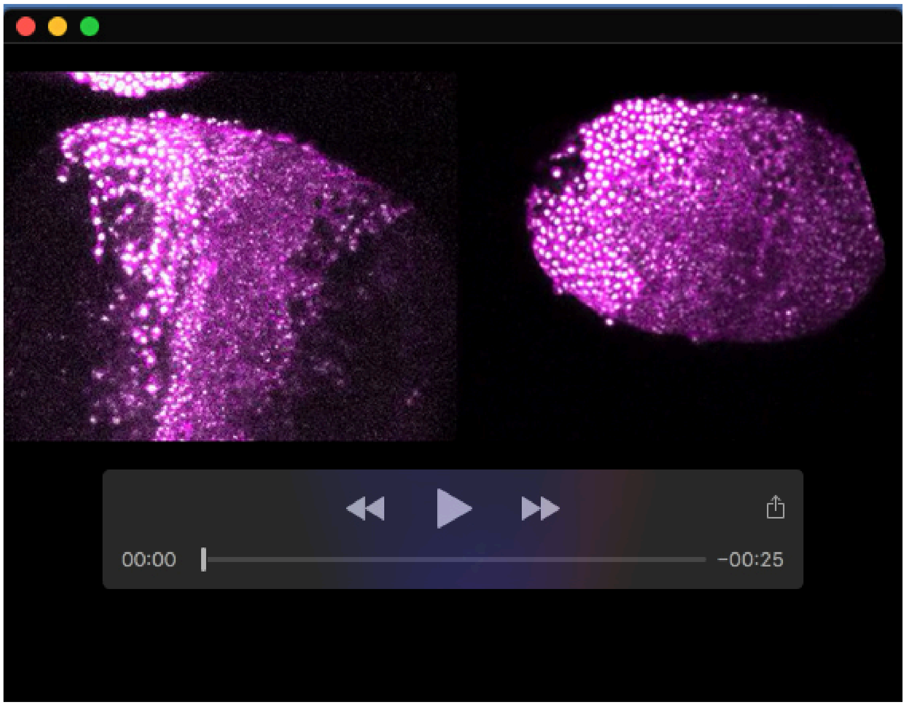


**Movie 8. Physical coupling between the posterior and anterior neural plate is necessary for anterior neural plate directed movement.** Time lapse recording of a posterior neural plate explant (left) and an anterior neural plate explant. Note that during deformation of the posterior neural plate explant due to convergent extension, the anterior neural plate explants does not display directed forward movement towards the anterior (top). Magenta: mem-GFP, Grey: Histone-RFP. Time interval: 3 min

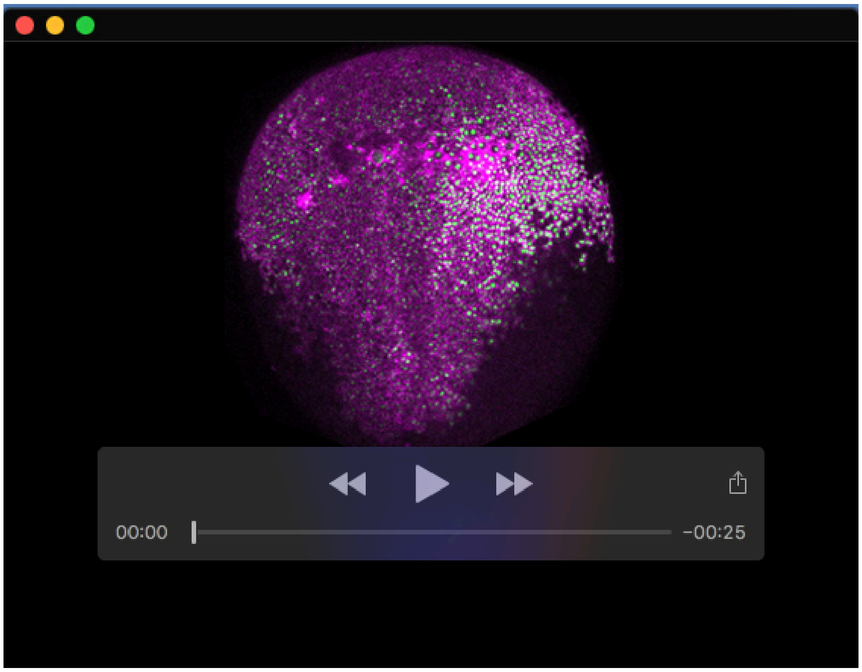


**Movie 9. Physical separation of the anterior and posterior neural plate results in defective anterior neural plate movement.** Time lapse recording of a neural stage embryo with a wound at the anterior/posterior neural plate boundary. The posterior (bottom) neural plate narrows through convergent extension. The anterior neural plate cells (top) don't display forward anterior directed movement. Magenta: mem-GFP, Grey: Histone-RFP: Time interval: 1 min.

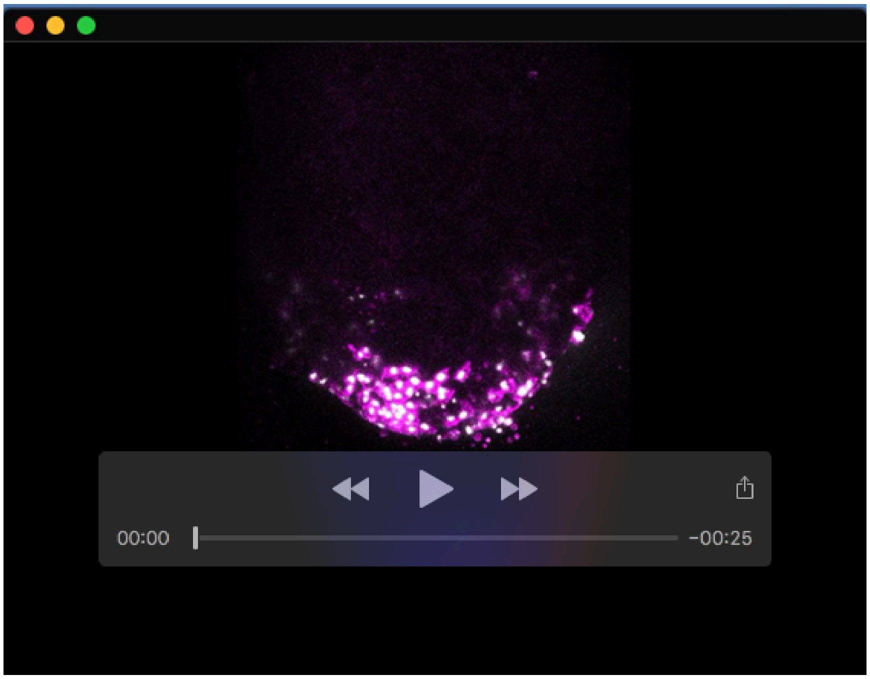


**Movie 10. Anterior neural plate does not display directed movement in the absence of the posterior neural plate.** Time lapse recording of an anterior neural plat (fluorescently labelled)+ surface ectoderm explant during neurulation. In the absence of the posterior neural plate, the anterior neural plate (fluorescently labelled cells) does not display anterior (top) directed movement. Time interval: 1 min.

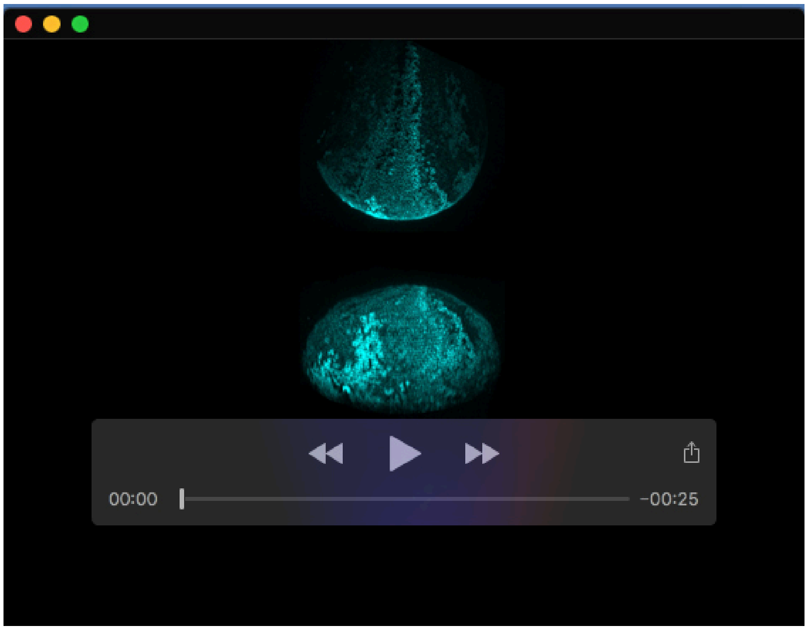


**Movie 11. Neural tube closure (1)**. Neural tube closure at the posterior (top) and rostral neural plate(bottom). Initially, caudal neural plate undergoes convergent extension and anterior neural plate moves towards the dorsoventral midline. Subsequently apical constriction occurs both at the posterior and anterior neural plate. Posterior neural tube closure is completed before anterior neural tube closure. Time interval= 3min

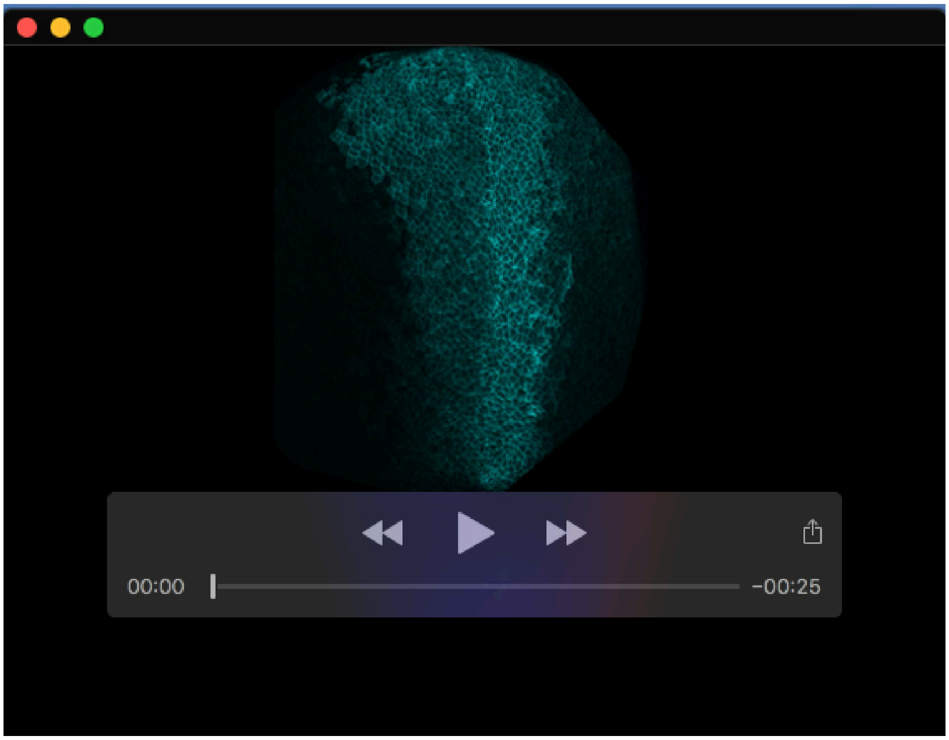


**Movie 12. Neural tube closure (2).** Initially, caudal neural plate undergoes convergent extension and rostral neural plate moves towards the dorsoventral midline. Subsequently apical constriction occurs both at the caudal and anterior neural plate. Caudal neural tube closure is completed before rostral neural tube closure. Time interval= 3min

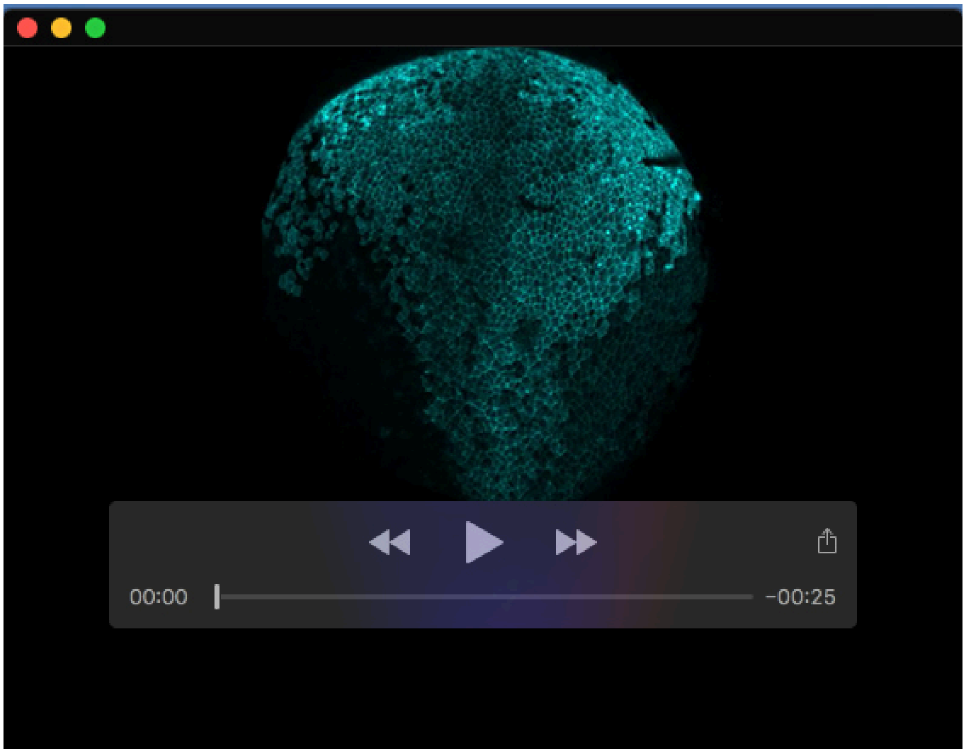


**Movie 13. Neural tube closure (3)**. Initially, caudal neural plate undergoes convergent extension and rostral neural plate moves towards the dorsoventral midline. Subsequently apical constriction occurs both at the caudal and anterior neural plate. Caudal neural tube closure is completed before rostral neural tube closure. Time interval= 3min

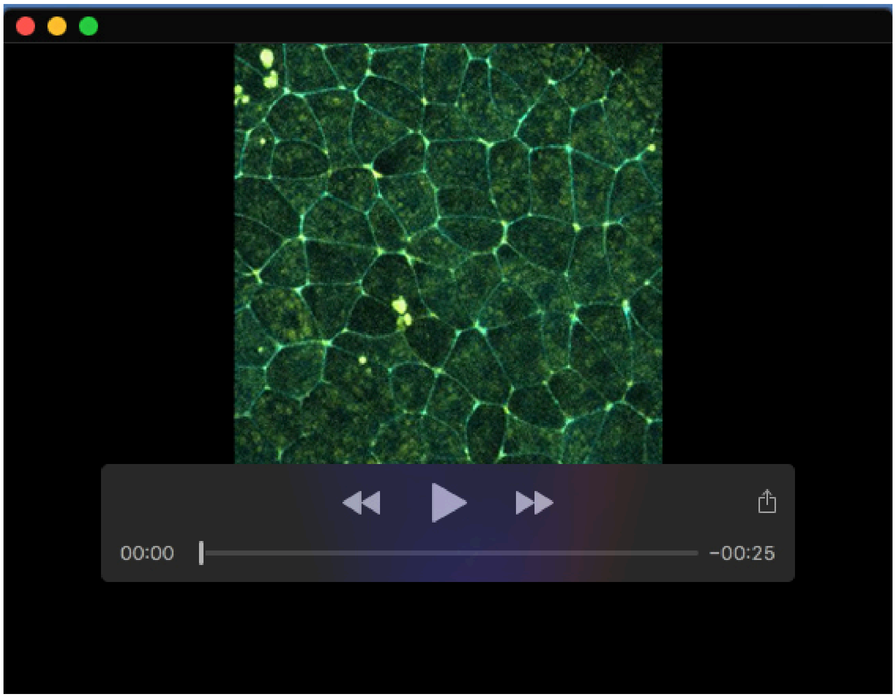


**Movie 14. Convergent extension and apical constriction do not show temporal overlap during neural tube closure.** Time lapse recording showing cell behaviour at the posterior neural plate from stage 12.5-stage 16. Initially neuroepithelial cells undergo polarised cell intercalation. During the last phase of neural tube closure, neuroepithelial cells undergo apical constriction, marked by the reduction of their apical cell surface area and enrichment of medio-apical actin signal (yellow). Cyan: Prickle2, Yellow: Utr-GFP. Time interval= 30 sec.

Development • Supplementary information

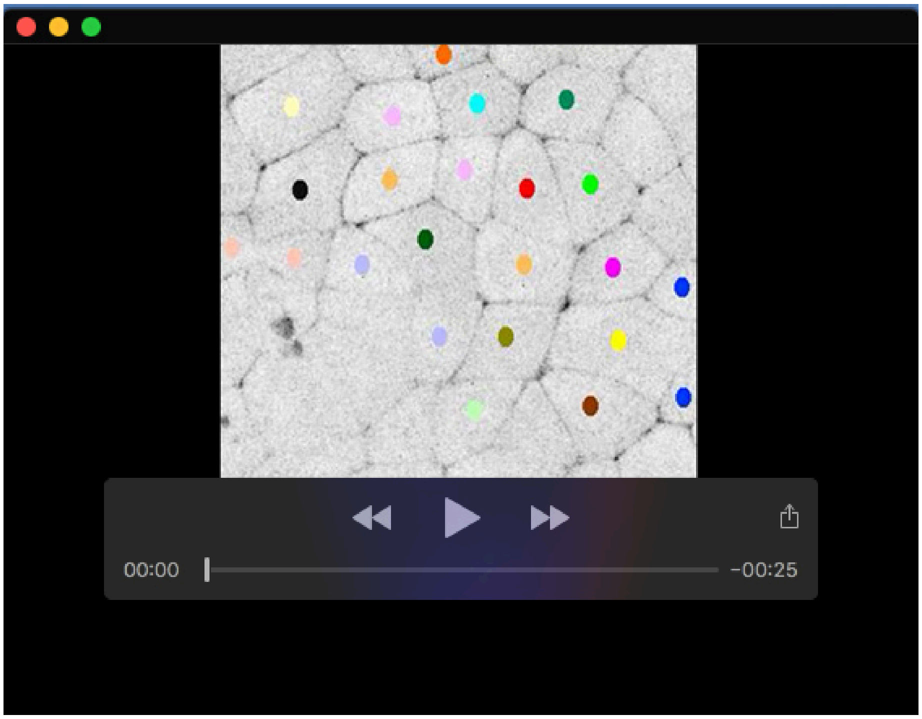


**Movie 15.** Manual single cell tracking of a zoomed region from Movie 14 using the PK2-GFP signal. Neighbour exchanges are present during convergent extension but not during apical constriction. Time interval= 12.5 min.

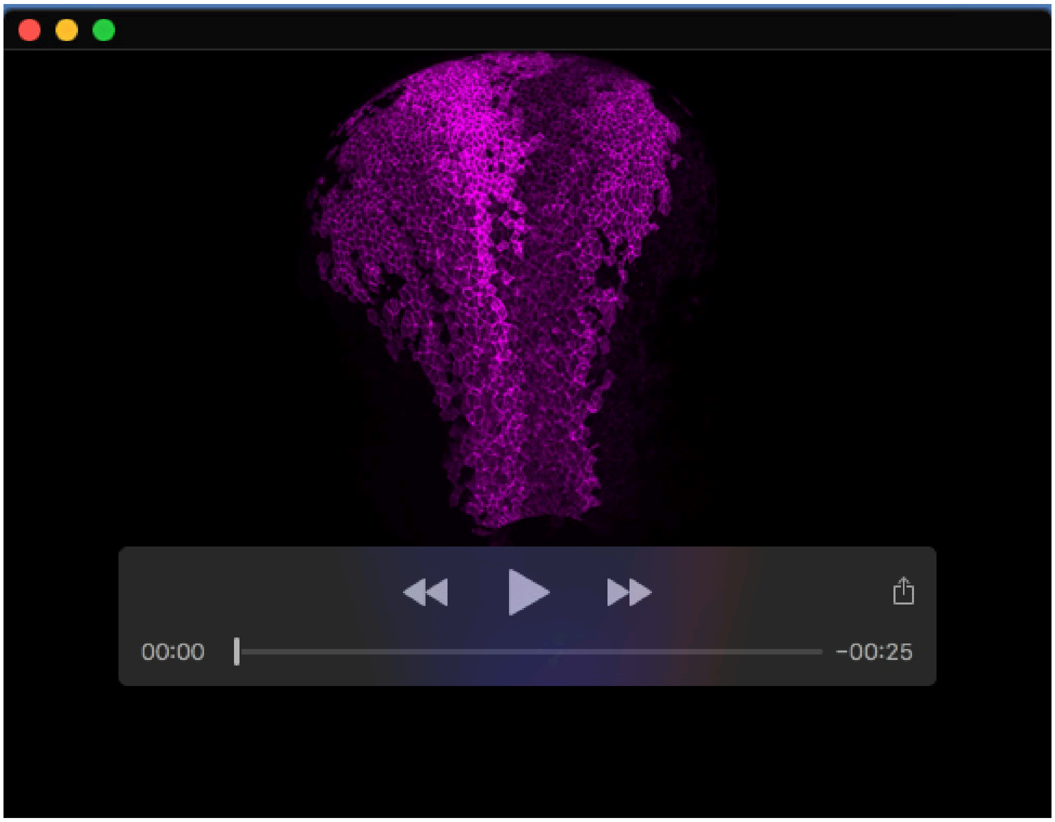


**Movie 16. Shroom3 downregulation results in defective apical constriction.** Time lapse recording of a Shroom3 morphant embryo. Caudal neural plate convergent extension is not affected. Time interval= 3min

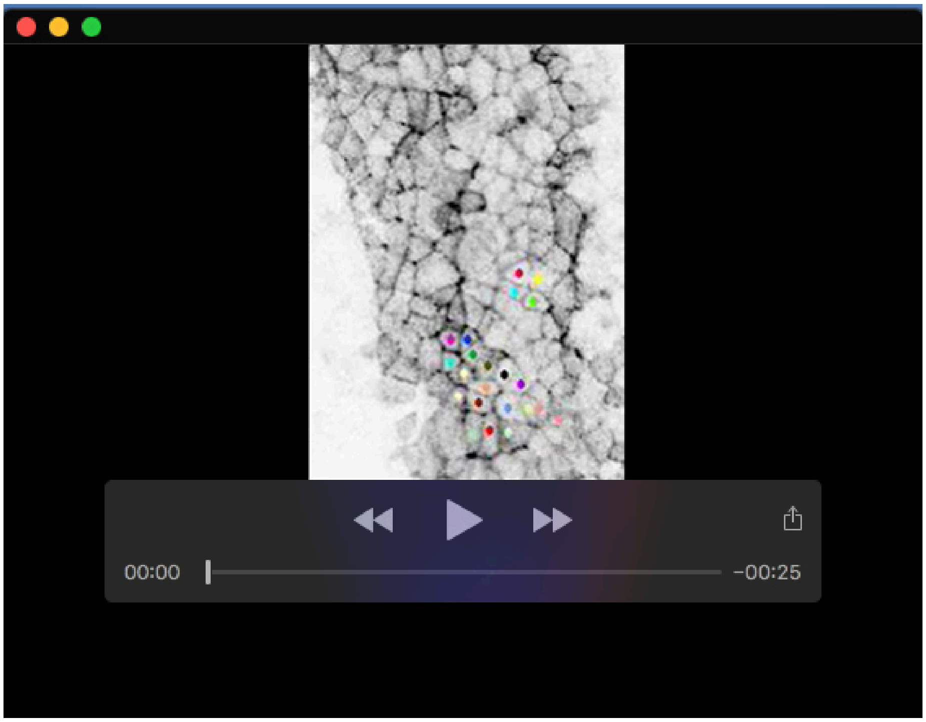


**Movie 17. Prolonged convergent extension at the posterior neural plate of Shroom3 morphant embryos.** Time lapse recording showing a zoomed region (posterior neural plate) of a Shroom3 morphant neural plate from Movie 16. This time lapse recording starts from the time-point during which neural tube closure is normally completed in control embryos. Convergent extension continues at the posterior neural plate of Shroom3 morphant at time points when convergent extension does not take place in control embryos. Single cell tracks showing the collective cell intercalative behaviour (left) and an example of a T1 transition (right). Time interval= 3min

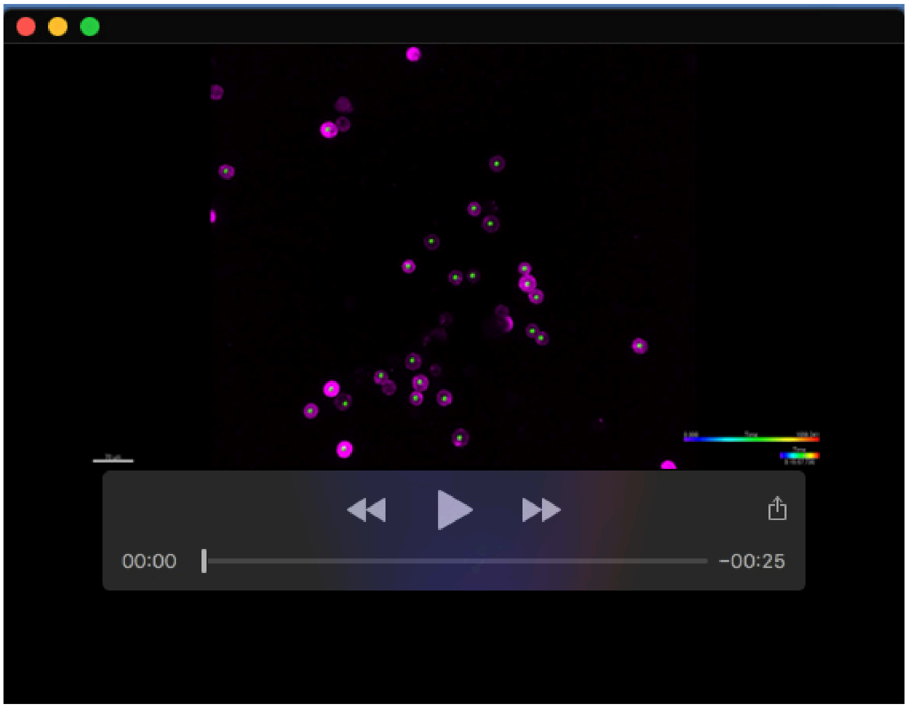


**Movie 18. Assessing the capacity deep surface ectoderm cells to migrate in-vitro.** Time lapse recording of deep surface ectoderm (SE) cells expressing hRFP and plated on a FN coated coverslip.

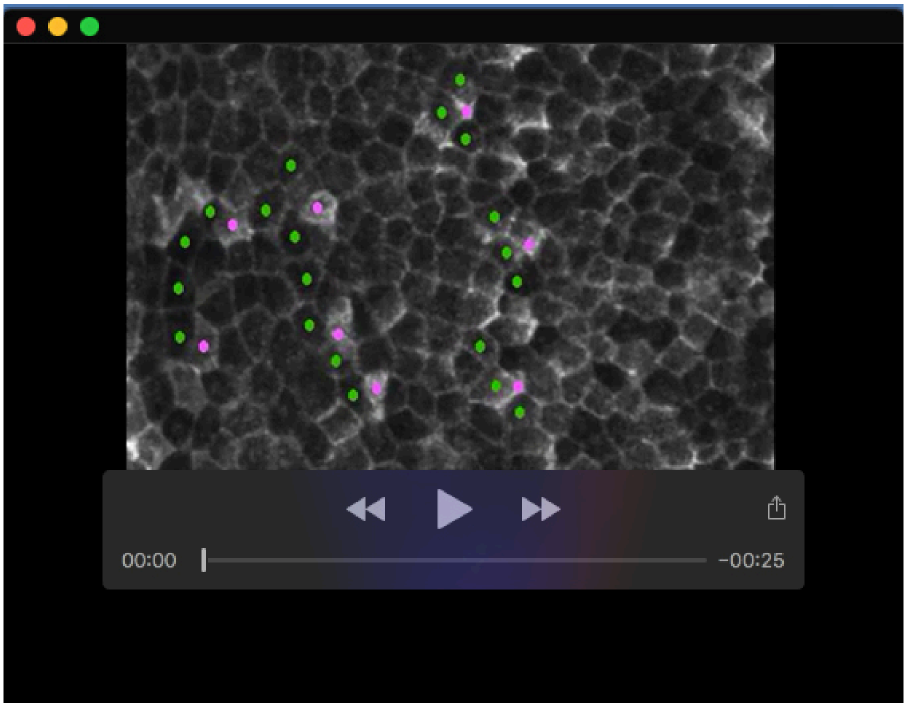


**Movie 19. Superficial and deep surface ectoderm movement during neurulation.** Time lapse recording showing the behaviour of the surface ectoderm during neural tube closure. Surface ectoderm moves towards the medial direction (left). Deep surface ectoderm cells (magenta) never overtake superficial SE cells (green). Time interval = 3 min.

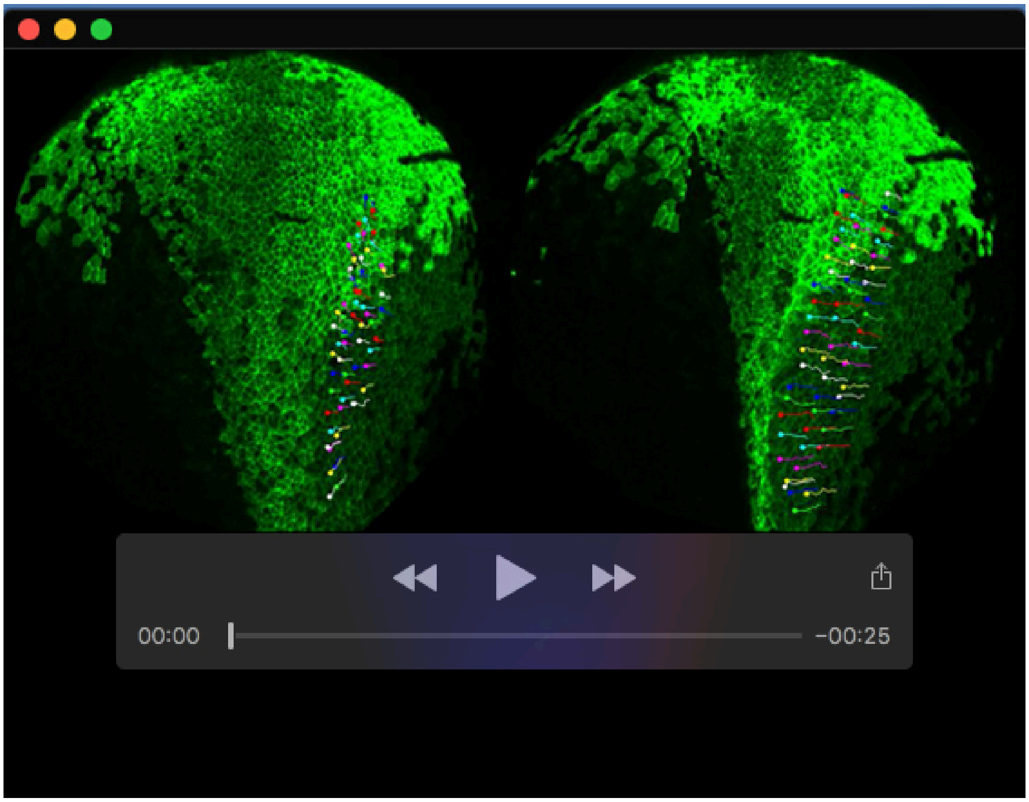


**Movie 20. Apical constriction within the neuroepithelium is correlated in increased movement of surface ectoderm cells.** Time lapse recording of surface ectoderm cell tracking before (left) and after(right) neural plate apical constriction. Time interval= 3 min

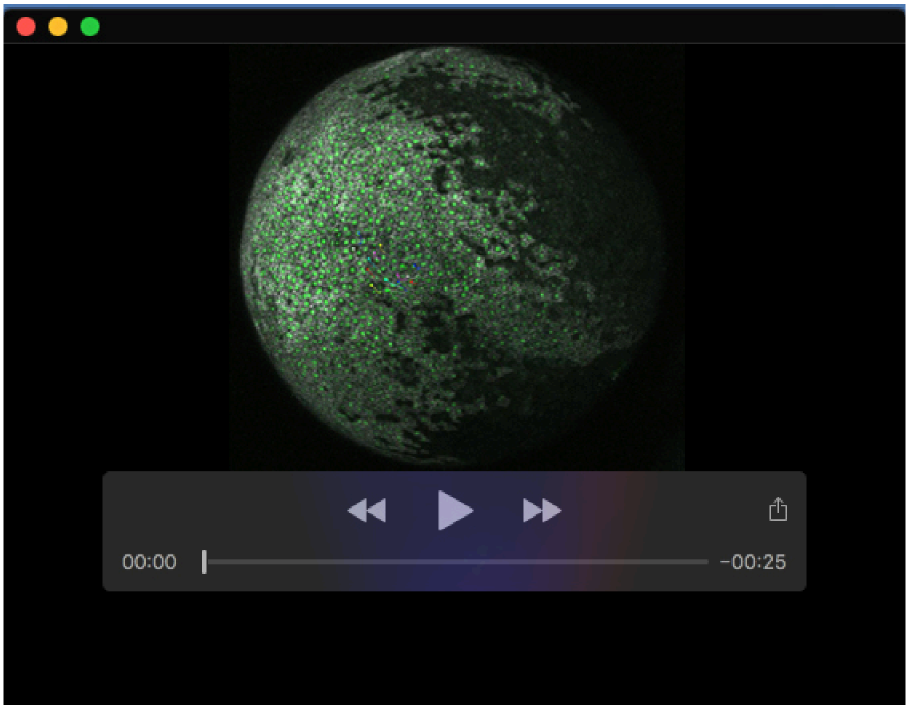


**Movie 21. Single cell tracking of anterior/ventral SE cells during the first phase of neural tube closure**. Anterior/Ventral SE cells move towards the anterior and ventral side of the embryo during neural plate convergent extension. Time interval= 3 min.

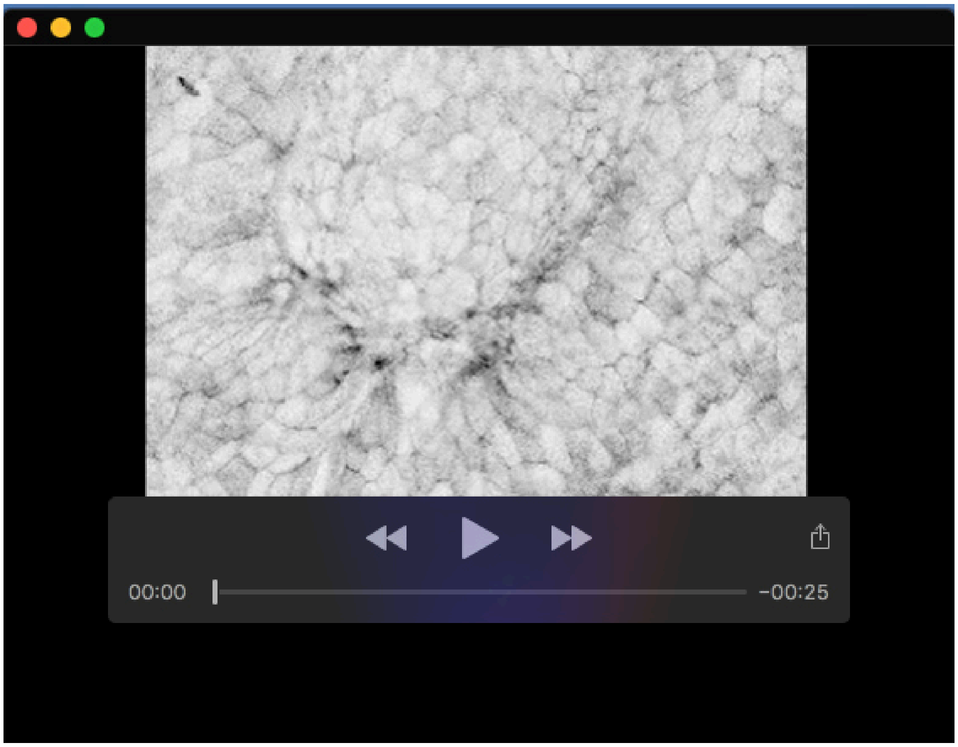


**Movie 22. First example of anterior/ventral SE cell behaviour during the second phase of neural tube closure**. When neural plate apical constriction occurs, anterior/ventral SE cells are stretched towards the dorsal side of the embryo, following the movement of anterior neural plate cells. Time interval= 20 sec.

Development • Supplementary information

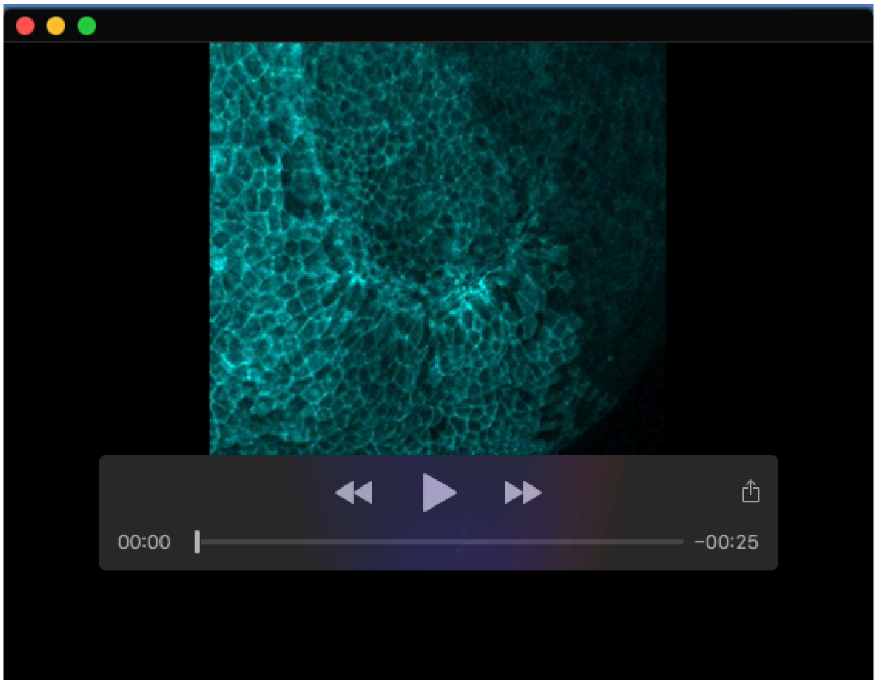


**Movie 23. Second example of anterior/ventral SE cell behaviour during the second phase of neural tube closure.** When neural plate apical constriction occurs, anterior/ventral SE cells are stretched towards the dorsal side of the embryo, following the movement of anterior neural plate cells. Time interval= 150 sec.

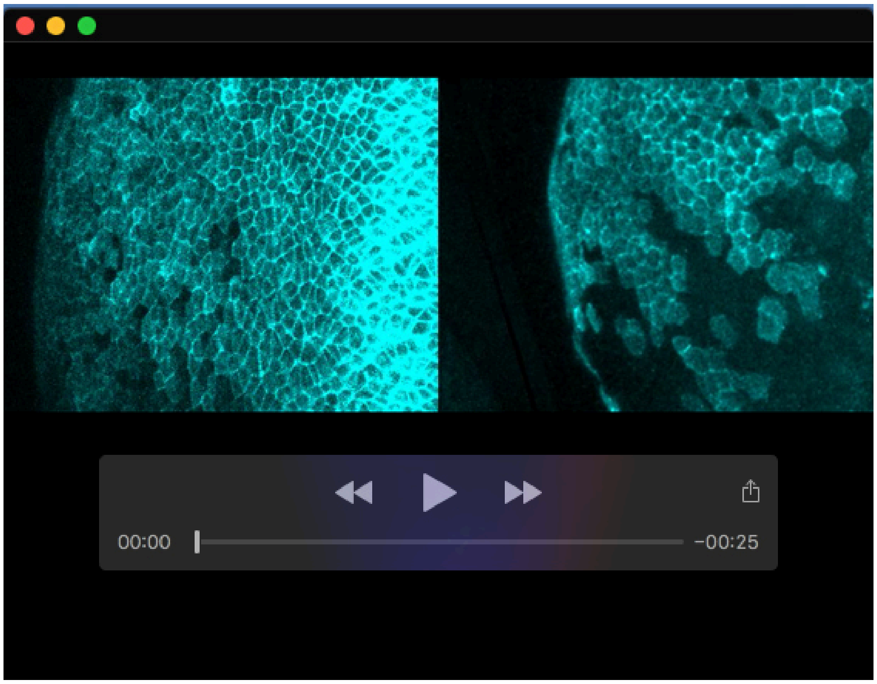


**Movie 24. Neural plate convergent extension is necessary for surface ectoderm medial movement.** Time lapse recording showing the movement of the surface ectoderm in control (left) and Vangl2 morphant embryo (right). Neural plate midline is on the right side. Time interval= 3 min.

Development • Supplementary information

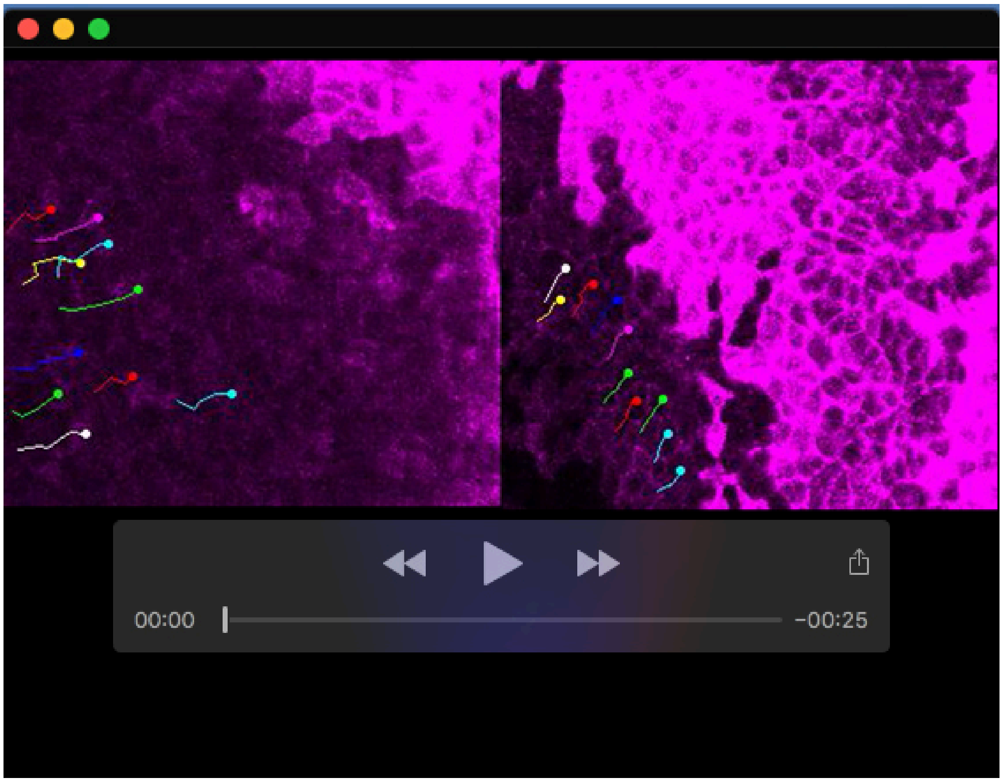


**Movie 25. Neural plate apical constriction is necessary for surface ectoderm medial movement.** Time lapse recording showing the movement of the surface ectoderm in control (left) and Shroom3 morphant embryo (right). Neural plate midline is on the right side. Time interval= 3 min.

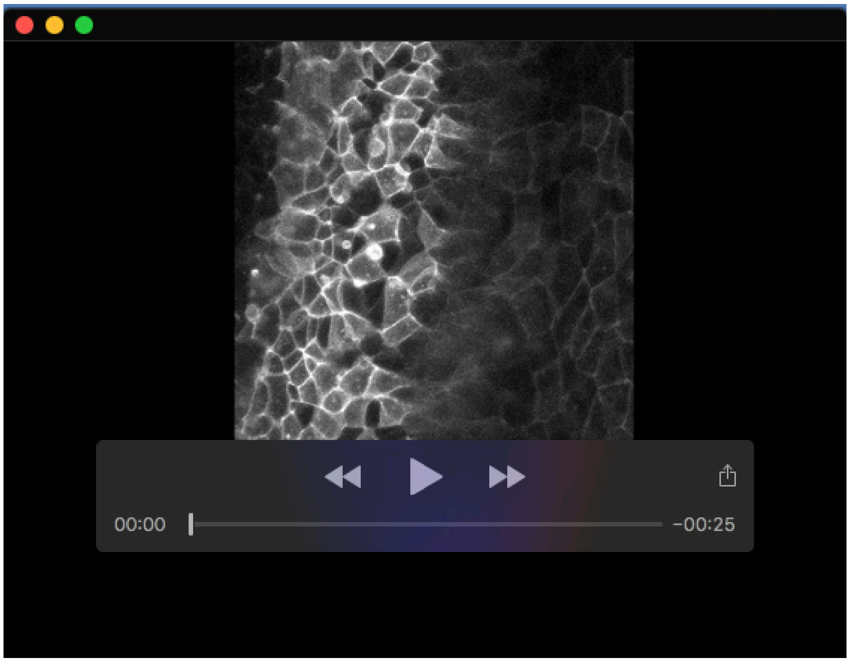


**Movie 26. Neural plate targeted ATP uncaging.** Time lapse recording showing the behaviour of the neuroepithelium (left) and the surface ectoderm (right) before and after ATP uncaging within the neural plate upon UV excitation (bright area).

Development • Supplementary information

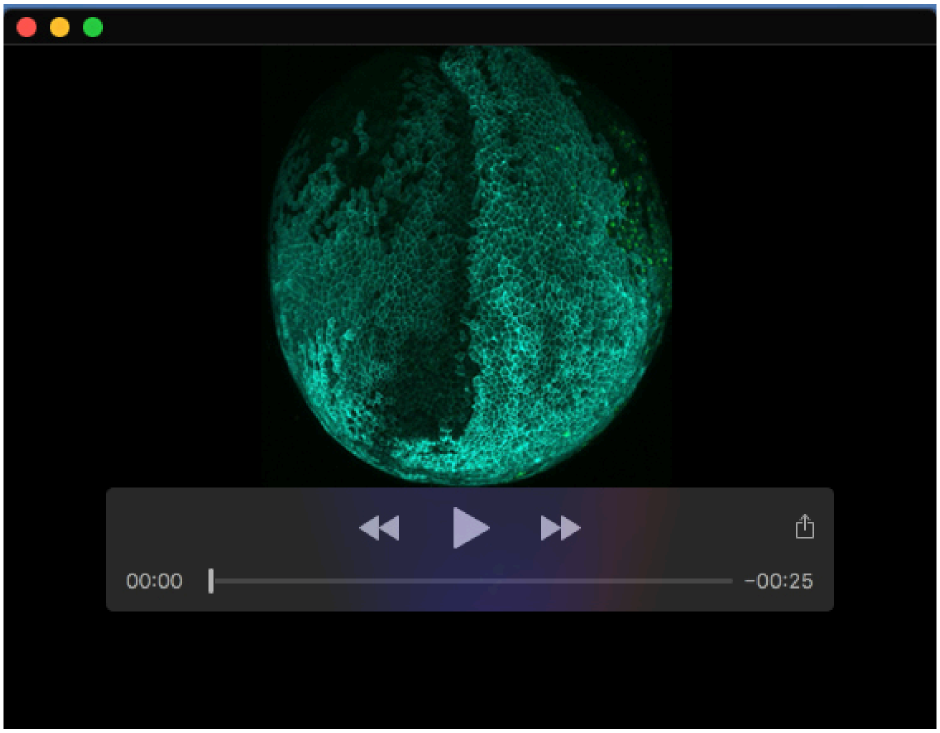


**Movie 27. Surface ectoderm development impacts neural tube closure.** Neural tube close in an embryo with uniliteral targeted surface ectoderm Itgb1 downregulation. Neural tube closure at the neural plate side adjacent to the morphant SE is defective. Red overlay highlights the defective neural tube closure at the affected side. Anterior: bottom, Posterior top. Time interval= 6 min

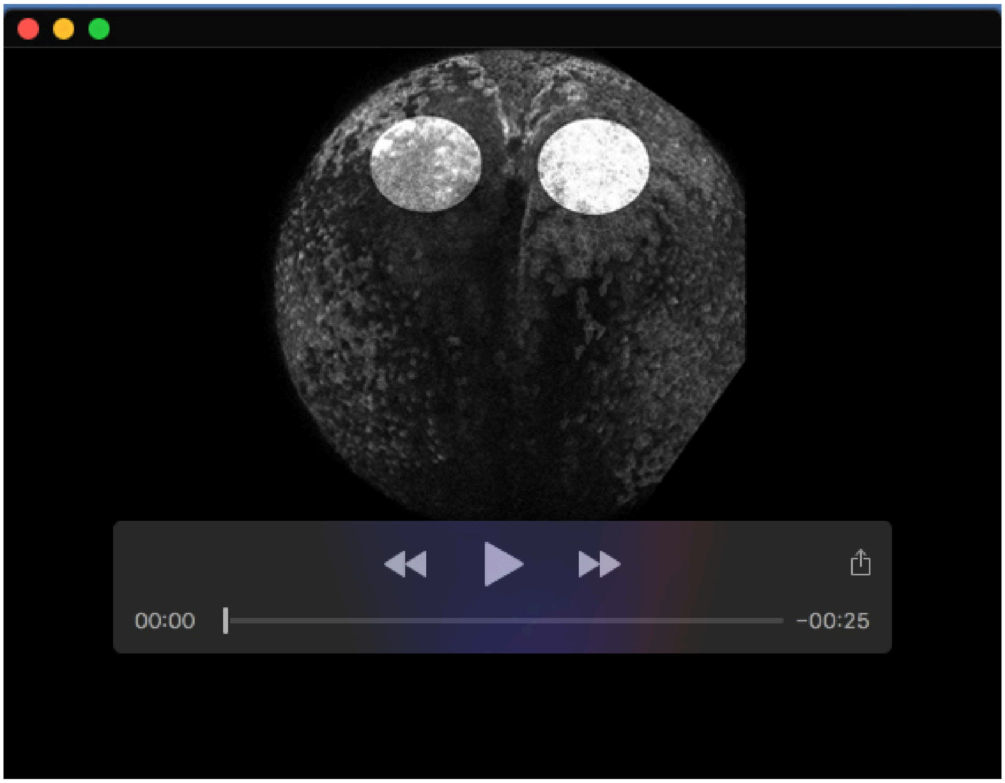


**Movie 28. Surface ectoderm targeted ATP uncaging.** Time lapse recording showing the behaviour of the neuroepithelium (middle) and the surface ectoderm (right and left) before and after ATP uncaging within the surface ectoderm upon UV excitation (bright areas).
