# Supplementary material for: Distinct spatiotemporal contribution of morphogenetic events and mechanical tissue coupling during Xenopus neural tube closure
Source: Development. 2022 Jul 1;149(13):dev200358. doi: 10.1242/dev.200358 (PMC9340557; doi:10.1242/dev.200358)
Supplement: Reviewer comments [file develop-149-200358_review_history.pdf]

Distinct spatiotemporal contribution of morphogenetic events and mechanical tissue coupling during Xenopus neural tube closure

Neophytos Christodoulou and Paris A. Skourides
DOI: 10.1242/dev.200358

Editor: Thomas Lecuit

Review timeline

Original submission:	15 November 2021
Editorial decision:	17 December 2021
First revision received:	31 March 2022
Editorial decision:	10 May 2022
Second revision received:	23 May 2022
Accepted:	25 May 2022

Original submission

First decision letter

MS ID#: DEVELOP/2021/200358

MS TITLE: Distinct spatiotemporal contribution of morphogenetic events and mechanical tissue coupling during Xenopus neural tube closure

AUTHORS: Neophytos Christodoulou and Paris A Skourides

I have now received all the referees' reports on the above manuscript, and have reached a decision. The referees' comments are appended below, or you can access them online: please go to BenchPress and click on the 'Manuscripts with Decisions' queue in the Author Area.

As you will see, the referees express interest in your work, but have some significant criticisms and recommend a substantial revision of your manuscript before we can consider publication. If you are able to revise the manuscript along the lines suggested, which will involve further experiments, I will be happy receive a revised version of the manuscript. Your revised paper will be re-reviewed by one or more of the original referees, and acceptance of your manuscript will depend on your addressing satisfactorily the reviewers' major concerns. Please also note that Development will normally permit only one round of major revision.

We are aware that you may be experiencing disruption to the normal running of your lab that make experimental revisions challenging. If it would be helpful, we encourage you to contact us to discuss your revision in greater detail. Please send us a point-by-point response indicating where you are able to address concerns raised (either experimentally or by changes to the text) and where you will not be able to do so within the normal timeframe of a revision. We will then provide further guidance. Please also note that we are happy to extend revision timeframes as necessary.

Please attend to all of the reviewers' comments and ensure that you clearly highlight all changes made in the revised manuscript. Please avoid using 'Tracked changes' in Word files as these are lost in PDF conversion. I should be grateful if you would also provide a point-by-point response detailing how you have dealt with the points raised by the reviewers in the 'Response to Reviewers' box. If you do not agree with any of their criticisms or suggestions please explain clearly why this is so.

© 2022. Published by The Company of Biologists under the terms of the Creative Commons Attribution License (https://creativecommons.org/licenses/by/4.0/).

Reviewer 1

Advance summary and potential significance to field

The manuscript entitled “Distinct spatiotemporal contribution of morphogenetic events and mechanical tissue coupling during Xenopus neural tube closure” by Christodoulou and Skourides investigates the sequence in which the known cell behaviors necessary for neural tube closure, convergent extension and apical constriction, occur in the anterior and posterior neural plate during neural tube formation. Through live imaging and loss of function approaches the authors conclude that convergent extension precedes apical constriction in the posterior neural plate while the anterior neural plate passively translocates ventroanteriorly, before going through apical constriction and neural tube closure. The study also shows that the non-neural ectoderm moves passively following the forces that the neighbor neural plate exerts while folding, and that an aberrant increase in the tension generated in non-neural ectodermal cells opposes to the neural plate cell behaviors of convergent extension and apical constriction.

This study contributes to the understanding of the cellular events necessary for neural tube formation which may be relevant to understanding the occurrence of neural tube defects the devising of potential therapeutics for preventing them.

Comments for the author

The manuscript entitled “Distinct spatiotemporal contribution of morphogenetic events and mechanical tissue coupling during Xenopus neural tube closure” by Christodoulou and Skourides investigates the sequence in which the known cell behaviors necessary for neural tube closure, convergent extension and apical constriction, occur in the anterior and posterior neural plate during neural tube formation. Through live imaging and loss of function approaches the authors conclude that convergent extension precedes apical constriction in the posterior neural plate while the anterior neural plate passively translocates ventroanteriorly, before going through apical constriction and neural tube closure. The study also shows that the non-neural ectoderm moves passively following the forces that the neighbor neural plate exerts while folding, and that an aberrant increase in the tension generated in non-neural ectodermal cells opposes to the neural plate cell behaviors of convergent extension and apical constriction.
This study contributes to the understanding of the cellular events necessary for neural tube formation which may be relevant to understanding the occurrence of neural tube defects the devising of potential therapeutics for preventing them.
Overall, the manuscript is clearly written, and data are well presented and analyzed appropriately. The knowledge gained is, however, only incremental. Convergent extension and apical constriction have long been recognized as crucial cellular events necessary for neural tube closure and this study is only contributing with assigning these cell behaviors to anterior and posterior neural plate. Similarly, the passive role of non-neural ectoderm has been reported before. Nevertheless, the study adds to the understanding of neurulation by demonstrating that increasing the tension of this tissue does interfere with the neural plate-autonomous morphogenesis. One major issue that the authors need to address is the potential limitations to the approach used to target neural or non-neural ectoderm which may not be as specific as needed for this type of study.

Major issues:
1) More convincing evidence is needed to demonstrate the specificity of the manipulations of gene expression in neural versus non-neural ectoderm. This requires labeling of the two tissues with specific markers, like Sox2 and E-cadherin, respectively. Moreover, I do not think injecting a 4-cell stage embryo allows for such exclusivity in targeted cells contributing only to neural or non-neural ectoderm and there are differences between posterior and anterior contributions from these blastomeres that may prevent concluding on the differential cell behaviors and participation of these regions of tissue during neurulation.
2) The behavior of cells dissociated and allowed to attach to a substrate in a dish is not indicative of the behavior of those cells in vivo. The authors need to refrain from concluding that the data from culturing deep SE cells indicates that “deep SE cells don’t acquire a migratory phenotype during neurulation”.
3) The authors need to indicate the spatial resolution of the ATP uncaging to be able to interpret the results as truly specific to this manipulation.

© 2022. Published by The Company of Biologists under the terms of the Creative Commons Attribution License (https://creativecommons.org/licenses/by/4.0/).

Minor issues:
4) Some sentences are confusing and require revision: lines 223-225; lines 413-416.
5) The reporting of angles of cell division is incorrect. "," should be replaced by "." and the relevant/significant figures to be reported are determined by the error of the data, which given that it is within the unit of the grade, reporting anything beyond the unit is inappropriate. Thus, the angles should be 49±2 for both samples.
6) In Figure 5, panel E' should be labeled as G'.
7) In Figure 6, panels J-N need to be reordered to fit better with the order of presentation of results in the text.
8) In Supplementary Figure 2, it seems that the labels (red and green double arrows) for 165 and 225 min in panels C and D should be swapped.
9) In Supplementary Figure 4C, label appears to be histone-GFP, not mem-GFP as indicated.

Reviewer 2

Advance summary and potential significance to field

The paper provides excellent, high resolution time-lapse images of Xenopus neural tube closure and uses the data to address several outstanding questions in vertebrate neural tube closure. This is clearly an important topic given that neural tube closure is fundamental to the development of the CNS and is the root of prominent human birth defects.

The paper provides several notable insights:

1. Methodologically, the paper provides a modern paradigm for the kind of large-scale integration of tissue and cell movements that is required to understand morphogenesis. This has been achieved to date largely in Drosophila tissues so development of such methods for vertebrate NTC is a welcome addition.

2. The anterior and posterior neural plate are known to engage in distinct morphogenetic processes, with apical constriction dominating anteriorly and cell intercalation (convergent extension) dominating posteriorly.

3. The paper does an excellent job of clarifying the role of epidermal ectoderm in neural tube closure. Using careful analysis of normal NTC as well as a clever use of caged ATP to acutely drive local contractions, the authors make a very strong argument against any contribution to NTC from mechanical pushing by epidermis.

4. Instead, by manipulating cell/matrix interactions, they show that radial interaction is required to release tension in the epidermal ectoderm in order for NTC to proceed normally. Thus, balanced tension between neural and non-neural ectoderm is the key.

However, this comprehensive and useful dataset is very sloppily presented. This paper represents a large body of work, and I very much hope the authors will make an effort to improve it so that it will be suitable for publication.

Comments for the author

Major revisions:
1. In Fig. 3C, D the authors describe "anterior movement" of the anterior neural plate. But it is not clear what this movement is relative to. From the traces, it would seem this movement is relative simply to the fixed frame of the movie, which might result from the entire embryo drifting. I think the result here is important, so wouldn't a better metric be to quantify the movement of anterior neural plate cell to posterior neural plate cells? (i.e. using a small number of posterior cells as fiducial markers). This should be very simple to do and would make this result compelling.

2. I am not convinced that AC strictly follows CE, and the claim (line 245) that AC is not spatially restricted seem unsupported. The figure 4D makes this seem likely posteriorly but not anteriorly. Since CE does not occur anteriorly it would make more sense to treat the two separately anyway.

© 2022. Published by The Company of Biologists under the terms of the Creative Commons Attribution License (https://creativecommons.org/licenses/by/4.0/).

Being more circumspect in the writing will serve the authors well in the long run. I also think the statement about “sole contributor” should be removed from line 234.
Very hard to prove a negative.
3. None of the data concerning shroom3 in Fig. 5 or Supp. Fig 2 are quantified.
Numbers and statistics backing the claims must be presented or the data removed.
Either route is acceptable, as the shroom3 data here are not crucial to the overall story.
4. Likewise, the data in Fig. 4K must be explained, as the current presentation leaves them uninterpretable. No specifics are given for what the arrows or the colors in this chart represent, or what the upper and lower panels show. The same is true for Fig. 6H. In addition, no statistics or N numbers are provided for either figure. Are these PIV from a single sample, or do these somehow represent multiple samples?

Making these data 1) convincing and 2) understandable is central to the paper, as the authors are concluding precisely the opposite of a previous paper (Morita, 2012). They need to get this right.
5. the data for Fig. 6N, showing planar polarization is maintained must be quantified. The images are not sufficient.

Minor comments:
1. Anterior in three different orientations in Fig 1 alone (top, bottom or left depending on the panel? The reader will be helped by picking one orientation and sticking with it for the whole paper. (Another example: Fig. 2A shows Anterior up and Posterior down within the panels, yet the posterior panel is on top.). I am sorry to be picky, but this is a very complex paper and your readers will thank you if you make it easier!
2. Keller, Shih, and Sater 1992 (PMID: 1600240) must be cited in discussion of Figure 1B (line 144) and line 180, as this precise pattern of movement was reported in that paper. Probably should cite that work in the Intro as well.
3. The method of quantification for Fig. 2C should be explicitly stated. If I read it right, the values are the ratio of AP and ML junctions, but that is never stated and how AP and ML are decided is not reported. Ditto for Fig. 3B.
4. Line 199. I am not sure the word “stems” is appropriate here as it implies cause, but only correlation is shown.
5. I do not understand the schematics at the right of fig. 4A. How is “+/- AC” “or +/-CE” determined? Is this subjective? What is this meant to convey?
Also, it seems that the shape of the tissue is being used to assess whether CE or AC is active. This is not a convincing argument, only cell-level quantification such as that in panel D or G is acceptable. Maybe best to just remove this analysis? Or explain it adequately.
5. I think it’s crucial to explicitly state where the data from Fig. 4E-H are taken from, anterior or posterior? Also the explanation for Panel D seems to be missing from the fig. 4 Legend. Please proofread the legend.

Reviewer 3

Advance summary and potential significance to field

The manuscript by Christodoulou and Skourides describes the morphogenetic movements that drive neural tube closure (NTC) in the vertebrate model Xenopus laevis. In multiple model organisms, including Xenopus, NTC involves two main morphogenetic processes – convergent extension, which switches the long axis of the neural plate from mediolateral to anteroposterior, and apical constriction.
Here, the authors define two distinct phases of NTC. The first phase involves convergent extension but not apical constriction. Moreover, convergent extension during phase I is restricted to the posterior/caudal region of the neural plate.
On the other hand, in phase II, cells throughout the neural plate undergo apical constriction synchronously to fold and close the neural tube.

In this manuscript, Christodoulou and Skourides have drawn two main conclusions.
First, that the neural plate elongation along the anteroposterior axis, which is caused by convergent extension movements in the caudal region during phase I positions the rostral part of the neural plate in its final, anterior position.

© 2022. Published by The Company of Biologists under the terms of the Creative Commons Attribution License (https://creativecommons.org/licenses/by/4.0/).

Second, they have shown that a balance between the mechanical properties of the developing neural plate and that of the adjoining surface ectoderm is necessary for proper neural tube closure. More specifically, they demonstrate that the morphogenetic movements within the neural plate pull the surface ectoderm medially, and that optogenetically stiffening the surface ectoderm abrogates movements in the neural tube. Thus, they present evidence to contradict previous reports where it has been suggested that the surface ectoderm pushes the developing neural plate medially.

Comments for the author

This is an interesting study, which also significantly advances our understanding of the mechanical underpinnings of vertebrate neural tube closure and will be a useful addition to the field. However, for the manuscript to be considered suitable for publication in Development, the authors should address the following comments.

Major comments

1. One major conclusion the authors present in this manuscript is that the anterior movement of the rostral neural plate is passive and that it occurs as convergent extension of the caudal neural plate pushes it toward the anterior.

This conclusion is mainly based on the analysis of Vangl2 MO embryos. Embryos with a downregulation of Vangl2, which lack caudal convergent extension movements, show limited anterior movement of the rostral neural plate suggesting that the rostral movement is passive. However, the authors do not account for the possibility that a homogenous knockdown of Vangl2 may have other effects. For instance, could Vangl2 be directly required within the rostral neural plate to promote its active anterior movement? The authors postulated that if the rostral cells actively move anteriorly, and convergent extension in the caudal region is permissive for this movement, then the anteroposterior junctions of the cells at the boundary between the rostral and the caudal regions should elongate in Vangl2 MO embryos. However, the authors did not find this to be true, therefore further strengthening their conclusion. However could the boundary cells in Vangl2 MO be stiff/tensed, thereby resisting changes in geometry despite experiencing a pulling force from the rostral cells? How do these cells look like in wild type embryos? If the anterior movement of the rostral region was inhibited (example, by introducing a physical barrier), would the boundary cells in wild type begin to exhibit signs of compression due to the anterior movement of the caudal region? Alternatively, in explants, if the rostral neural plate is replaced by a gel, would the gel also move anteriorly?

As another option, could rostral neural plate cells, explanted and devoid of caudal cells, migrate?

2. Cells in the neural plate in Shroom3 MO embryos do not undergo apical constriction, a behavior that is exhibited by all cells in the neural plate in wild type. Yet defects in neural tube closure are only restricted to the rostral region. The authors explain this by showing that caudal convergent extension movements continue in Shroom3 MO embryos till significantly later than in wild type. They suggest that this prolongation of convergent extension compensates for the lack of apical constriction. However, it is unclear if, overall, in Shroom3 MO, there is more convergent extension or just a reduction in its rate.

For instance, do the same number of neighbor exchange events occur per hour in wild type and Shroom3 MO? For convergent extension to compensate for the lack of apical constriction, one would expect there to be an overall increase in (and not just a delay in the completion of) convergent extension. Furthermore, is development overall slower in Shroom3 MO? In other words, are 60 min for wild type also equal to 60 min for Shroom3 MO?

3. In fig. 5, the authors show that explanted surface ectoderm cells do not show migratory behavior. Based on this, they argue that surface ectoderm cells do not have the capacity to migrate during neurulation. However, could it be that important factors required for ectoderm cell migration are missing in culture? I am not sure what can be used here as a positive control but perhaps one may be required. Alternatively, on lines 296 and 297, the authors conclusion should be qualified with this caveat.

Furthermore, the authors also present evidence to suggest that embryos lacking morphogenetic movements within the neural plate show an absence of movement of the surface ectoderm. Based on this, they conclude that surface ectoderm movements are passive, and a result of mechanical coupling with the moving cells of the neural plate. It may be useful to at least discuss the possibility that the lack of free space in such embryos (as neural plate cells do not move forward) could be the reason for the lack of ectoderm movements.

© 2022. Published by The Company of Biologists under the terms of the Creative Commons Attribution License (https://creativecommons.org/licenses/by/4.0/).

While it is true that the authors have shown later that optogenetically generated pulling forces within the neural plate can pull the surface ectoderm cells (and vice versa), those experiments only show that such pulling forces are sufficient to achieve ectoderm movement, and not that they are necessary for the same. This should be made clear in the main text.
Minor comments Line 139: Histone-RFP should instead be Histone-GFP, as it is in fig. 1B. In general there are a few errors in the text and the figures. Some of them have been mentioned below. The manuscript requires thorough proofreading.
Line 143: How were neuroepithelial cells distinguished from surface ectoderm cells based on uniform H2B labelling? Was this done based on their morphological features? This information should be briefly included.
Lines 157 and 158: The cell division angle data originates from only two embryos. A couple more embryos should be examined to determine if the observations are consistent. In addition, the values mentioned in these lines use a comma for a decimal, which is inconsistent with the way values have been represented in the rest of the manuscript.
Line 159: To support the claim that cell proliferation rates in the rostral and the caudal regions of the neural plate are similar, the authors have only shown two images, one each from early and late stage of development. Perhaps a graph summarizing the population data would be necessary to include.
Line 225: To determine if the rostral cells pull the caudal cells, the authors have analyzed the geometry of cells at the boundary between the two regions. How were the boundary cells defined, considering that there is no clear demarcation (rather just a gradual narrowing) between the two regions?
Lines 344-346: Information with respect to the sample size and the population data are missing. Were any paired statistical analyses performed at the population level to determine if the effect of ATP uncaging within the neural plate on the surface ectoderm was consistent and can be considered significant?
Line 381: ‘Anteroposterior’ should actually be mediolateral, as the authors have examined the shrinkage of junctions along the mediolateral axis. That would then also be consistent with the data represented in Fig. 6J, L, and M.
Figures Across all figures, it would be best to use the same embryo orientation to avoid confusion. For example: the orientations of the embryo in figs. 1B and 1D are opposite to each other.
Fig. 1E: Column headings (Rostral vs Caudal) are switched.
Fig. 2A: Labels for the double-headed arrows are switched in the top row (0 min vs 60 min).
Fig. S2C, last image: the time labels for the double-headed arrows (165 min vs 225 min are switched).
Fig. 3C: Embryo orientation should be indicated.
Fig. 5G: Labels for the anteroposterior labelling are cropped.
Fig. 5K: Labels for images, similar to that for fig. 6K, are missing.
In general, quantifications and detailed information regarding the sample size and statistics should be included for all experiments wherever possible. For instance, in fig. 2C, where three timepoints for each group have been compared it is assumed that a multiple comparisons correction must have been made.
However, the corresponding information (for example, which correction test was applied) is missing.

First revision

<u>Author response to reviewers' comments</u>

Reviewer 1 Comments for the Author...

The manuscript entitled “Distinct spatiotemporal contribution of morphogenetic events and mechanical tissue coupling during Xenopus neural tube closure” by Christodoulou and Skourides investigates the sequence in which the known cell behaviors necessary for neural tube closure, convergent extension and apical constriction, occur in the anterior and posterior neural plate during neural tube formation. Through live imaging and loss of function approaches the authors conclude that convergent extension precedes apical constriction in the posterior neural plate while

© 2022. Published by The Company of Biologists under the terms of the Creative Commons Attribution License (https://creativecommons.org/licenses/by/4.0/).

the anterior neural plate passively translocates ventroanteriorly, before going through apical constriction and neural tube closure. The study also shows that the non-neural ectoderm moves passively following the forces that the neighbor neural plate exerts while folding, and that an aberrant increase in the tension generated in non-neural ectodermal cells opposes to the neural plate cell behaviors of convergent extension and apical constriction.

This study contributes to the understanding of the cellular events necessary for neural tube formation, which may be relevant to understanding the occurrence of neural tube defects the devising of potential therapeutics for preventing them.

Overall, the manuscript is clearly written, and data are well presented and analyzed appropriately. The knowledge gained is, however, only incremental. Convergent extension and apical constriction have long been recognized as crucial cellular events necessary for neural tube closure and this study is only contributing with assigning these cell behaviors to anterior and posterior neural plate. Similarly, the passive role of non-neural ectoderm has been reported before. Nevertheless, the study adds to the understanding of neurulation by demonstrating that increasing the tension of this tissue does interfere with the neural plate-autonomous morphogenesis. One major issue that the authors need to address is the potential limitations to the approach used to target neural or non-neural ectoderm which may not be as specific as needed for this type of study.

We would like to thank the Reviewer for their useful feedback and corrections. We respectfully disagree with the reviewer's comment that the knowledge gained by our study is only incremental especially given the importance of the spatial and temporal aspects and coordination of these events to drive NTC, but we appreciate that the Reviewer acknowledges the contribution of our study to the understanding of neurulation.

Major issues:
1) More convincing evidence is needed to demonstrate the specificity of the manipulations of gene expression in neural versus non-neural ectoderm. This requires labelling of the two tissues with specific markers, like Sox2 and E-cadherin, respectively. Moreover, I do not think injecting a 4-cell stage embryo allows for such exclusivity in targeted cells contributing only to neural or non-neural ectoderm and there are differences between posterior and anterior contributions from these blastomeres that may prevent concluding on the differential cell behaviors and participation of these regions of tissue during neurulation.
Injection of dorsal and ventral blastomeres at the 4-cell stage has been used in the majority of studies using Xenopus to study gene function within the neural plate or the surface ectoderm respectively. When injecting the dorsal or ventral blastomeres to target the neuroepithelium or the surface ectoderm we always inject near the surface of the apical membrane of the blastomere to avoid targeting of mesodermal tissues. To directly address the reviewer's comment however, we injected dorsal blastomeres and ventral blastomeres at the 4-cell stage and stained embryos using an anti-Sox3 (neural marker) antibody. This showed that targeting a dorsal blastomere at the 4-cell stage leads to neural plate targeting (Supplementary Figure 2 A-B) and injection of a ventral blastomere leads to surface ectoderm targeting (Supplementary Figure 9A-B). Furthermore, we quantified targeting of the neural plate, the surface ectoderm and mesodermal tissues after targeted injections at 1 dorsal or ventral blastomere at the 4-cell stage in cross sections of neurula stage embryos. This revealed that our targeted dorsal blastomere injections led mainly to neural plate targeting (Supplementary Figure 2 C) and ventral injections led mainly to surface ectoderm targeting (Supplementary Figure 9C).

© 2022. Published by The Company of Biologists under the terms of the Creative Commons Attribution License (https://creativecommons.org/licenses/by/4.0/).

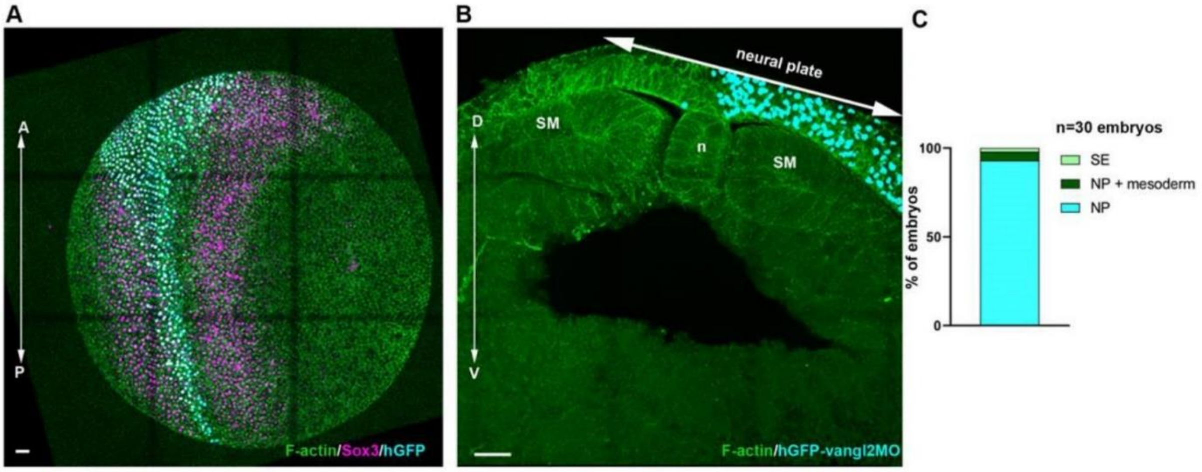

Figure S2.

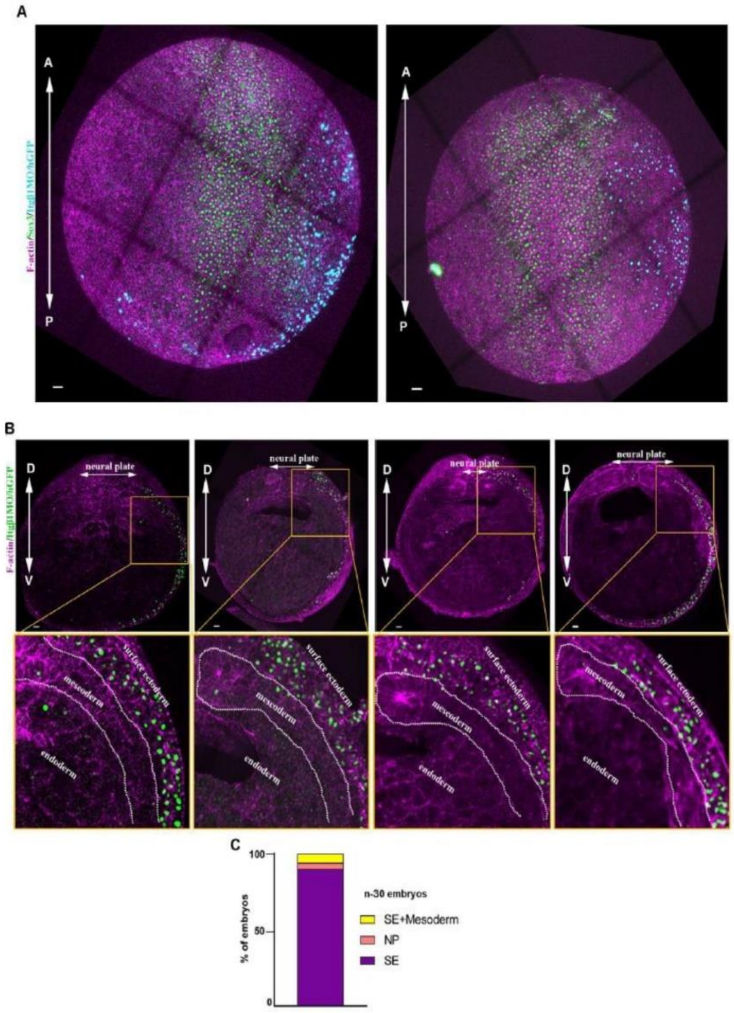

2) The behavior of cells dissociated and allowed to attach to a substrate in a dish is not indicative of the behavior of those cells in vivo. The authors need to refrain from concluding that the data from culturing deep SE cells indicates that “deep SE cells don’t acquire a migratory phenotype during neurulation”.

We have revised the manuscript according to the reviewer's suggestion. We now state that “This revealed that deep SE cells, unlike other migratory cell types, do not possess the capacity to migrate when cultured ex-vivo on a FN substrate when explanted”

© 2022. Published by The Company of Biologists under the terms of the Creative Commons Attribution License (https://creativecommons.org/licenses/by/4.0/).

3) The authors need to indicate the spatial resolution of the ATP uncaging to be able to interpret the results as truly specific to this manipulation.

In order to address the reviewer's comment, we injected embryos with the photoconvertible protein mEOS and subsequently photoconverted mEOS using UV excitation (as we have used for ATP unchanging). This shows that UV photoconversion can be spatially controlled in our system.

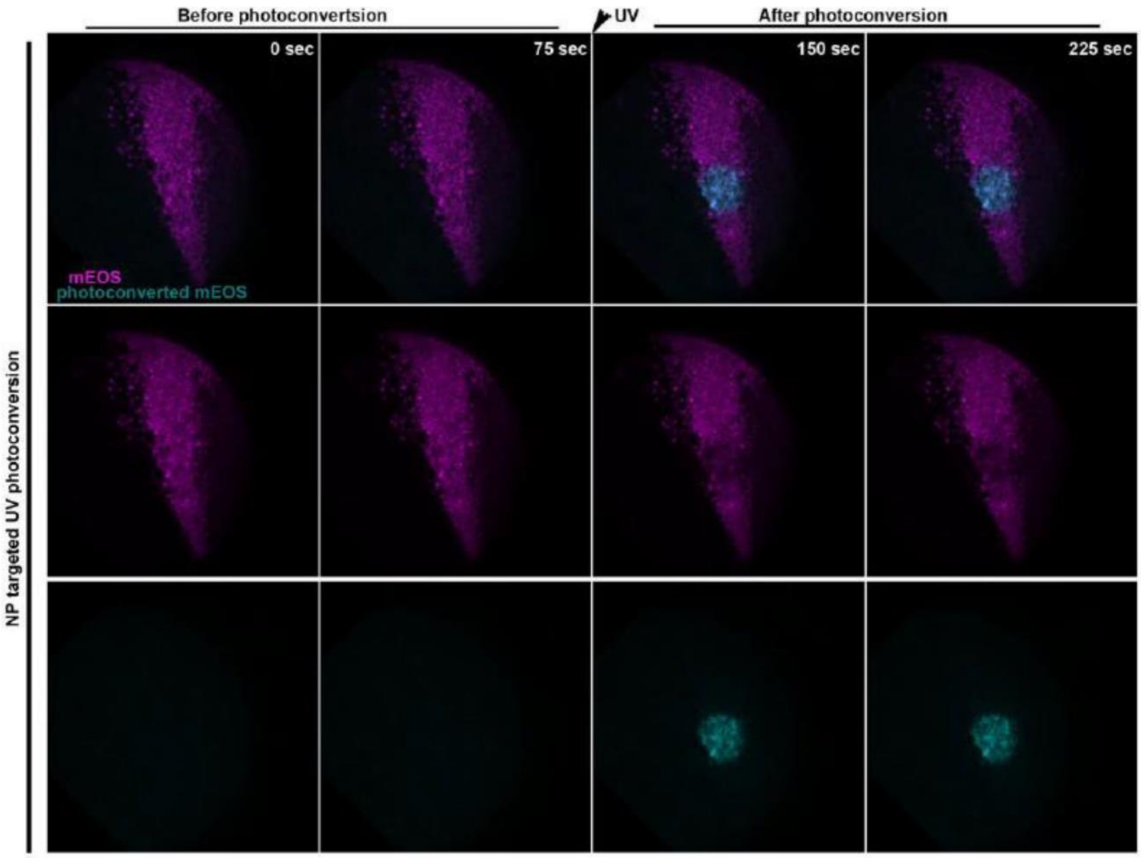

© 2022. Published by The Company of Biologists under the terms of the Creative Commons Attribution License (https://creativecommons.org/licenses/by/4.0/).

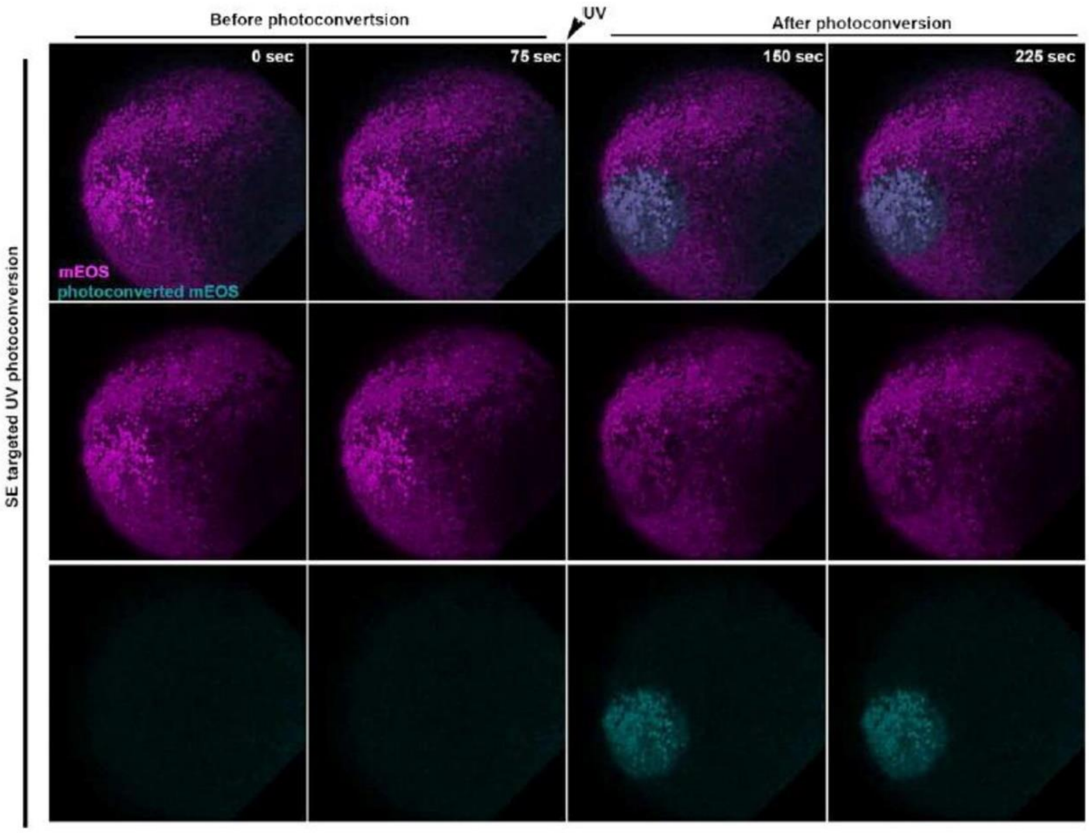

Additionally in supplementary Figure 1 we show that UV excitation can photoconvert mEOS at distinct regions within the neural plate highlighting the spatial control of UV excitation.

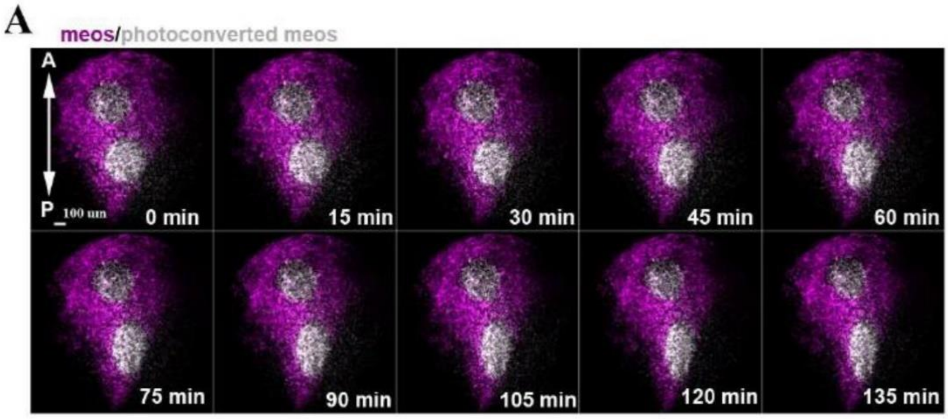

Figure S1

Minor issues:
4) Some sentences are confusing and require revision: lines 223-225; lines 413-416.
These sentences have been revised accordingly.

5) The reporting of angles of cell division is incorrect. “,” should be replaced by “.” and the relevant/significant figures to be reported are determined by the error of the data, which given that it is within the unit of the grade, reporting anything beyond the unit is inappropriate. Thus, the angles should be 49±2 for both samples.

According to the reviewer’s comment we are now reporting the angle values and the error within the unit of the grade. According to the suggestion from Reviewer3 we have now quantified cell division angles from 5 different embryos.

© 2022. Published by The Company of Biologists under the terms of the Creative Commons Attribution License (https://creativecommons.org/licenses/by/4.0/).

6) In Figure 5, panel E' should be labeled as G'.
This has now been corrected.

7) In Figure 6, panels J-N need to be reordered to fit better with the order of presentation of results in the text.
Figure 6, panels J-N are now presented in Figure 9 and are ordered according to the presentation of the results in the text.

8) In Supplementary Figure 2, it seems that the labels (red and green double arrows) for 165 and 225 min in panels C and D should be swapped.
This has been revised and presented in supplementary Figure 6.

9) In Supplementary Figure 4C, label appears to be histone-GFP, not mem-GFP as indicated.

We have replaced histone-GFP with mem-GFP. Supplementary Figure 4 is now Supplementary Figure 10.

Reviewer 2 Comments for the Author...

We would like to thank the reviewer for the feedback that allowed us to improve our manuscript.

Major revisions:

1. In Fig. 3C, D the authors describe "anterior movement" of the anterior neural plate. But it is not clear what this movement is relative to. From the traces, it would seem this movement is relative simply to the fixed frame of the movie, which might result from the entire embryo drifting. I think the result here is important, so wouldn't a better metric be to quantify the movement of anterior neural plate cell to posterior neural plate cells? (i.e. using a small number of posterior cells as fiducial markers). This should be very simple to do and would make this result compelling.

We are confident that the anterior movement of the neural plate is not due to embryo drifting since this movement is only present at the anterior part of the neural plate. This is evident by single cell tracking (compare anterior and posterior NP cell movement in Fig 1F-G. Furthermore, according to the Reviewer's suggestion, we tracked the movement of anterior and posterior NP cells in the same embryo (Supplementary Figure 3). Quantification of cell displacement vector angle shows that anterior neural plate cells move towards more anterior regions in trajectories parallel with the anteroposterior embryo axis while the posterior neural plate cell movement is perpendicular to the same axis.

© 2022. Published by The Company of Biologists under the terms of the Creative Commons Attribution License (https://creativecommons.org/licenses/by/4.0/).

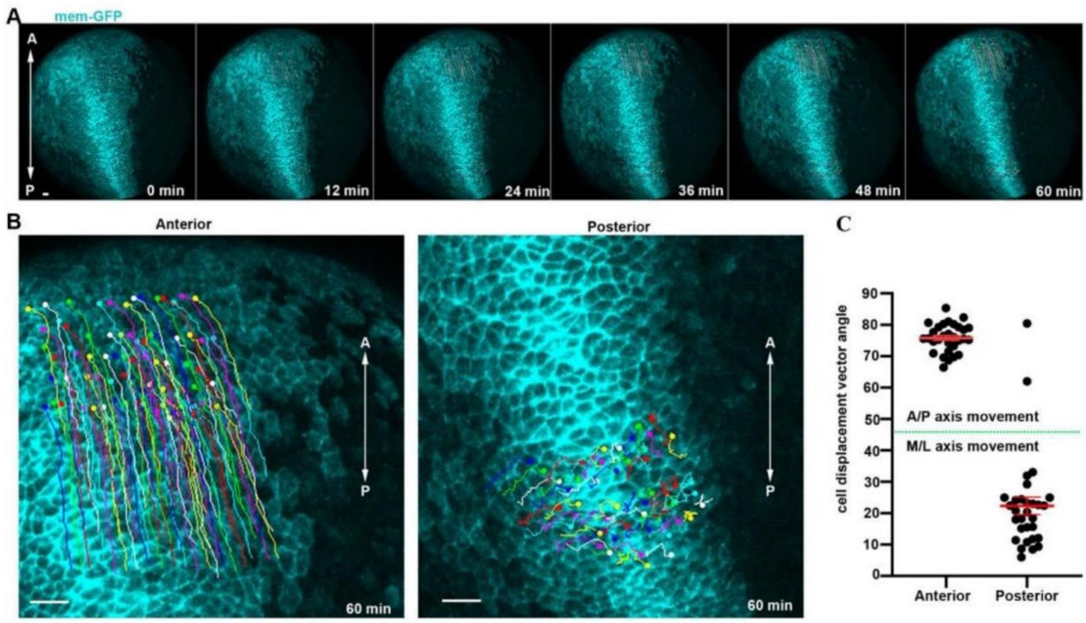

Figure S3

Additionally following tissue deformation of the posterior and anterior neural plate over time using photoconverted mEOS again reveals that anterior directed movement is only present in the anterior region of the embryo.

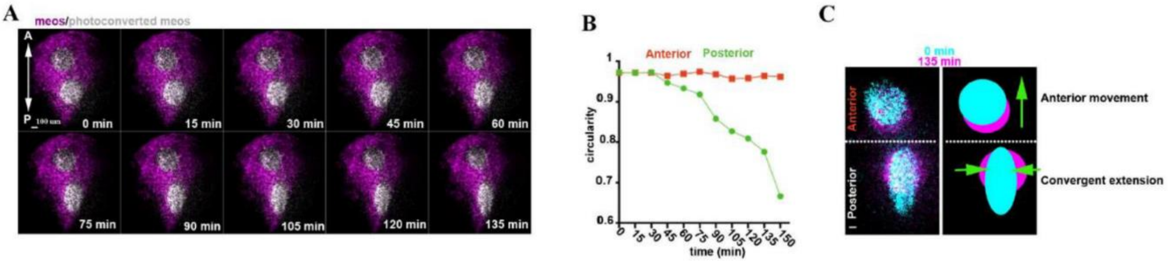

Figure S1

2. I am not convinced that AC strictly follows CE, and the claim (line 245) that AC is not spatially restricted seem unsupported. The figure 4D makes this seem likely posteriorly but not anteriorly. Since CE does not occur anteriorly, it would make more sense to treat the two separately anyway. Being more circumspect in the writing will serve the authors well in the long run.

We agree with the Reviewer that AC does not strictly follows CE. We do not argue that termination of CE is necessary for AC. Our data show that AC and CE don't have temporal overlap. We have now revised the manuscript to make clear that AC and CE do not have temporal overlap.

I also think the statement about "sole contributor" should be removed from line 234. Very hard to prove a negative.

We have replaced the word "sole" with "major" in the revised manuscript.

3. None of the data concerning shroom3 in Fig. 5 or Supp. Fig 2 are quantified. Numbers and statistics backing the claims must be presented or the data removed. Either route is acceptable, as the Shroom3 data here are not crucial to the overall story.
In the revised version of the manuscript, we provide quantification for data concerning Shroom 3. Specifically, we quantified NTC rate in shroom3 morphant embryo (Supplementary Figure

© 2022. Published by The Company of Biologists under the terms of the Creative Commons Attribution License (https://creativecommons.org/licenses/by/4.0/).

6B, below) and the apical cell surface area over time at the posterior and anterior neural plate of shroom3 morphant embryos (Supplementary Figure 6C, below).

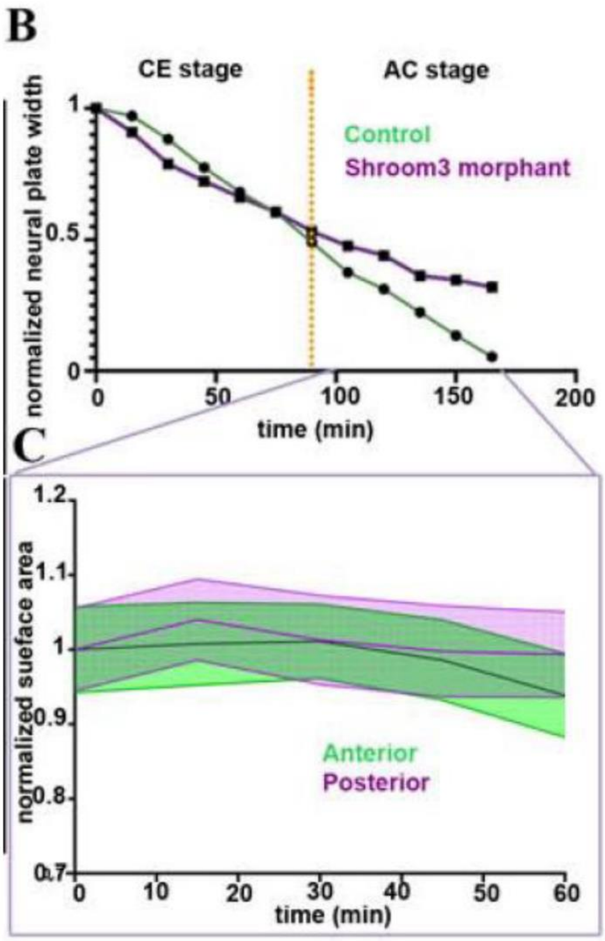

Additionally, we have quantified SE cell velocity in Shroom3 morphant embryos (Figure 5J, below).

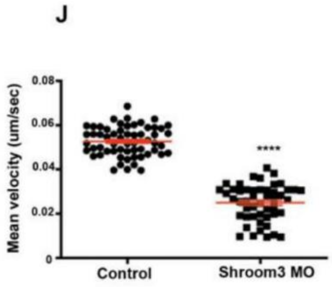

4. Likewise, the data in Fig. 5K must be explained, as the current presentation leaves them uninterpretable. No specifics are given for what the arrows or the colors in this chart represent, or what the upper and lower panels show. The same is true for Fig. 6H. In addition, no statistics or N numbers are provided for either figure. Are these PIV from a single sample, or do these somehow represent multiple samples. Making these data 1) convincing and 2) understandable is central to the paper, as the authors are concluding precisely the opposite of a previous paper (Morita, 2012).

Qualitative and quantitative data from Figure 5K and 6H are now presented in Figure 6 and 8 respectively. Additionally, we present qualitative data for the embryos used to generate the quantitative data in Figures 6 and 8 in Supplementary Figure 8 and 11 respectively.
The coloured arrows represent coloured coded normalised movement vector magnitude in all Figures. In Figure 6 we present quantitative data regarding the normalised vector magnitude of

© 2022. Published by The Company of Biologists under the terms of the Creative Commons Attribution License (https://creativecommons.org/licenses/by/4.0/).

the surface ectoderm before and after ATP unchanging within the neural plate from 3 different embryos. In Figure 8 we present quantitative data regarding the angle of the surface ectoderm movement vector before and after ATP unchanging within the surface ectoderm.
We feel that the data as presented in the revised manuscript (Figures 6,8, Supplementary Figure 8 and 11) are now convincing and understandable. These data with the data presented in Figure 5 show clearly that surface ectoderm moves passively during NTC in contrast to what was reported in a previous study (Morita, 2012).

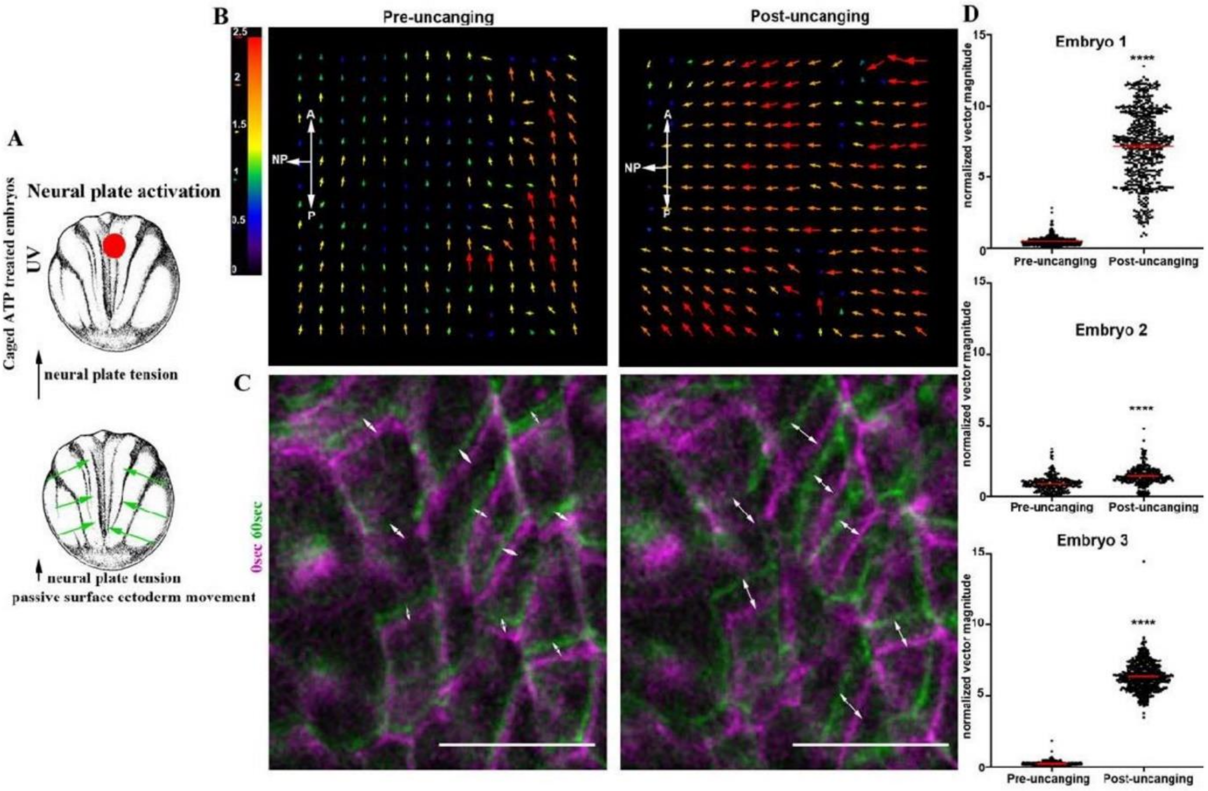

Figure 6

© 2022. Published by The Company of Biologists under the terms of the Creative Commons Attribution License (https://creativecommons.org/licenses/by/4.0/).

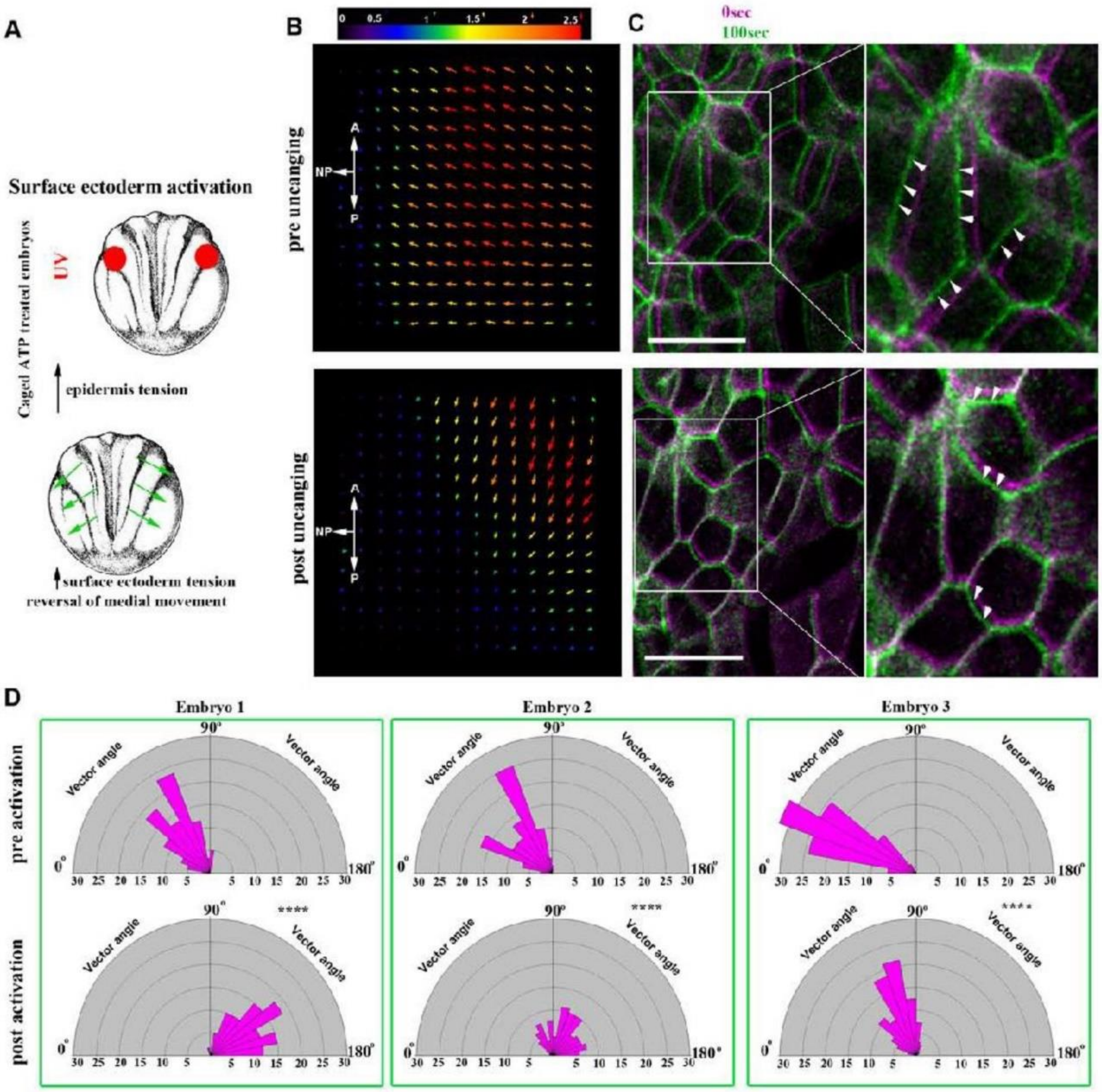

Figure 8

5. the data for Fig. 6N, showing planar polarization is maintained must be quantified. The images are not sufficient.

According to the Reviewer's comment we have quantified PK2-GFP localisation and provide this data in Figure 9E(below).

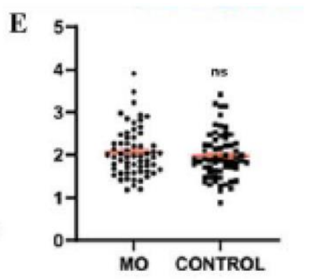

Minor comments:

1. Anterior in three different orientations in Fig 1 alone (top, bottom or left depending on the panel? The reader will be helped by picking one orientation and sticking with it for the whole paper. (Another example: Fig. 2A shows Anterior up and Posterior down within the panels, yet the

© 2022. Published by The Company of Biologists under the terms of the Creative Commons Attribution License (https://creativecommons.org/licenses/by/4.0/).

posterior panel is on top.). I am sorry to be picky, but this is a very complex paper and your readers will thank you if you make it easier!

We agree with the reviewer that consistent presentation of the embryo orientation throughout the figures will help the readers. We have revised all the figures so that the anterior region of the embryo is facing the top.

2. Keller, Shih, and Sater 1992 (PMID: 1600240) must be cited in discussion of Figure 1B (line 144) and line 180, as this precise pattern of movement was reported in that paper. Probably should cite that work in the Intro as well.
We now cite this work in the discussion of Figure 1B.

3. The method of quantification for Fig. 2C should be explicitly stated. If I read it right, the values are the ratio of AP and ML junctions, but that is
never stated and how AP and ML are decided is not reported. Ditto for Fig. 3B.
We now describe the method of quantification for ML junction enrichment in the methods sections of the revised manuscript. AP junctions were set as the junctions oriented >45 relative with the ML axis and ML junctions were set as the junctions oriented <45 relative to the ML junction.

4. Line 199. I am not sure the word “stems” is appropriate here as it implies cause, but only correlation is shown.
We revised the manuscript accordingly.

5. I do not understand the schematics at the right of fig. 4A. How is “+/- AC” “or +/-CE” determined? Is this subjective? What is this meant to convey? Also, it seems that the shape of the tissue is being used to assess whether CE or AC is active. This is not a convincing argument, only cell-level quantification such as that in panel D or G is acceptable. Maybe best to just remove this analysis? Or explain it adequately.
This analysis has now been removed from the Figure

5. I think it’s crucial to explicitly state where the data from Fig. 4E-H are taken from, anterior or posterior? Also the explanation for Panel D seems to be missing from the fig. 4 Legend. Please proofread the legend.

The data from Figure 4E-H are taken from the posterior neural plate. This is now stated in the Figure itself and in the text of the revised manuscript. We apologise for the missing legend. We now have revised the legend for Figure 4.

Reviewer 3 Comments for the Author...

This is an interesting study, which also significantly advances our understanding of the mechanical underpinnings of vertebrate neural tube closure and will be a useful addition to the field. However, for the manuscript to be considered suitable for publication in Development, the authors should address the following comments.

We thank the reviewer for the valuable suggestions that we feel that they strengthen and solidify our initial findings.

Major comments
1. One major conclusion the authors present in this manuscript is that the anterior movement of the rostral neural plate is passive and that it occurs as convergent extension of the caudal neural plate pushes it toward the anterior. This conclusion is mainly based on the analysis of Vangl2 MO embryos. Embryos with a downregulation of Vangl2, which lack caudal convergent extension movements, show limited anterior movement of the rostral neural plate, suggesting that the rostral movement is passive. However, the authors do not account for the possibility that a homogenous knockdown of Vangl2 may have other effects. For instance, could Vangl2 be directly required within the rostral neural plate to promote its active anterior movement?
The authors postulated that if the rostral cells actively move anteriorly, and convergent extension

© 2022. Published by The Company of Biologists under the terms of the Creative Commons Attribution License (https://creativecommons.org/licenses/by/4.0/).

in the caudal region is permissive for this movement, then the anteroposterior junctions of the cells at the boundary between the rostral and the caudal regions should elongate in Vangl2 MO embryos. However, the authors did not find this to be true, therefore further strengthening their conclusion. However, could the boundary cells in Vangl2 MO be stiff/tensed, thereby resisting changes in geometry despite experiencing a pulling force from the rostral cells? How do these cells look like in wild type embryos? If the anterior movement of the rostral region was inhibited (example, by introducing a physical barrier), would the boundary cells in wild type begin to exhibit signs of compression due to the anterior movement of the caudal region? Alternatively, in explants, if the rostral neural plate is replaced by a gel, would the gel also move anteriorly? As anoer option, could rostral neural plate cells, explanted and devoid of caudal cells, migrate?

We agree with the reviewer that we don't have spatial control over Vangl2 downregulation within the neural plate. With the current available tools this is not possible. However, we believe that since convergent extension only takes place at the posterior, and Vangl2 does not affect AC the defects observed in anterior neural plate movement stem from inhibition of CE at the posterior.
However, to directly address their concerns we have now followed their excellent suggestion to use explants and embryo manipulations to examine the input of the posterior neural plate morphogenesis on anterior neural plate movement and the intrinsic capacity of the neural plate to migrate.
Using anterior neural plate explants, initially we show that at the time when the posterior explant undergoes convergent extension the anterior explants do not display active migration supporting our initial conclusion that anterior neural plate movement is passive and dependent on posterior neural plate deformation. Second, we mechanically decoupled the anterior and posterior neural plate through the generation of a deep wound at the forebrain-midbrain boundary region. This manipulation again showed that anterior neural plate movement cannot take place if the mechanical interaction with the posterior tissue is disrupted. Last, to exclude the possibility that anterior neural plate explants fail to move due to absence of signals from the ventral surface ectoderm we removed the posterior neural plate from neural stage embryos to generate anterior neural plate/surface ectoderm explants. In this setting the anterior neural plate also failed to display movement further supporting our conclusion on the passive nature of anterior neural plate movement and the necessity of force generation by the posterior.
These data are now presented in Figure 2D-E and Supplementary Figure 4.

© 2022. Published by The Company of Biologists under the terms of the Creative Commons Attribution License (https://creativecommons.org/licenses/by/4.0/).

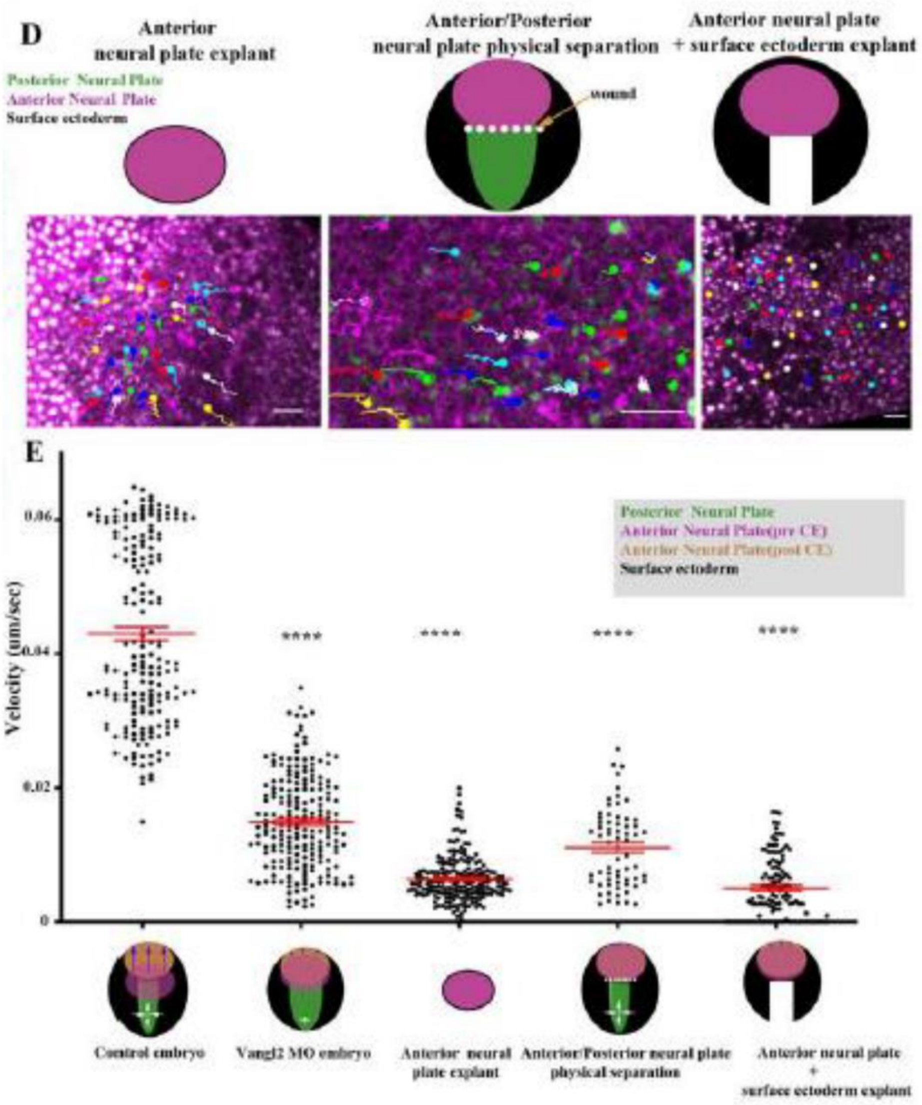

Figure 2D-E

© 2022. Published by The Company of Biologists under the terms of the Creative Commons Attribution License (https://creativecommons.org/licenses/by/4.0/).

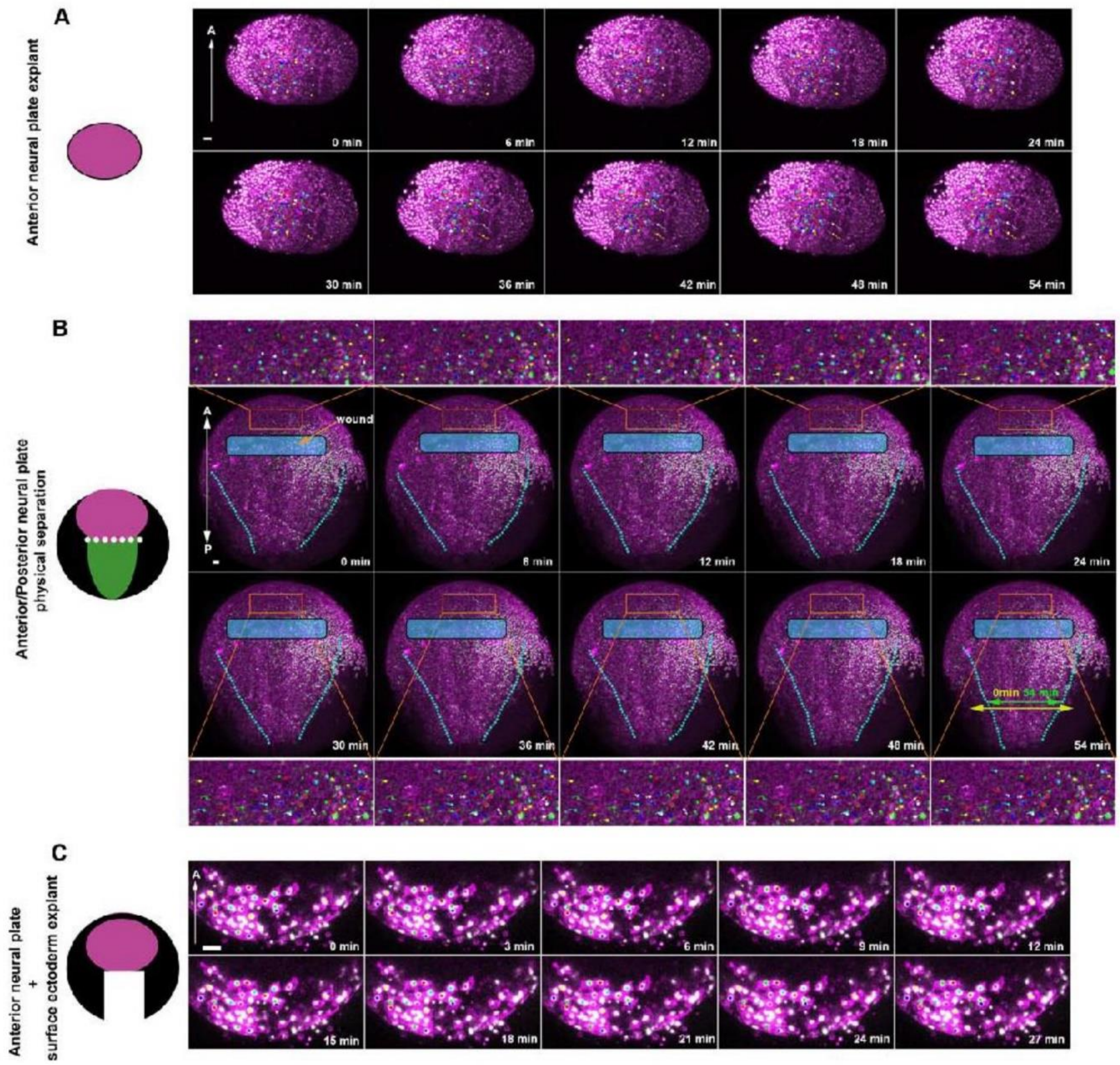

Supplementary Figure 4

2. Cells in the neural plate in Shroom3 MO embryos do not undergo apical constriction, a behavior that is exhibited by all cells in the neural plate in wild type. Yet defects in neural tube closure are only restricted to the rostral region. The authors explain this by showing that caudal convergent extension movements continue in Shroom3 MO embryos till significantly later than in wild type. They suggest that this prolongation of convergent extension compensates for the lack of apical constriction. However, it is unclear if, overall, in Shroom3 MO, there is more convergent extension or just a reduction in its rate. For instance, do the same number of neighbor exchange events occur per hour in wild type and Shroom3 MO? For convergent extension to compensate for the lack of apical constriction, one would expect there to be an overall increase in (and not just a delay in the completion of) convergent extension. Furthermore, is development overall slower in Shroom3 MO? In other words, are 60 min for wild type also equal to 60 min for Shroom3 MO?

To respond to the reviewer's concern, we quantified NTC rates in Control and Shroom3 morphant embryos (Supplementary Figure 6B). This analysis revealed that the NTC rate during the CE phase is similar between control and morphant embryos . This shows that inhibition of AC does not impact the rate of CE but the first phase is extended and just as the reviewer suggests the total number of cell intercalation events taking place is increased. In agreement with this analysis recent work showed that T1 transition events are not affected in the absence of Shroom3, with a mild effect being present in the orientation of junction remodelling events(Baldwin et al., 2022). Thereafter, the comprehensive analysis performed shows that the development of the embryos is not delayed prior to initiation of AC in Shroom3 morphants.

© 2022. Published by The Company of Biologists under the terms of the Creative Commons Attribution License (https://creativecommons.org/licenses/by/4.0/).

3. In fig. 5, the authors show that explanted surface ectoderm cells do not show migratory behavior. Based on this, they argue that surface ectoderm cells do not have the capacity to migrate during neurulation. However, could it be that important factors required for ectoderm cell migration are missing in culture? I am not sure what can be used here as a positive control but perhaps one may be required. Alternatively, on lines 296 and 297, the authors conclusion should be qualified with this caveat.
We agree with the reviewer that important factors required for ectoderm cell behaviour might be missing from cell culture. However other migratory cell types from Xenopus embryos such as the head mesoderm and cells of the neural crest attach, spread and migrate when cultured on FN substrates in-vitro. This coupled with the fact that the tissue is explanted during neurulation and subsequent to any signalling events required suggests that if these cells had the capacity to migrate, they would or at the very least attach and spread. However we can't exclude the possibility that the lack of some essential factors is responsible for this so, we revised the text stating "This revealed that deep SE cells cannot migrate when cultured ex-vivo on FN substrates as other migratory cell types do , such as the head mesoderm during gastrulation and neural crest explants during neurulation".

Furthermore, the authors also present evidence to suggest that embryos lacking morphogenetic movements within the neural plate show an absence of movement of the surface ectoderm. Based on this, they conclude that surface ectoderm movements are passive, and a result of mechanical coupling with the moving cells of the neural plate. It may be useful to at least discuss the possibility that the lack of free space in such embryos (as neural plate cells do not move forward) could be the reason for the lack of ectoderm movements.
Our data show that inhibition of both neural plate convergent extension and apical constriction affect surface ectoderm movement. If the lack of free space was the reason for defective surface ectoderm movement, then we would expect to observe compression and buckling of the surface ectoderm epithelium near the boundary with the neural plate, but we don't observe this. Furthermore, if the surface ectoderm was generating forces by active migration, then we would expect to observe bucking of the neural plate epithelium when neural plate convergent extension was inhibited which we don't observe. The above argue against the possibility that the defective surface ectoderm movement in embryos with defective neural plate morphogenesis are due to lack of free space.

While it is true that the authors have shown later that optogenetically generated pulling forces within the neural plate can pull the surface ectoderm cells (and vice versa), those experiments only show that such pulling forces are sufficient to achieve ectoderm movement, and not that they are necessary for the same. This should be made clear in the main text.
We agree with the reviewer that the optogenetic experiments only show that pulling forces from the neural plate are sufficient for surface ectoderm translocation and we state this clearly in the revised text. The necessity of neural plate morphogenesis is essential for surface ectoderm movement is supported by our analysis on surface ectoderm movement in Vangl2 and Shroom3 morphant embryos.

Minor comments
Line 139: Histone-RFP should instead be Histone-GFP, as it is in fig. 1B. In general there are a few errors in the text and the figures. Some of them have been mentioned below. The manuscript requires thorough proofreading.
Revised according to the reviewer's comment.
Line 143: How were neuroepithelial cells distinguished from surface ectoderm cells based on uniform H2B labelling? Was this done based on their morphological features? This information should be briefly included.
Using membrane fluorescent signal, we identified neural hinge points (boundary between neural plate and surface ectoderm) when the neural plate started to bend. Subsequently neuroepithelial cells were distinguished from surface ectoderm cells based on retrograde tracking.

Lines 157 and 158: The cell division angle data originates from only two embryos. A couple more embryos should be examined to determine if the observations are consistent. In addition, the values mentioned in these lines use a comma for a decimal, which is inconsistent with the way values have been represented in the rest of the manuscript.

© 2022. Published by The Company of Biologists under the terms of the Creative Commons Attribution License (https://creativecommons.org/licenses/by/4.0/).

We now include data from 5 embryos and according to Reviewer one values are provided without decimal.

Line 159: To support the claim that cell proliferation rates in the rostral and the caudal regions of the neural plate are similar, the authors have only shown two images, one each from early and late stage of development. Perhaps a graph summarizing the population data would be necessary to include.

We now include a graph summarising the data in Figure 1I (see below).

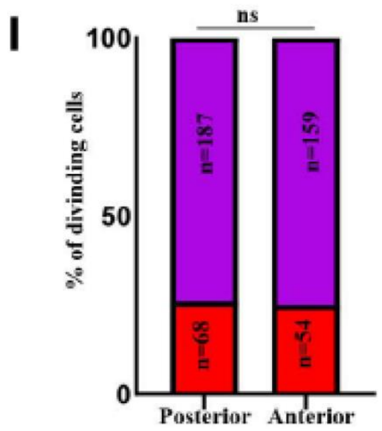

Line 225: To determine if the rostral cells pull the caudal cells, the authors have analyzed the geometry of cells at the boundary between the two regions. How were the boundary cells defined, considering that there is no clear demarcation (rather just a gradual narrowing) between the two regions?

In our analysis the boundary was defined through the tracking of cell intercalation events and marking the transition zone where such events were taking place and where they did not. These data are now presented in Figure S4D.

Lines 344-346: Information with respect to the sample size and the population data are missing. Were any paired statistical analyses performed at the population level to determine if the effect of ATP uncaging within the neural plate on the surface ectoderm was consistent and can be considered significant?

We now include statistical analysis in the revised manuscript, normalised vector magnitude and vector angles, in Figures 6 and 8 respectively.

Line 381: ‘Anteroposterior’ should actually be mediolateral, as the authors have examined the shrinkage of junctions along the mediolateral axis. That would then also be consistent with the data represented in Fig. 6J, L, and M.

We apologise for this mistake. Anteroposterior has been replaced with mediolateral in the revised manuscript.

Figures

Across all figures, it would be best to use the same embryo orientation to avoid confusion. For example: the orientations of the embryo in figs. 1B and 1D are opposite to each other.

Embryo orientation is now consistent across all figures with the anterior of the embryo at the top.

Fig. 1E: Column headings (Rostral vs Caudal) are switched.

This has now been rectified.

Fig. 2A: Labels for the double-headed arrows are switched in the top row (0 min vs 60 min).

This has now been rectified.

Fig. S2C, last image: the time labels for the double-headed arrows (165 min vs 225 min are switched).

This has now been rectified.

Fig. 3C: Embryo orientation should be indicated.

Embryo orientation has been included.

Fig. 5G: Labels for the anteroposterior labelling are cropped.

© 2022. Published by The Company of Biologists under the terms of the Creative Commons Attribution License (https://creativecommons.org/licenses/by/4.0/).

Figure revised.
Fig. 5K: Labels for images, similar to that for fig. 6K, are missing.
Images from 5K and 6K are now presented in Figures 6 and 8 respectively.
In general, quantifications and detailed information regarding the sample size and statistics should be included for all experiments wherever possible. For instance, in fig. 2C, where three timepoints for each group have been compared, it is assumed that a multiple comparisons correction must have been made. However, the corresponding information (for example, which correction test was applied) is missing.
We have included the information on sample size and statistical tests in the Figure legends. In Figure 2C we didn't perform multiple comparisons. We compared each column with the previous time point in order to understand if there is a statistically significant reduction of mediolateral junction length over time.

References:
Baldwin, A. T., Kim, J., Seo, H. and Wallingford, J. B. (2022) 'Global analysis of cell behavior and protein localization dynamics reveals region-specific functions for Shroom3 and N- cadherin during neural tube closure', *Elife* 11.

Second decision letter

MS ID#: DEVELOP/2021/200358

MS TITLE: Distinct spatiotemporal contribution of morphogenetic events and mechanical tissue coupling during Xenopus neural tube closure

AUTHORS: Neophytos Christodoulou and Paris A Skourides

I have now received all the referees reports on the above manuscript, and have reached a decision. The referees' comments are appended below, or you can access them online: please go to BenchPress and click on the 'Manuscripts with Decisions' queue in the Author Area.

The overall evaluation is very positive and we would like to publish your manuscript in Development. Before we can proceed further though, please proof-read your manuscript to correct typos as mentioned by one of reviewer 3. As I understand it, the major concerns of reviewer 1 have been addressed in your previous revision. Please address all other minor issues.

Reviewer 1

Advance summary and potential significance to field

The manuscript entitled “Distinct spatiotemporal contribution of morphogenetic events and mechanical tissue coupling during Xenopus neural tube closure” by Christodoulou and Skourides investigates the sequence in which the known cell behaviors necessary for neural tube closure, convergent extension and apical constriction, occur in the anterior and posterior neural plate during neural tube formation. Through live imaging and loss of function approaches the authors conclude that convergent extension precedes apical constriction in the posterior neural plate while the anterior neural plate passively translocates ventroanteriorly, before going through apical constriction and neural tube closure. The study also shows that the non-neural ectoderm moves passively following the forces that the neighbor neural plate exerts while folding, and that an aberrant increase in the tension generated in non-neural ectodermal cells opposes to the neural plate cell behaviors of convergent extension and apical constriction.

This study contributes to the understanding of the cellular events necessary for neural tube formation, which may be relevant to understanding the occurrence of neural tube defects and the devising of potential therapeutics for preventing them.

© 2022. Published by The Company of Biologists under the terms of the Creative Commons Attribution License (https://creativecommons.org/licenses/by/4.0/).

Comments for the author

The manuscript entitled “Distinct spatiotemporal contribution of morphogenetic events and mechanical tissue coupling during Xenopus neural tube closure” by Christodoulou and Skourides investigates the sequence in which the known cell behaviors necessary for neural tube closure, convergent extension and apical constriction, occur in the anterior and posterior neural plate during neural tube formation. Through live imaging and loss of function approaches the authors conclude that convergent extension precedes apical constriction in the posterior neural plate while the anterior neural plate passively translocates ventroanteriorly, before going through apical constriction and neural tube closure. The study also shows that the non-neural ectoderm moves passively following the forces that the neighbor neural plate exerts while folding, and that an aberrant increase in the tension generated in non-neural ectodermal cells opposes to the neural plate cell behaviors of convergent extension and apical constriction.
This study contributes to the understanding of the cellular events necessary for neural tube formation, which may be relevant to understanding the occurrence of neural tube defects and the devising of potential therapeutics for preventing them.
The authors have satisfactorily addressed some of the concerns raised in the original submission. However some remain unresolved.

Major issues:

1) The issue of targeting exclusively non-neural ectoderm by injecting ventral blastomeres of 4-cell stage embryos remains unsolved. This approach is not specific enough and some of the data provided by the authors actually document this lack of tissue specificity using this approach. Indeed, images in Figures S2, S9 and 7 provide evidence that either additional tissues or not the intended tissue are targeted with the 4-cell stage injections. And this is not surprising; cell fate mapping presented in Xenbase shows how ventral nervous system structures are majorly contributed by ventral blastomeres.
Similarly for the non-neural ectoderm receiving contribution from dorsal blastomeres (xenbase.org).
Microinjections at the 8-cell stage can give better specificity.
For instance, non-neural ectoderm is also targeted by dorsal blastomere injections at 4-cell stage as shown in Figure S2B, although the image is cropped on the right to appreciate the full extent of the non-neural ectoderm expressing the injected construct.
Similarly, in Figure S9B, the zoomed images do not include the neural ectoderm, so it is not possible to appreciate the exclusivity of the targeting of the non-neural ectoderm.
Moreover, Figure 7B clearly shows how the ventral blastomere injection is also contributing to the neural ectoderm, and it misses to target the superficial layer of cells of the non-neural ectoderm next to the neural plate, which are presumably those affecting the morphogenetic changes of the neural plate.
It is also unclear what regions of the non-neural ectoderm are shown zoomed-in in Figure 7C. They seem to correspond to areas very distant from the neural plate, so it is unclear how a change in these areas can influence the neural plate. Instead, it is more likely that the manipulation of gene expression in neural plate cells due to the 4-cell stage injections, is responsible for the changes in its morphogenesis.
With the approach of 4-cell stage injections that the authors chose, I do not think is possible or appropriate to make strong conclusions about the influence of the non-neural ectoderm on neural plate morphogenesis and closure.

2) An additional control is needed for the ATP-uncaging experiment that will report on the effect of the UV-photo-stimulation without the caged-ATP. Additionally, details on what objective/microscope was used for the uncaging are needed in Methods.

3) Moreover, I remain concerned that a clearer evidence of the spatial resolution achieved by the uncaged ATP is still missing in this manuscript. The use of mEOS to demonstrate spatial resolution for the uncaging of ATP is problematic because the former is a photo-conversion of a protein expressed by the cells in the illuminated region. But the caged-ATP is applied to the bath. Thus, when uncaged, it will diffuse beyond the illuminated region. Ideally, the authors would show how ATP signaling is affected in a delimited region or they would use a probe that is more comparable to the caged/uncaged ATP to demonstrate the spatial/tissue specificity.

© 2022. Published by The Company of Biologists under the terms of the Creative Commons Attribution License (https://creativecommons.org/licenses/by/4.0/).

4) In Figure 9B,D the reported lack of reduction in mediolateral junction length in the affected neural plate could be due to an overall failure of apical constriction, ie deficient reduction of apical surface of neural plate cells, unlike in the control side (which indeed shows advanced apical constriction compared to affected side of the neural plate), and not because of failed convergent extension, as the authors argue. To convincingly show that convergent extension failed, they should examine ML junction length at earlier time points when apical constriction has not occurred yet.

Minor issues:

5) Shouldn't the color-code in labels in Figure S1C (0 and 135 min) be swapped?

6) Figure legend S5 needs a title

7) The presentation of data in Figure S5C is very confusing and needs revision.

8) Some sentences need revision, ie, 225-227; 245-249.

9) It is unclear what the authors mean by “surface ectoderm homeostasis” as a permissive factor in neural tube closure. It seems an inadequate term in this context, or it needs further clarification.

Reviewer 2

Advance summary and potential significance to field

My comments have been adequately addressed in this revision and i strongly support publication at this stage.

Comments for the author

Great work!

Reviewer 3

Advance summary and potential significance to field

-

Comments for the author

The authors have responded to our previous comments to my satisfaction. I just have two very minor comments.
1. In the very last point in their response, they say that they don't require a multiple comparisons correction for the data shown in fig. 2C, whereas I think that they do... although that wouldn't change the statistical relationships or their conclusion.
2. There are still some minor errors that should be rectified. The manuscript requires some proofreading. e.g. a spelling mistake 'sueface' in the y-axis title in fig. S6D. In this same panel, the outline of the plot is cropped.
Otherwise, I am fully satisfied with the revisions and think the the manuscript is suitable for publication in Development.

© 2022. Published by The Company of Biologists under the terms of the Creative Commons Attribution License (https://creativecommons.org/licenses/by/4.0/).

Second revision

Author response to reviewers' comments

Reviewer 1

Minor issues:

5) Shouldn't the color-code in labels in Figure S1C (0 and 135 min) be swapped?
The labels in Figure S1C have been swapped.

6) Figure legend S5 needs a title
We have added a title for Figure S5 in the revised manuscript

7) The presentation of data in Figure S5C is very confusing and needs revision.
We don't have Figure S5C in the manuscript

8) Some sentences need revision, ie, 225-227; 245-249.
Sentences have been revised

9) It is unclear what the authors mean by "surface ectoderm homeostasis" as a permissive factor in neural tube closure. It seems an inadequate term in this context, or it needs further clarification.
'Homeostasis' was replaced by 'development'.

Reviewer 3

1. In the very last point in their response, they say that they don't require a multiple comparisons correction for the data shown in fig. 2C, whereas I think that they do... although that wouldn't change the statistical relationships or their conclusion.
   We perform multiple comparison (Two-way ANOVA test (for the data presented in Figure2c as suggested by the reviewer. This does not change the statistical relationship in the data and our conclusions.

2. There are still some minor errors that should be rectified. The manuscript requires some proofreading. e.g. a spelling mistake 'sueface' in the y-axis title in fig. S6D. In this same panel, the outline of the plot is cropped.

   We have proofread the manuscript and the spelling mistake in FigureS6D has been corrected.

Third decision letter

MS ID#: DEVELOP/2021/200358

MS TITLE: Distinct spatiotemporal contribution of morphogenetic events and mechanical tissue coupling during Xenopus neural tube closure

AUTHORS: Neophytos Christodoulou and Paris A Skourides
ARTICLE TYPE: Research Article

I am happy to tell you that your manuscript has been accepted for publication in Development, pending our standard ethics checks.

© 2022. Published by The Company of Biologists under the terms of the Creative Commons Attribution License (https://creativecommons.org/licenses/by/4.0/).
